# Uncovering the Mechanism of Curcuma in the Treatment of Ulcerative Colitis Based on Network Pharmacology, Molecular Docking Technology, and Experiment Verification

**DOI:** 10.1155/2021/6629761

**Published:** 2021-06-16

**Authors:** Suxian Liu, Qiaodong Li, Fengzhi Liu, Hui Cao, Jun Liu, Jingyi Shan, Wenchao Dan, Jianye Yuan, Jiang Lin

**Affiliations:** ^1^Longhua Affiliated Hospital of Shanghai University of Traditional Chinese Medicine, Shanghai 200032, China; ^2^Department of Gastroenterology, China–Canada Center of Research for Digestive Diseases, Institute of Digestive Diseases, Longhua Hospital, Shanghai University of Traditional Chinese Medicine, Shanghai 200032, China; ^3^Key Laboratory of Chinese Internal Medicine of Ministry of Education and Beijing, Dongzhimen Hospital, Beijing University of Chinese Medicine, Beijing 100700, China; ^4^Beijing University of Chinese Medicine, Beijing 100029, China; ^5^Department of Cardiology, China Academy of Chinese Medical Sciences Guanganmen Hospital, Beijing 100053, China

## Abstract

**Aim:**

The incidence of ulcerative colitis (UC) is increasing steadily in developed countries, it is plaguing nearly 1 million people in the United States and European countries, while developing countries have had a rapidly increased incidence over the past decades. Curcuma is widely used in treating malaria, UC, Crohn's disease, and colon cancer, which lead to diarrhea and bloody stool. However, the systemic mechanism of curcuma in treating UC is still unclear. Our work was supposed to expound how does curcuma alleviate UC in a comprehensive and systematic way by network pharmacology, molecular docking, and experiment verification.

**Methods:**

Traditional Chinese Medicine System Pharmacology Database (TCMSP), Shanghai Chemistry & Chemical Industry Data Platform (SGST), and papers published in Chinese Network Knowledge Infrastructure (CNKI) and PubMed were used to collect the chemical constituents of curcuma based on ADME (absorption, distribution, metabolism, and excretion). And effective targets were predicted by Swiss Target Prediction to establish the curcuma-related database. The disease targets of UC were screened by GeneCards and DrugBank databases, and Wayne (Venn) analysis was carried out with curcuma targets to determine the intersection targets. AutoDock software and TCMNPAS system were used to dock the core chemical components of curcuma with key UC targets. Protein interaction (PPI) network was constructed based on the STRING database and Cytoscape software. Gene function GO analysis and KEGG pathway enrichment analysis were carried out by using Metascape database. Finally, HE staining was performed to identify the inflammatory infiltration and expression difference in TNF-*α* and STAT3 before and after the treatment of curcuma which was verified by immunoblotting.

**Results:**

Twelve active components containing 148 target genes were selected from curcuma. Potential therapeutic targets of curcuma in the treatment of UC were acquired from 54 overlapped targets from UC and curcuma. Molecular docking was used to filter the exact 24 core proteins interacting with compounds whose docking energy is lower than −5.5 and stronger than that of 5-aminosalicylic acid (5-ASA). GO and KEGG analyses showed that these targets were highly correlated with EGFR tyrosine kinase inhibitor resistance, PI3K-Akt signaling pathway, JAK-STAT signaling pathway, MAPK signaling pathway, and inflammatory bowel disease (IBD). Experiments verified curcuma relieved pathological manifestation and decreased the expression of TNF-*α* and STAT3.

**Conclusion:**

Curcuma relieved the colon inflammation of ulcerative colitis via inactivating TNF pathway, inflammatory bowel disease pathway, and epithelial cell signaling in *Helicobacter pylori* infection pathway, probably by binding to STAT3 and TNF-*α*.

## 1. Background

UC is characterized by abdominal pain, diarrhea, and bloody stool [1]. Globally, the annual incidence of UC is about 9–20/100,000 and the prevalence is 156–291/100,000 [2]. However, the exact pathogenesis is still not fully clear for the etiology is prototypically diverse. It is known that many factors are involved in the development of UC, interacting environmental, genomic, microbial, and immunological elements [3]. With the deterioration of UC, it will eventually lead to colorectal cancer. Therefore, to prevent before the occurrence of UC, to avoid the complications of UC, and to prevent the recurrence of UC after recovery, a new treatment is urgently needed.

TCM is gaining its popularity in the ameliorating subhealth state and treating disease. Much more attention has been focused on the treatment of UC by Chinese herb; curcuma is one of the popular ones. In ancient China, curcuma was widely used to invigorating the circulation of blood in clinical applications; nowadays, the function of the positive regulation of inflammatory cytokines in inflammatory diseases [4] and its safety [5] attracted many more clinical trials and experimental verifications.

It is well known that TCM is guided by the theory of TCM and characteristics of being multicomponents, multitargets, and multipathways in the treatment of diseases, which meets the requirements of systematically tackling complex diseases such as colorectal cancer. Guo et al. established the model of colorectal cancer and predicted the traditional Chinese medicine components of inhibiting inflammation-induced tumorigenesis by using network pharmacology method [6]. Gupta et al. gathered curcumin's pleiotropic activities from many research studies to conclude its ability to modulate numerous signaling molecules such as proinflammatory cytokines, apoptotic proteins, cyclooxygenases, and C-reactive protein in human participants [7]. To improve the “one target and one drug” mode to “network targeting multicomponent” mode and to discover traditional Chinese medicine from the perspective of system and molecular level [8], a systemic overall approach of curcuma in the treatment of UC is still needed to verify the previous results and broaden the mechanism of curcuma in the treatment of UC.

## 2. Methods

### 2.1. Network Pharmacology

#### 2.1.1. Active Compound Screening

Traditional Chinese Medicine System Pharmacology Database [9] (TCMSP, http://lsp.nwu.edu.cn/tcmsp.php), Shanghai Chemistry & Chemical Industry Data Platform (SGST, http://www.organchem.csdb.cn), and papers published in Chinese Network Knowledge Infrastructure (CNKI, https://www.cnki.net/) and PubMed were used to collect the chemical constituents of curcuma. We screened curcuma compounds based on absorption, distribution, metabolism, and excretion (ADME) [10], and pharmacokinetic information retrieval filters were used to retrieve bioactive compounds for further analysis under the conditions of OB ≥ 30% and DL ≥ 0.18 in TCMSP [9]. We further screen the active ingredients by their effects on the human body. However, the compounds were searched from CNKI, PubMed, and SGST without ADME parameters, so we obtained chemical formula of those components from PubChem (https://pubchem.ncbi.nlm.nih.gov/) to finish Swiss ADME prediction [11], which was requested that OB degree was equal to HIGH and at least two terms of druglikeness were YES [12].

#### 2.1.2. Screening of Possible Targets for Curcuma

PubChem was used to search the chemical structures of the active compounds. Potential targets of curcuma were predicted by Swiss Target Prediction (STP, http://www.swisstargetprediction.) [11]. Probability was used to balance the connection between compounds and targets, which was closer to 1, and it was more connective. We screened targets by the median of probability to establish potential target database related to curcuma.

#### 2.1.3. Predicting the Possible Targets of UC

Data of UC-associated target genes were gathered from GeneCards (https://www.genecards.org/) [13] and DrugBank [14] (https://www.drugbank.ca/) with the keyword “ulcerative colitis.” In addition, articles published in CNKI and PubMed about the known targets of its active compounds were counted [15]. Genes from GeneCards were provided with scores, and genes were selected as UC-related ones whose scores were above the median degree [16].

#### 2.1.4. Gathering Compound-Disease Overlapped Targets

The screened curcuma targets and UC targets were imported into Bioinformatics [17] (http://www.bioinformatics.com.cn/), and the overlapped targets of compound-disease were obtained as the potential targets for further analysis.

#### 2.1.5. PPI Network of Compound-Disease Overlapped Targets

Protein-protein interaction (PPI) network was derived based on the STRING database (https://string-db.org/), which covered almost all functional interactions between the expressed proteins [18]. Species were set as “Homo sapiens,” and the target interaction information was obtained according to the results of analysis.

#### 2.1.6. Gene Ontology (GO) and KEGG Pathway Enrichment Analysis

The biological process (BP), molecular function (MF), cell component (CC), and Kyoto Encyclopedia of Genes and Genomes (KEGG) pathway enrichment analysis were carried out using Metascape system (https://metascape.org/) [19]. In this research, GO functional annotation and KEGG pathway enrichment analyses were performed using the *P* value less than 0.05.

#### 2.1.7. Construction of Active Component-Target-Pathway Network

A visual network was constructed through Cytoscape software to reflect the complex relationship between active compounds, filtrated targets [17], and pathways based on KEGG pathway enrichment analysis to reflect the relationship between top pathways, included targets, and active compounds. Nodes represented the compounds, targets, and pathways, while edges indicated the interactions between pathways, targets, and components potentially included in the treatment of UC by curcuma.

### 2.2. Molecular Docking

Using TCMNPAS system [20] and AutoDock [21] software, the docking energy between the overlapped proteins and chemical ingredients of curcuma was calculated. Between the component and the target by docking score value the binding activity should meet two standards: bind tighter than 5-ASA and binding energy was lower than −5.5 to further filtrate the targets related to the treatment of UC by curcuma.

### 2.3. Experiment Verification

#### 2.3.1. Drugs and Reagents

Curcuma (TCM Pharmacy of Longhua Hospital of Shanghai University of Traditional Chinese Medicine), DSS (MP Biomedicals, USA), absolute ethyl alcohol, Tween-20, xylene substitute (Sinopharm Group Chemical Reagent Co. Ltd.), RIPA Lysis buffer, PMSF, BSA, BCA Protein Quantitation Kit (Beyotime), PAGE gel rapid preparation kit, Multicolor Restrained Protein Ladder (Shanghai EpiZyme Biotechnology Co., Ltd.), *β*-actin, anti-STAT3 antibody, anti-TNF-*α* antibody (Abcam Company, England), HE dyeing (Shanghai Yixin Biotechnology Co., Ltd.), and neutral gum (Shanghai Yiyang Instrument Co., Ltd.) were used.

#### 2.3.2. Consumables

Homogenized tube, ceramic beads, frozen storage tube (Shanghai Yike Biotechnology Co., Ltd.), centrifuge tube (Axygen Company, USA), PVDF membrane (Millipore Company, USA), and 96-well plate (Eppendorf Life Sciences Corporation) were used.

#### 2.3.3. Instruments

H2050r high-speed refrigerated centrifuge (Hunan Xiangyi Company), MIX-S vortex mixer, shaker oscillator (Shiloh, USA), TGear mini centrifuge (Tiangen), heating magnetic agitator (Dalong, Beijing), SIM-F140 ice maker (Sanyo, Japan), electronic balance (Sartorius, Germany), enzyme labeling instrument (BioTek, USA), tissue grinding homogenizer (MP Biomedicals, USA), electric constant temperature blast drying oven (Jinghong, Shanghai), electrophoretic system, transfer system, glue rack, ultralow-temperature freezer (SANYO, Japan), microtome (Laika, Germany), and TKY-BMB, electrothermostatic water bath (Hualida).

#### 2.3.4. Animals

Healthy male Sprague Dawley (SD) rats, weighing 180 ± 20 g, were provided by Charles River Experiment Technology Co., Ltd., and the certificate number is SCXK (Hu) 2017-0005. The rats were housed in the animal room of Shanghai University of Traditional Chinese Medicine.

#### 2.3.5. Preparation of Curcuma

The native herb was selected, and standard decoction pieces were prepared with reference to the Chinese Pharmacopoeia (2015 edition). Extract was prepared by boiling the samples in 8 times amount of water for 30 min. The procedure was repeated 3 times.

#### 2.3.6. Groups and the Construction of the UC Model

SD rats were accepted to the laboratory for 7 days before the experiments. According to the random number table, the rats were divided into 3 groups of 4 rats each: control, model, and curcuma. Except for the control group, the UC model was prepared with 5% DSS, and the intervention was given according to the group after 7 days.

#### 2.3.7. Drug Administration

The curcuma group was administered continuously by gavage with 2 ml 0.1 g/ml curcuma suspension for 7 days. The other two groups were given saline 1.08 g/kg.

#### 2.3.8. HE Staining and Western Blot

The steps to stain the samples are as follows: xylene I and xylene II, 10 min for each; 100% alcohol I, 100% alcohol II, 95% alcohol, and 85% alcohol, 5 min for each; water washing for 20 s, hematoxylin for 7 min, water washing for 1 min, 1% hydrochloric acid alcohol for 10 s, 50°C water washing for 5 min, eosin stain for 2 min, and water washing for 10 s; 85% alcohol, 95% alcohol, 100% alcohol II, and 100% alcohol I, each for 2 min; and xylene II and xylene I, 3 min for each. Neutral balsam was added after xylene was passerillaged.

For protein extraction, tissues were placed in homogenized tubes; 5 porcelain beads, 500 *μ*L of RIPA, and 5 *μ*L PMSF were added to each tube. After five times' homogenization, the colon tissue was basically broken. The supernatant was extracted after centrifugation to test the protein concentration and to collocate protein solution. Equivalent amounts of protein (200 *μ*l) were denatured at 98°C for 10 min in sample loading buffer, then separated by electrophoresis in 15% gel, and electrotransferred onto 0.45 *μ*m polyvinylidene difluoride membranes for 60 min at 350 mA. Subsequently, the membranes were blocked in blocking buffer (0.01 M phosphate-buffered saline, 0.05% Tween-20 with 5% skim milk) at 25 ± 5°C, followed by incubation with primary antibodies against STAT3 (1 : 5000) and TNF-*α* (1 : 5000) at 4°C overnight. After being washed with Tris-buffered saline containing Tween-20 (TBST) for 5 min three times, the membranes were incubated with a horseradish peroxidase-conjugated secondary antibody for 1 h at room temperature. After the membranes were washed three times in TBST for 20 min each time, the bands were visualized on X-ray film using an enhanced chemiluminescence western blotting (WB) detection system. The Image Lab™ software was used for quantitative analysis.

#### 2.3.9. The Whole Workflow of Network Pharmacology Strategy

**Figure 1:**
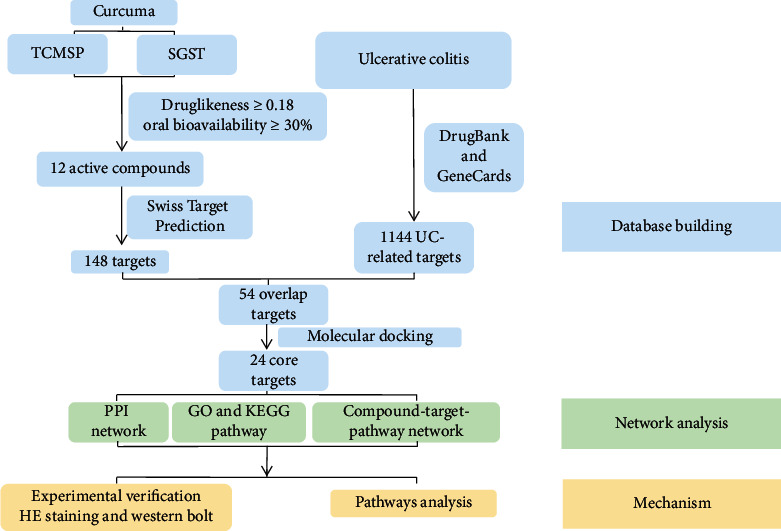
The workflow of the study. Chemical constituents of curcuma were collected from TCMSP, SGST, CNKI, and PubMed based on druglikeness and oral bioavailability. The active compounds were collected further and imported into Swiss Target Prediction to get protein targets of curcuma. Then, we obtained UC-related genes from GeneCards and DrugBank, and coincident genes from curcuma and UC were collected for molecular docking to filter the proteins binding with compounds stronger than 5-ASA. PPI network was carried out by using the STRING database. GO and KEGG pathway analyses were performed by Metascape, and compound-target-pathway network was executed by Cytoscape. Further experimental verification and pathway analysis were carried out to interpret the mechanism.

The workflow of this study is summarized in Figure 1. We built the ingredient-target collection of curcuma and UC-related genes, respectively. The overlapping targets of curcuma and UC were subsequently identified using molecular docking. And the PPI network was constructed according to the targets. Further, the gene ontology (GO) and the Kyoto encyclopedia of genes and genomes (KEGG) pathway enrichment analyses were performed and also compound-target-pathway network was executed. Next, we performed animal experiments to verify the remission of UC by admitting curcuma through HE staining and immunoblotting. Last, key pathways were analyzed to elucidate the mechanism of curcuma in the treatment of UC.

## 3. Results

### 3.1. Active Compounds and Targets of Curcuma

TCMSP database, SGST, and articles published in CNKI and PubMed were used to gather the active components and targets, and 13 potential ingredients were discovered from curcuma (Table 1). We excluded 1,7-bis(4-hydroxy-3-methoxyphenyl)-1,4,6-heptadiene-4-one and determined that 12 active components out of 13 can produce platelet aggregation and other functions in the human body, through literature review [24, 25]. Furthermore, 148 targets were affirmed through PubChem and Swiss Target Prediction.

### 3.2. Searching the Potential Disease Targets

**Figure 2:**
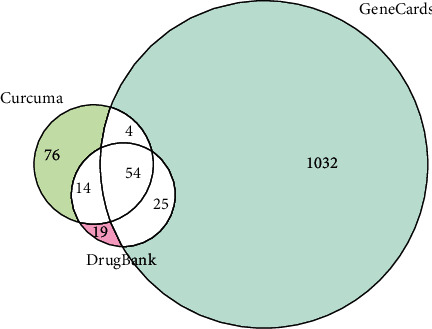
Matching of target genes between UC and curcuma.

We obtained the curcuma-related targets from GeneCards (whose relevance score is above the median point) and the ones from DrugBank, CNKI, and PubMed (Table S3). Then the searched results were overlapped with UC targets to obtain the UC-related disease protein targets (Figure 2). Fifty-four potential targets were obtained based on the degree of correlation between curcuma and UC, and their detailed information is shown in Table 2.

### 3.3. Molecular Docking

**Figure 3:**
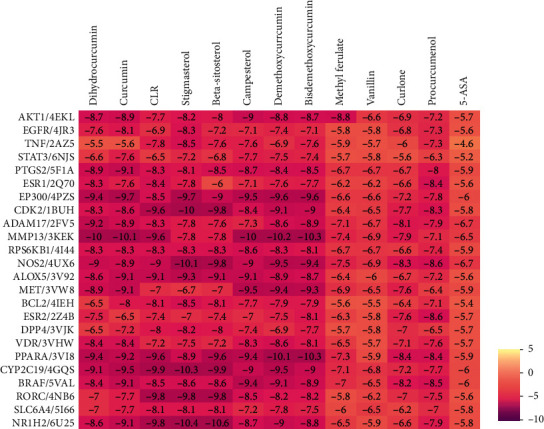
24 proteins whose docking energy with curcuma compounds was above that of 5-ASA.

We input 54 potential genes into the PDB database (http://www.rcsb.org/) to query their PDB ID [26]. Then, we downloaded the molecular structure of the 12 effective compounds of curcuma in the treatment of UC in ZINC database [27] (http://zinc.docking.org/). Molecular docking was progressed in TCMNPAS to calculate the docking score. The interaction strength between compounds and gene corresponding proteins can be expressed by docking fraction, and the lower the score is, the higher the interaction intensity is. Further, we docked the 54 potential targets with 5-ASA. As shown in Figure 3, the 24 proteins whose docking energy with curcuma compounds matched two standards, the docking energy was above that of 5-ASA and lower than −5.5, were picked for further network analysis.

### 3.4. Establishment of PPI Network

**Figure 4:**
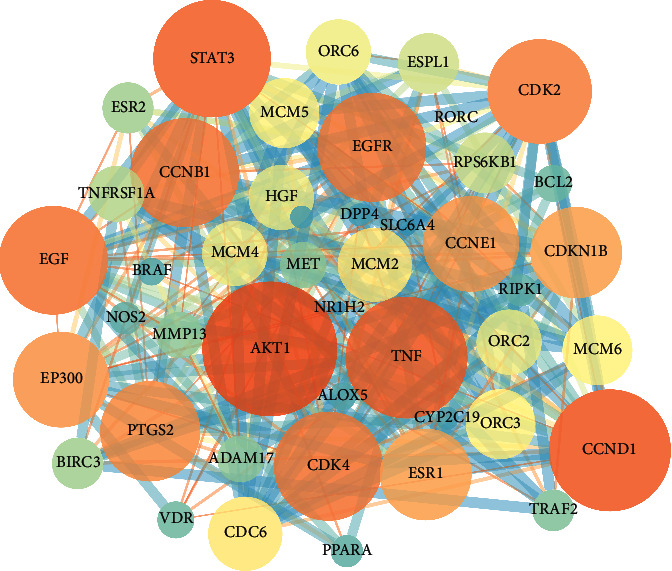
PPI network between UC and curcuma. Each node represented a target for curcuma in the treatment of UC. The smaller size and the darker color mean the lower degree value. The edges among nodes display the relationship between different targets. The larger edge size and the brighter edge color mean the higher combination scores.

The molecular docking filtered 24 core targets for curcuma in the treatment of UC. Then, the 24 genes were uploaded into the STRING database for analysis. We selected protein targets with a medium confidence score of 0.400 and the selected protein targets were plotted as an interaction network. The network of protein-protein interactions (PPI) was established through the STRING database. As shown in Figure 4, 47 nodes and 274 edges were contained in the network; in detail, the average node degree is 12.5. Nodes represented the core targets and the extended targets, edges represented the connection between the genes, and the degree value represented the association intensity.

### 3.5. Gene Ontology Enrichment Analysis and KEGG Pathway Enrichment

**Figure 5:**
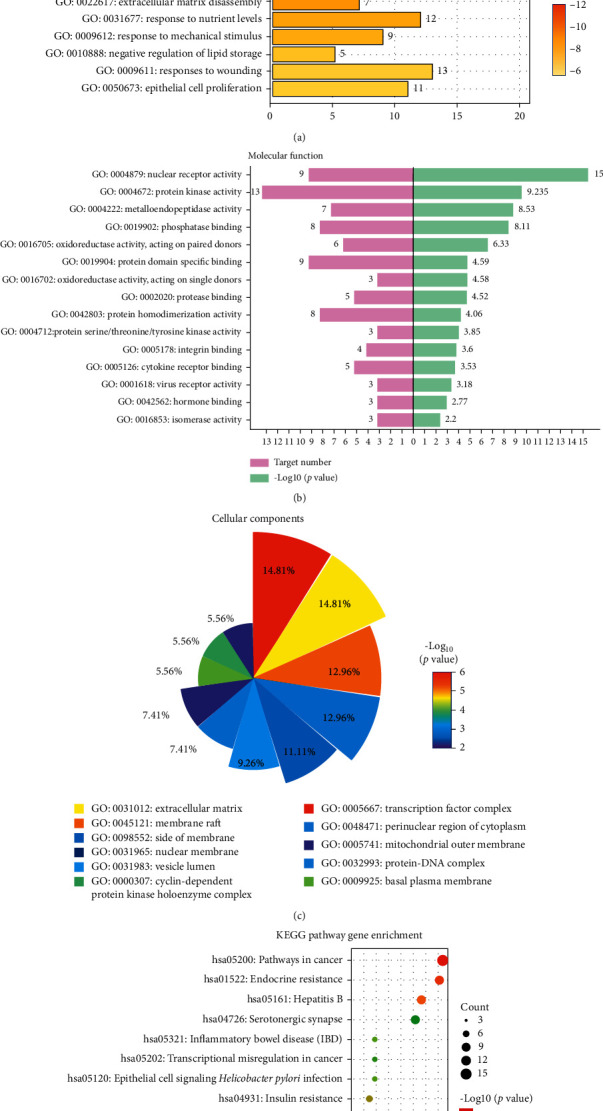
Target biological function and target-pathway analysis. (a) Biological process of curcuma in the treatment of UC. (b) Molecular function of curcuma in the treatment of UC. (c) Cellular components of curcuma in the treatment of UC. (d) Signal pathway of curcuma in the treatment of UC.

We imported the selected potential 25 target genes into the Metascape system for GO and KEGG pathway enrichment analyses. The results revealed that the functions of these potential targets were related to many biological processes, molecular functions, cellular components, and pathways, which were of crucial importance in the development and treatment of UC. A total of 886 biological processes were enriched based on *P* < 0.05, such as cellular response to lipid, response to oxygen levels, and regulation of inflammatory response (Figure 5(a)). A total of 58 molecular function GO terms were enriched (Figure 5(b)). These targets of molecular function mainly involved nuclear receptor activity, transcription factor activity, steroid hormone receptor activity, and many genes related to the molecular functions described above. In all, 18 cell component GO terms were enriched (Figure 5(c)). The targets were closely related to RNA polymerase II transcription factor complex, transcription factor complex, nuclear transcription factor complex, and membrane raft, and many targets were ranked highly as potential related genes. The biological processes, molecular functions, cellular components, and pathways revealed the vital role of curcuma in the treatment of UC.

To further reveal the potential mechanism of curcuma on the effect of UC, we conducted KEGG pathway enrichment analysis on 24 targets and screened out 95 pathways based on the threshold of *P* < 0.05(Figure 5(d)). Numerous pathways for potential target genes were identified, such as epithelial cell signaling in *Helicobacter pylori* infection related to misregulation of intestinal flora. TNF signaling pathways and inflammatory bowel disease (IBD) are closely related to the inflammatory reaction process. p53 signaling pathway is included in the pathways in cancer. Moreover, MAPK signaling pathway and TGF-beta signaling pathway play a crucial role in immunological stress. In addition, we found some other pathways such as endocrine resistance, hepatitis B, serotonergic synapse, and longevity regulating pathway, which revealed that curcuma has a potential application in other related diseases. The KEGG pathways verified that curcuma cured UC by regulating gut microbiota, inflammatory process, immunization, and inflammatory reaction.

### 3.6. Component-Target-Pathway Network

**Figure 6:**
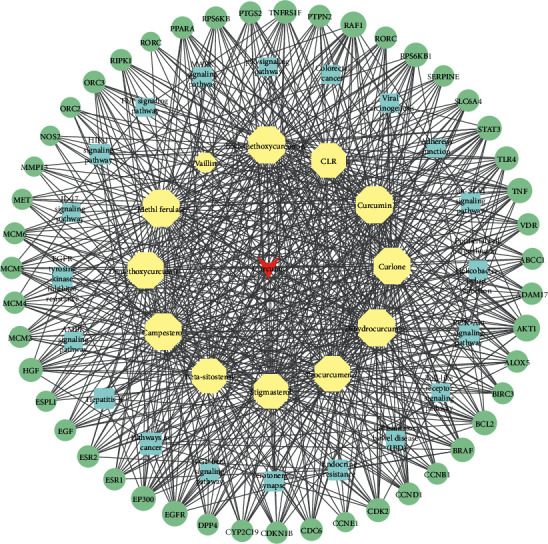
Component-target-pathway network of potential pathways in the treatment of UC by curcuma. The green colored circles correspond to the core common targets and the compounds calculated in blue were pathways that contain the targets, and the nodes in yellow are components in curcuma.

To view the relationship between the components, common targets, and their corresponding pathways, a target-pathway network was constructed (Figure 6). Twenty pathways, 47 core common targets, and 12 active compounds were connected. The network contained 88 nodes and 628 edges, in which the green colored circles represented the core common targets, the compounds colored in blue were pathways that contain the targets, and yellow nodes were effective components in curcuma. The pathways with more targets were pathways in cancer, EGFR tyrosine kinase inhibitor resistance, endocrine resistance, and HIF-1 signaling pathway, which correspond to 12, 8, 8, and 8 targets, respectively. The result suggested that these four pathways probably played significant therapeutic roles.

### 3.7. HE Staining and the Effect of Curcuma on the Expression of Key Protein in the Colon of UC Rats

**Figure 7:**
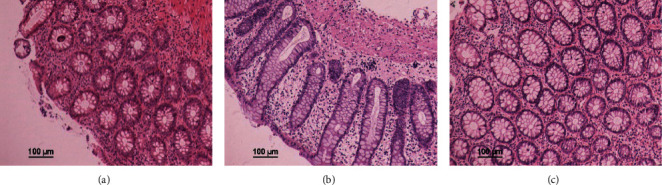
Effects of curcuma on colon tissue. (a) Control. (b) Model. (c) Curcuma.

According to the HE staining (Figure 7), DSS could change the crypt structure and inflammatory infiltration in the model rats, which were characterized by the distortion and branch of crypt structures, loss of goblet cells, appearance of a large number of crypt abscesses, lymphocytes, and plasmacyte. The above results indicated that curcuma could ameliorate DSS-induced UC in terms of colon histopathological changes.

**Figure 8:**
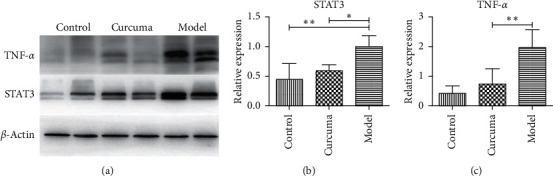
Effects of protein expression in each group (*n* = 12). ^*∗*^*P* < 0.05 and ^*∗∗*^*P* < 0.01.

Compared with the control group, the protein expression of STAT3 and TNF-*α* in the colon samples of the model group was significantly increased (*P* < 0.01). Compared with the model group, the curcuma group can reduce the expression of STAT3 and TNF-*α* (*P* < 0.05) (Figure 8).

## 4. Discussion

UC is one of the autoimmune diseases affecting northern Europe, Canada, and Australia [28]. The occurrence and development of UC are related to commensal microflora, antigen recognition, dysregulation of immunological responses, leucocyte recruitment, and genetic factors [29]. A large number of murine experiments and clinical tests have been performed to identify the bright prospects for the treatment of UC by curcuma, and TNF-*α* and STAT3 are two research hotspots. It was reported that curcumin and semibionic extraction of compound turmeric can inhibit the proinflammatory signaling by STAT3 and TNF-*α* in experimental colitis [30, 31]. Similarly, clinical research studies verified that curcumin inhibits NF-*κ*B expression by regulating tumor necrosis factor-*α* (TNF-*α*) in humans [5]. Further, meta-analyses identified that curcumin have the potential to induce and maintain remission in UC patients with no serious side effects [28].

The 12 found core compounds not only have anti-immune and anti-inflammation effects but also have the effect of regulating intestinal flora. Scholars verified that the extension of UC is important for the positivity rate of *H. pylori* later [32]. Moreover, it was reported that curcumin downregulated the expression of tumor necrosis factor-*α* (TNF-*α*) through inhibiting NF-*κ*B expression [32]. Meanwhile, curcumin enhanced the suppressive function of Treg cells and promoted the recovery of damaged colonic mucosa in UC [33]. Previous studies also suggested that CLRs revealed in the research can be included in many immune responses [34].

Molecular docking verified that the binding energy of 24 key targets was better than that of 5-ASA and lower than −5.5, meaning the interaction between the compounds and targets of UC can bind tighter than that of 5-ASA, indicating better therapeutic effects. Common target PPI network showed that the targets were cocontrolled by curcuma and UC, which revealed that curcuma could regulate the expression of UC-regulated targets and alleviate UC symptoms. AKT1 (degree = 43), EGFR (degree = 35), TNF-*α* (degree = 35), STAT3 (degree = 33), and PTGS2 (degree = 32) might be the core targets of the PPI network, through whom curcuma may come into play.

In order to predict the mechanism of curcuma in the treatment of UC, we analyzed the key candidate targets by performing GO enrichment results, such as biological processes, molecular functions, and cellular components. The GO terms (*P* < 0.05) indicated that the major hubs were significantly involved in multiple biological processes, including cellular response to lipid, cellular response to organic cyclic compound, and response to steroid hormone. Furthermore, molecular function enrichment analysis showed nuclear receptor activity, transcription factor activity, and steroid hormone receptor activity were contained in the healing process. The active targets involved were AKT1, EGFR, TNF, STAT3, and PTGS2, which mainly concentrate on the molecular processes of immunization, inflammatory reaction, gut microbiota, etc. Some of the genes and mechanisms have been verified in curcumin, while PTGS2 was proposed connecting with the treatment of UC by curcuma firstly as a gene with high possibility. Meanwhile, cellular components consist of RNA polymerase II transcription factor complex, transcription factor complex, nuclear transcription factor complex, and membrane raft, and EGFR, DPP4, ADAM17, BCL2, and CDK2 were contained in the cellular components. The verified impact of EGFR on UC by regulating epithelial barrier function could identify the reliability of this GO analysis [35].

**Figure 9:**
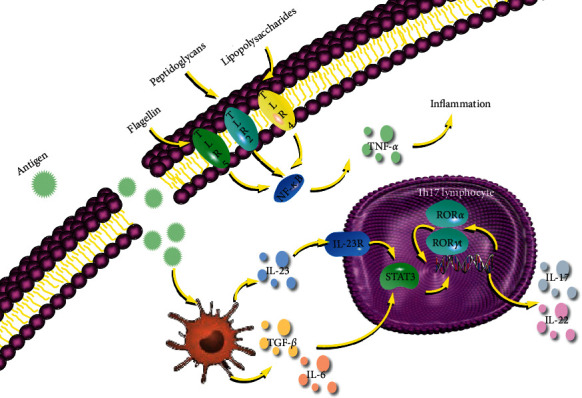
Curcuma played a therapeutic role in UC by regulating inflammatory bowel disease pathway.

The results of pathway analysis and KEGG pathway database [36] analysis showed that the potential mechanisms of curcuma in treating UC were mainly immune regulation, inflammatory reaction, intestinal flora regulation, and the signal pathways related to immunoregulation. Among them, the IBD pathway (Figure 9) was representative and verified in our research.

In the IBD pathway, antigen-presenting cells (APC) endocytosis the invading bacteria and secrete TGF-β and IL-6. Then, TGF-β and IL-6 stimulate Th17 activating STAT3 with RORγt and RORα for further inflammatory cytokines. The activated expression of STAT3 participates in the gene transcription and protein expression of a variety of inflammatory factors such as TNF-*α* and IL-1*β*, thus promoting the formation and persistent aggravation of inflammation [37–39]. Besides, STAT3, another gene symbol, participates in inflammation. TLR4, an important link in the development of the pathogenesis of UC, can nonspecifically bind to pathogen phase molecules, initiate signal transduction, and eventually lead to the release of nuclear factors [40]. However, lipopolysaccharides activate NK-*κ*B pathway through TLR4 in intestinal epithelial cells and then induce inflammation by secreting TNF-*α* in Toll-like receptor signaling pathway. Curcuma may inhibit the inflammation through TLR4, TNF, STAT3, and ROR*γ*t.

## 5. Conclusion

In summary, consistent with clinical experience in the long history, experimental verification, and our HE staining, as well as our WB results, curcuma has significant advantages in the treatment of UC. At the same time, the uncovered targets and pathways were excavated for a better research of how curcuma relieves UC. It has a significant value to provide theoretical basis for clinical treatment of UC and a potent evidence for further study of the mechanism of curcuma in the treatment of UC.

## Figures and Tables

**Table 1:** Basic information for curcuma compound ingredients.

CAS	Molecule name	Structure	OB (%)/GI absorption	Druglikeness
474-62-4^*∗*^	Campesterol	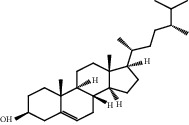	37.58	0.71
57-88-5^*∗*^	CLR	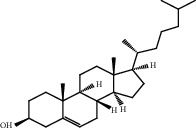	37.87	0.68
83-48-7^*∗*^	Stigmasterol	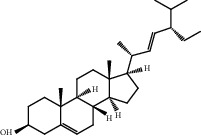	43.87	0.76
76474-56-1^▲^	Dihydrocurcumin	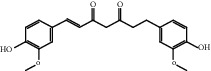	High	0.55
22608-12-4^▲^	Bisdemethoxycurcumin	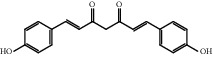	High	0.55
22608-11-3^▲^	Demethoxycurcumin	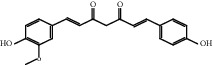	High	0.55
458-37-7^▲^	Curcumin	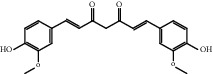	High	0.55
87440-60-6^▲^	Curlone	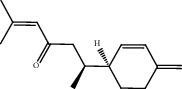	High	0.55
21698-40-8^▲^	Procurcumenol	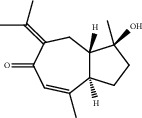	High	0.55
83-46-5^▲^	Beta-sitosterol	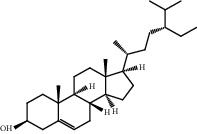	High	0.55
2309-07-1^☆^	Methyl ferulate [22]	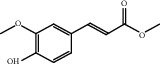	High	0.55
121-33-5^☆^	Vanillin [22]	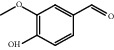	High	0.55
NA^☆^	1,7-Bis(4-hydroxy-3-methoxyphenyl)-1,4,6-heptadiene-4-one [23]	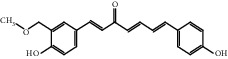	High	0.55

^*∗*^Ingredients searched from TCMSP. ^▲^Ingredients searched from SGST. ^☆^Ingredients searched from CNKI and PubMed.

**Table 2:** Information on potential targets and the topological attributes.

No.	Gene name	Protein name	UniProt ID	Degree
1	AKT1	AKT serine/threonine kinase 1	P31749	43
2	EGFR	Epidermal growth factor receptor	P00533	35
3	TNF	Tumor necrosis factor	P01375	35
4	STAT3	Signal transducer and activator of transcription 3	P40763	33
5	PTGS2	Prostaglandin-endoperoxide synthase 2	P35354	32
6	MMP9	Matrix metallopeptidase 9	P50281	31
7	ESR1	Estrogen receptor 1	P03372	25
8	EP300	E1A-binding protein P300	Q09472	23
9	TLR4	Toll-like receptor 4	O00206	23
10	PPARG	Peroxisome proliferator-activated receptor gamma	P37231	20
11	SERPINE1	Serpin family E member 1	P05121	18
12	CDK4	Cyclin-dependent kinase 4	P11802	14
13	NR3C1	Nuclear receptor subfamily 3 group C member 1	P04150	14
14	CDK1	Cyclin-dependent kinase 1	P06493	13
15	CDK2	Cyclin-dependent kinase 2	P24941	13
16	MMP3	Matrix metallopeptidase 3	P08254	13
17	ADAM17	ADAM metallopeptidase domain 17	P78536	12
18	CHEK1	Checkpoint kinase 1	O14757	12
19	LCK	LCK proto-oncogene, Src family tyrosine kinase	P06239	12
20	MMP13	Matrix metallopeptidase 13	P45452	12
21	CXCR2	C-X-C motif chemokine receptor 2	P25025	11
22	MMP14	Matrix metallopeptidase 14	P50281	11
23	MMP7	Matrix metallopeptidase 7	P09237	11
24	RPS6KB1	Ribosomal protein S6 kinase B1	P23443	11
25	CA9	Carbonic anhydrase 9	Q16790	10
26	NOS2	Nitric oxide synthase 2	P22894	10
27	PTGS1	Prostaglandin-endoperoxide synthase 1	P23219	10
28	TLR9	Toll-like receptor 9	Q9NR96	10
29	ALOX5	Arachidonate 5-lipoxygenase	P09917	9
30	MET	MET proto-oncogene, receptor tyrosine kinase	P08581	9
31	MMP8	Matrix metallopeptidase 8	P22894	9
32	RAF1	Raf-1 proto-oncogene, serine/threonine kinase	P04049	9
33	AURKA	Aurora kinase A	O14965	8
34	BCL2	BCL2 apoptosis regulator	P10415	8
35	ESR2	Estrogen receptor 2	Q92731	8
36	F3	Coagulation factor III, tissue factor	P13726	8
37	MIF	Macrophage migration inhibitory factor	P14174	8
38	NFE2L2	Nuclear factor, erythroid 2 like 2	P09237	8
39	TOP1	DNA topoisomerase I	P11387	8
40	ALOX15	Arachidonate 15-lipoxygenase	P16050	7
41	DPP4	Dipeptidyl peptidase 4	P27487	7
42	PTPN2	Protein tyrosine phosphatase nonreceptor type 2	P17706	7
43	ABCC1	ATP binding cassette subfamily C member 1	P33527	6
44	PPARA	Peroxisome proliferator-activated receptor alpha	Q07869	6
45	VDR	Vitamin D receptor	P11473	6
46	CYP2C19	Cytochrome P450 family 2 subfamily C member 19	P33261	5
47	BRAF	B-Raf proto-oncogene, serine/threonine kinase	P15056	3
48	HTR1A	5-Hydroxytryptamine receptor 1A	P08908	3
49	RORC	RAR-related orphan receptor C	P51449	3
50	SLC6A4	Solute carrier family 6 member 4	P31645	3
51	TYR	Tyrosinase	P14679	3
52	NR1H2	Nuclear receptor subfamily 1 group H member 2	P55055	2
53	PTGER2	Prostaglandin E receptor 2	P43116	2
54	HSD11B1	Hydroxysteroid 11-beta dehydrogenase 1	P28845	2

## Data Availability

The data used to support the findings of this study are available from the corresponding author upon request.

## Supplementary Materials

Supplementary Materials Table S1: compound targets for each component in curcuma by prediction. Table S2: UC-related targets in GeneCards and DisGeNet and papers published in CNKI and PubMed. Table S3: component-target-pathway connection.
