# Supplementary material for: Uncovering the Mechanism of Curcuma in the Treatment of Ulcerative Colitis Based on Network Pharmacology, Molecular Docking Technology, and Experiment Verification
Source: Evid Based Complement Alternat Med. 2021 Jun 16;2021:6629761. doi: 10.1155/2021/6629761 (PMC8225429; doi:10.1155/2021/6629761)
Supplement: Supplementary Materials — Table S1: compound targets for each component in curcuma by prediction. Table S2: UC-related targets in GeneCards and DisGeNet and papers published in CNKI and PubMed. Table S3: component-target-pathway connection. [file 6629761.f1.zip › 6629761.f1/Table S1 components and targets of curcuma.pdf]

HMGCR		
CYP51A1	campester	HMGCR
NR1H3	campester	CYP51A1
NPC1L1	campester	NR1H3
AR	campester	NPC1L1
RORC	campester	AR
SHBG	campester	RORC
CYP17A1	campester	SHBG
SREBF2	campester	CYP17A1
ESR1	campester	SREBF2
ESR2	campester	ESR1
CYP19A1	campester	ESR2
SLC6A2	campester	CYP19A1
RORA	campester	SLC6A2
CYP2C19	campester	RORA
PTPN1	campester	CYP2C19
NR1H2	CLR	NPC1L1
SERPINA6	CLR	NR1H3
SLC6A4	CLR	RORC
ACHE	CLR	SREBF2
BCHE	CLR	HMGCR
NR1I3	CLR	SHBG
CHRM2	CLR	CYP51A1
DHCR7	CLR	CYP17A1
VDR	CLR	CYP19A1
G6PD	CLR	AR
GLRA1	CLR	RORA
PTGER1	CLR	ESR1
PTGER2	CLR	ESR2
TBXAS1	CLR	PTPN1
PTGES	CLR	SLC6A2
PPARA	CLR	NR1H2
PPARD	CLR	SERPINA6
CES2	CLR	SLC6A4
SQLE	CLR	ACHE
NOS2	CLR	BCHE
PTPN6	CLR	NR1I3
PTPN2	CLR	CYP2C19
HSD11B1	CLR	CHRM2
FDFT1	CLR	DHCR7
SIGMAR1	CLR	VDR
PPARG	CLR	G6PD
UGT2B7	CLR	GLRA1
SHH	CLR	PTGER1
POLB	CLR	PTGER2
PREP	CLR	TBXAS1
ALOX5	CLR	PTGES
MAOA	CLR	PPARA
APP	CLR	PPARD
EP300	CLR	CES2
TLR9	CLR	SQLE
BACE1	CLR	NOS2
TOP2A	CLR	PTPN6
HSD17B1	CLR	PTPN2
NFE2L2	CLR	HSD11B1
PTGS1	CLR	FDFT1
EGFR	CLR	SIGMAR1
RAF1	CLR	PPARG

BRAF	CLR	UGT2B7
MET	CLR	SHH
ADAM17	CLR	POLB
LCK	CLR	PREP
DNM1	Stigmaster	NPC1L1
HSD17B3	Stigmaster	NR1H3
CDK2	Stigmaster	RORC
AGTR1	Stigmaster	SHBG
SYK	Stigmaster	HMGCR
AURKB	Stigmaster	SREBF2
RPS6KB1	Stigmaster	CYP19A1
AURKA	Stigmaster	AR
CCNE1	Stigmaster	CYP17A1
HTR1A	Stigmaster	RORA
SERPINE1	Stigmaster	ESR1
CHEK1	Stigmaster	ESR2
PDK1	Stigmaster	CYP51A1
WEE1	Stigmaster	CYP2C19
THRA	Stigmaster	BCHE
THRB	Stigmaster	PTPN1
GCGR	Stigmaster	SERPINA6
ALPL	Stigmaster	G6PD
CELA1	Stigmaster	ACHE
MELK	dihydrocu	ALOX5
HSD17B2	dihydrocu	MAOA
ALOX5AP	dihydrocu	APP
TYR	dihydrocu	EP300
BMP1	dihydrocu	PTGES
HDAC8	dihydrocu	TLR9
ROCK2	dihydrocu	BACE1
ROCK1	dihydrocu	TOP2A
MMP14	dihydrocu	HSD17B1
MMP7	dihydrocu	NFE2L2
MMP8	dihydrocu	PTGS1
PTGS2	dihydrocu	EGFR
NOX4	dihydrocu	RAF1
CFD	dihydrocu	BRAF
TOP1	dihydrocu	MET
CDK1	dihydrocu	ADAM17
MAP3K12	dihydrocu	LCK
NCOR2	dihydrocu	DNM1
HDAC11	dihydrocu	HSD17B3
HDAC10	dihydrocu	CDK2
IMPDH1	dihydrocu	AGTR1
SLC5A1	dihydrocu	SYK
P2RY12	dihydrocu	AURKB
CDK4	dihydrocu	RPS6KB1
DPP4	dihydrocu	AURKA
IKBKG	dihydrocu	CCNE1
DPP7	dihydrocu	HTR1A
ADAM10	dihydrocu	SERPINE1
STAT3	dihydrocu	CHEK1
HSP90AB1	dihydrocu	PDK1
YWHAG	dihydrocu	WEE1
MIF	dihydrocu	THRA
SGK1	dihydrocu	THRB
CXCR2	dihydrocu	GCGR
PDE10A	dihydrocu	ESR1

TNF	dihydrocu	ESR2
CDK5R1	dihydrocu	ALPL
CTSK	dihydrocu	CELA1
PLK1	dihydrocu	PREP
F3	dihydrocu	MELK
GLO1	dihydrocu	HSD17B2
AKT1	dihydrocu	ALOX5AP
CA7	dihydrocu	TYR
CA6	dihydrocu	BMP1
CA12	dihydrocu	HDAC8
CA14	dihydrocu	ROCK2
CA9	dihydrocu	ROCK1
CA5A	dihydrocu	MMP14
GSK3B	dihydrocu	MMP7
ABCC1	dihydrocu	MMP8
CA2	dihydrocu	PTGS2
CA1	dihydrocu	NOX4
MMP13	dihydrocu	CFD
GRIK1	dihydrocu	TOP1
IMPDH2	dihydrocu	CDK1
MMP9	dihydrocu	MAP3K12
MMP3	dihydrocu	NCOR2
BCL2	dihydrocu	HDAC11
NR3C1	dihydrocu	HDAC10
MAOB	dihydrocu	IMPDH1
ERN1	dihydrocu	SLC5A1
ALOX15	dihydrocu	P2RY12
PRKCE	dihydrocu	CDK4
CA4	dihydrocu	DPP4
CA13	dihydrocu	IKBKG
CA5B	dihydrocu	DPP7
TLR4	dihydrocu	ADAM10
	dihydrocu	STAT3
	dihydrocu	HSD11B1
	dihydrocu	HSP90AB1
	dihydrocu	YWHAG
	dihydrocu	MIF
	dihydrocu	SGK1
	dihydrocu	CXCR2
	dihydrocu	PDE10A
	dihydrocu	TNF
	dihydrocu	CDK5R1
	dihydrocu	CTSK
	dihydrocu	PLK1
	bisdemeth	BACE1
	bisdemeth	MAOA
	bisdemeth	APP
	bisdemeth	EP300
	bisdemeth	PTGES
	bisdemeth	TLR9
	bisdemeth	F3
	bisdemeth	HSD11B1
	demethox	BACE1
	demethox	MAOA
	demethox	APP
	demethox	EP300
	demethox	PTGES
	demethox	TLR9

demethoxy	TOP2A
demethoxy	GLO1
demethoxy	ALOX5
demethoxy	NFE2L2
demethoxy	PTGS1
curcumin	MAOA
curcumin	APP
curcumin	EP300
curcumin	PTGES
curcumin	TLR9
curcumin	BACE1
curcumin	TOP2A
curcumin	NFE2L2
curcumin	ALOX5
curcumin	PTGS1
curcumin	GLO1
curcumin	IKBKG
curcumin	EGFR
curcumin	HSD17B3
curcumin	AKT1
curcumin	STAT3
curcumin	HSD11B1
curcumin	CA7
curcumin	CA6
curcumin	CA12
curcumin	CA14
curcumin	CA9
curcumin	CA5A
curcumin	GSK3B
curcumin	ABCC1
curcumin	RAF1
curcumin	BRAF
curcumin	CHEK1
curcumin	WEE1
curcumin	PDK1
curcumin	CA2
curcumin	CA1
curcumin	TOP1
curcumin	GCGR
curcumin	AURKB
curcumin	MMP13
curcumin	ADAM17
curcumin	RPS6KB1
curcumin	AURKA
curcumin	TYR
curcumin	ALOX5AP
curcumin	SERPINE1
curcumin	CDK2
curcumin	MMP14
curcumin	NOX4
curcumin	CFD
curcumin	AGTR1
curcumin	BMP1
curcumin	MELK
curcumin	ALPL
curcumin	GRIK1
curcumin	THRA
curcumin	THRB

curcumin	MMP8
curcumin	ADAM10
curcumin	IMPDH2
curcumin	CXCR2
curcumin	CELA1
curcumin	PREP
curcumin	SGK1
curcumin	DPP4
curcumin	DPP7
curcumin	MMP9
curcumin	IMPDH1
curcumin	MMP3
curcumin	MMP7
curcumin	BCL2
curlone	CYP19A1
procurcum	NR3C1
methyl fer	MAOB
methyl fer	CA2
methyl fer	CA7
methyl fer	CA1
methyl fer	CA12
methyl fer	CA14
methyl fer	CA9
vanillin	ERN1
vanillin	CA2
beta-Sitos	NPC1L1
beta-Sitos	NR1H3
beta-Sitos	RORC
beta-Sitos	HMGCR
beta-Sitos	SHBG
beta-Sitos	CYP51A1
beta-Sitos	CYP17A1
beta-Sitos	CYP19A1
beta-Sitos	SREBF2
beta-Sitos	AR
beta-Sitos	RORA
beta-Sitos	ESR1
beta-Sitos	ESR2
1,7-bis(4-I	NFE2L2
1,7-bis(4-I	EGFR
1,7-bis(4-I	CA2
1,7-bis(4-I	ALOX5
1,7-bis(4-I	PTGS1
1,7-bis(4-I	CA7
1,7-bis(4-I	APP
1,7-bis(4-I	CA1
1,7-bis(4-I	ALOX15
1,7-bis(4-I	CA6
1,7-bis(4-I	MMP9
1,7-bis(4-I	CA12
1,7-bis(4-I	PRKCE
1,7-bis(4-I	CA9
1,7-bis(4-I	CA4
1,7-bis(4-I	EP300
1,7-bis(4-I	CA13
1,7-bis(4-I	CA5B
1,7-bis(4-I	F3
1,7-bis(4-I	HSD11B1

1,7-bis(4-l	NOS2
1,7-bis(4-l	BACE1
1,7-bis(4-l	PTGES
1,7-bis(4-l	TLR4
