# Supplementary material for: Uncovering the Mechanism of Curcuma in the Treatment of Ulcerative Colitis Based on Network Pharmacology, Molecular Docking Technology, and Experiment Verification
Source: Evid Based Complement Alternat Med. 2021 Jun 16;2021:6629761. doi: 10.1155/2021/6629761 (PMC8225429; doi:10.1155/2021/6629761)
Supplement: Supplementary Materials — Table S1: compound targets for each component in curcuma by prediction. Table S2: UC-related targets in GeneCards and DisGeNet and papers published in CNKI and PubMed. Table S3: component-target-pathway connection. [file 6629761.f1.zip › 6629761.f1/Table S2 UC targets (1).pdf]

Gene Syml	Descriptio	Category	Gifts	GC Id	Relevance score		1.795
NOD2	Nucleotide	Protein Co	49	GC16P050	73.59		
IL6	Interleukin	Protein Co	51	GC07P022	69.22		
IL10	Interleukin	Protein Co	49	GC01M20(	65.24		
TLR4	Toll Like R	Protein Co	52	GC09P117	57.87		
IL23R	Interleukin	Protein Co	46	GC01P067	57.73		
HLA-DRB1	Major Hist	Protein Co	47	GC06M03	57.41		
IL1RN	Interleukin	Protein Co	50	GC02P114	51.81		
ABCB1	ATP Bindir	Protein Co	52	GC07M08	50.61		
TNF	Tumor Ne	Protein Co	53	GC06P033	49.98		
CCR6	C-C Motif	Protein Co	46	GC06P167	49.01		
IRF5	Interferon	Protein Co	50	GC07P128	46.02		
ATG16L1	Autophag	Protein Co	45	GC02P233	45.82		
IL10RA	Interleukin	Protein Co	47	GC11P117	44.81		
IRGM	Immunity	Protein Co	38	GC05P150	43.69		
IL10RB	Interleukin	Protein Co	46	GC21P033	43.33		
MLH1	MutL Hom	Protein Co	49	GC03P036	41.39		
INAVA	Innate Imr	Protein Co	29	GC01P200	41.37		
PRTN3	Proteinase	Protein Co	46	GC19P000	40.01		
CTNNB1	Catenin Be	Protein Co	54	GC03P041	39.56		
CD40LG	CD40 Liga	Protein Co	49	GC0XP136	39.15		
CXCL8	C-X-C Mo	Protein Co	43	GC04P073	39.02		
IL1B	Interleukin	Protein Co	50	GC02M11	38.97		
SMAD7	SMAD Fan	Protein Co	44	GC18M04	37.67		
TLR2	Toll Like R	Protein Co	52	GC04P153	37.38		
AKT1	AKT Serine	Protein Co	56	GC14M10	37.07		
MPO	Myeloperc	Protein Co	51	GC17M05	36.99		
TP53	Tumor Pro	Protein Co	55	GC17M00	36.64		
SMAD4	SMAD Fan	Protein Co	52	GC18P051	35.96		
MSH2	MutS Hom	Protein Co	50	GC02P047	35.36		
IL2	Interleukin	Protein Co	47	GC04M12	34.57		
STAT3	Signal Trai	Protein Co	54	GC17M04	34.31		
MSH6	MutS Hom	Protein Co	51	GC02P047	33.37		
HLA-B	Major Hist	Protein Co	46	GC06M03	33		
IFNG	Interferon	Protein Co	50	GC12M06	32.66		
CAT	Catalase	Protein Co	51	GC11P034	32.04		
CRP	C-Reactive	Protein Co	48	GC01M15	31.82		
CTLA4	Cytotoxic	Protein Co	47	GC02P203	31.63		
NLRP3	NLR Famil	Protein Co	48	GC01P247	31.25		
MMP1	Matrix Me	Protein Co	53	GC11M10	31.24		
FAS	Fas Cell Su	Protein Co	52	GC10P088	30.43		
IL4	Interleukin	Protein Co	48	GC05P132	30.06		
ELANE	Elastase, N	Protein Co	48	GC19P000	29.35		
FOXP3	Forkhead I	Protein Co	48	GC0XM04	29.34		
TNFAIP3	TNF Alpha	Protein Co	49	GC06P137	28.76		
TGFB1	Transform	Protein Co	54	GC19M04	28.62		
IL17A	Interleukin	Protein Co	44	GC06P052	28.46		
PTPN22	Protein Ty	Protein Co	48	GC01M11	28.09		
HLA-DQB	Major Hist	Protein Co	45	GC06M03	27.93		
TNFRSF1B	TNF Recep	Protein Co	49	GC01P012	27.71		
ICAM1	Intercellul	Protein Co	52	GC19P010	27.33		
PTGS2	Prostaglan	Protein Co	50	GC01M18	27.31		
GAST	Gastrin	Protein Co	41	GC17P041	27.13		
NGF	Nerve Gro	Protein Co	52	GC01M11	26.98		
JAK2	Janus Kina	Protein Co	55	GC09P004	26.96		
APC	APC Regul	Protein Co	50	GC05P112	26.95		
MST1	Macropha	Protein Co	46	GC03M04	26.84		
HLA-A	Major Hist	Protein Co	48	GC06P033	26.81		

MMP9	Matrix Me	Protein Co	54	GC20P046	26.65
SLC11A1	Solute Car	Protein Co	48	GC02P218	26.52
MUC2	Mucin 2, C	Protein Co	40	GC11P001	26.37
RELA	RELA Proto	Protein Co	51	GC11M065	26.3
MEFV	MEFV Inna	Protein Co	44	GC16M003	26.27
FASLG	Fas Ligand	Protein Co	49	GC01P172	26.2
PPARG	Peroxisom	Protein Co	54	GC03P012	26.05
STAT4	Signal Trar	Protein Co	47	GC02M191	25.21
BTNL2	Butyrophil	Protein Co	39	GC06M032	24.83
S100A8	S100 Calci	Protein Co	44	GC01M153	24.8
TNFSF15	TNF Super	Protein Co	47	GC09M114	24.55
TNFRSF1A	TNF Recep	Protein Co	51	GC12M006	24.54
IL13	Interleukin	Protein Co	46	GC05P132	24.28
VEGFA	Vascular E	Protein Co	51	GC06P043	24.25
ALB	Albumin	Protein Co	51	GC04P073	24.11
IL22	Interleukin	Protein Co	43	GC12M068	23.6
TLR5	Toll Like R	Protein Co	49	GC01M223	23.27
REG4	Regenerat	Protein Co	41	GC01M119	23.15
CARD9	Caspase R	Protein Co	45	GC09M136	23.14
STAT1	Signal Trar	Protein Co	55	GC02M190	23.07
PSTPIP1	Proline-Se	Protein Co	45	GC15P076	22.68
CCND1	Cyclin D1	Protein Co	53	GC11P069	22.64
KRAS	KRAS Proto	Protein Co	52	GC12M025	22.52
CTSG	Cathepsin	Protein Co	46	GC14M024	22.49
NOS2	Nitric Oxic	Protein Co	51	GC17M027	22.25
IL1R1	Interleukin	Protein Co	47	GC02P102	22.22
ITGB2	Integrin Su	Protein Co	52	GC21M044	22.03
FGF2	Fibroblast	Protein Co	49	GC04P122	21.89
REL	REL Proto-	Protein Co	48	GC02P060	21.83
IL23A	Interleukin	Protein Co	41	GC12P056	21.74
TPMT	Thiopurine	Protein Co	49	GC06M018	21.71
XDH	Xanthine D	Protein Co	47	GC02M031	21.71
S100A12	S100 Calci	Protein Co	41	GC01M153	21.62
ERAP1	Endoplasm	Protein Co	45	GC05M096	21.61
MYO9B	Myosin IXB	Protein Co	44	GC19P022	21.46
EGF	Epidermal	Protein Co	52	GC04P109	21.44
LACC1	Laccase Do	Protein Co	35	GC13P043	21.3
CCR3	C-C Motif	Protein Co	48	GC03P046	21.28
PTGS1	Prostaglan	Protein Co	48	GC09P122	21.19
LTF	Lactotrans	Protein Co	45	GC03M046	21.09
MIR21	MicroRNA	RNA Gene	24	GC17P059	21.05
IL12B	Interleukin	Protein Co	45	GC05M159	20.96
SST	Somatosta	Protein Co	43	GC03M187	20.56
BPI	Bactericida	Protein Co	43	GC20P038	20.56
HRAS	HRas Proto	Protein Co	53	GC11M000	20.48
CD4	CD4 Mole	Protein Co	51	GC12P006	20.43
GPR35	G Protein-	Protein Co	44	GC02P240	20.37
TCF4	Transcripti	Protein Co	48	GC18M055	20.27
SMAD3	SMAD Fan	Protein Co	50	GC15P067	20.25
DUOX2	Dual Oxida	Protein Co	44	GC15M045	20.18
CIITA	Class II Ma	Protein Co	46	GC16P010	19.87
IL18	Interleukin	Protein Co	45	GC11M112	19.86
FCGR2A	Fc Fragme	Protein Co	47	GC01P161	19.71
NLRP1	NLR Family	Protein Co	45	GC17M005	19.71
IL17F	Interleukin	Protein Co	43	GC06M052	19.64
PTPRC	Protein Ty	Protein Co	52	GC01P198	19.54
CLDN18	Claudin 18	Protein Co	40	GC03P137	19.49
CCN2	Cellular Co	Protein Co	40	GC06M131	19.39

SPP1	Secreted F	Protein Co	48	GC04P087	19.37
S100A9	S100 Calci	Protein Co	44	GC01P153	19.15
MUC12	Mucin 12,	Protein Co	32	GC07P100	19.1
IL12A	Interleukin	Protein Co	45	GC03P159	18.9
BRAF	B-Raf Prot	Protein Co	55	GC07M14(	18.9
PIK3CA	Phosphati(	Protein Co	54	GC03P179	18.83
C4A	Compleme	Protein Co	44	GC06P033	18.83
IL2RA	Interleukin	Protein Co	52	GC10M00(	18.5
CD28	CD28 Mol(	Protein Co	48	GC02P203	18.36
CLDN2	Claudin 2	Protein Co	43	GC0XP106	18.32
UCN	Urocortin	Protein Co	39	GC02M02	18.25
ICOSLG	Inducible	Protein Co	40	GC21M04	18.11
MIR145	MicroRNA	RNA Gene	22	GC05P149	18.03
CCL2	C-C Motif	Protein Co	50	GC17P034	17.96
CDH1	Cadherin 1	Protein Co	52	GC16P068	17.9
NFKB1	Nuclear Fa	Protein Co	54	GC04P102	17.9
TIMP1	TIMP Meta	Protein Co	47	GC0XP047	17.89
SLC22A4	Solute Car	Protein Co	44	GC05P132	17.85
ERBB2	Erb-B2 Re	Protein Co	56	GC17P039	17.81
CHGA	Chromogr	Protein Co	44	GC14P092	17.76
ATP4A	ATPase H	Protein Co	42	GC19M04	17.66
ZFP90	ZFP90 Zin	Protein Co	36	GC16P068	17.6
HRH2	Histamine	Protein Co	45	GC05P175	17.43
SLC22A5	Solute Car	Protein Co	48	GC05P132	17.41
RET	Ret Proto-	Protein Co	55	GC10P043	17.31
F2	Coagulatic	Protein Co	50	GC11P046	17.3
F5	Coagulatic	Protein Co	46	GC01M16	17.2
ITGAM	Integrin Su	Protein Co	48	GC16P031	17.17
MIR126	MicroRNA	RNA Gene	23	GC09P136	17.16
PLA2G2A	Phospholi	Protein Co	47	GC01M01	17.11
TLR9	Toll Like R	Protein Co	46	GC03M05	17.07
NOD1	Nucleotide	Protein Co	46	GC07M03(	17.05
SELE	Selectin E	Protein Co	45	GC01M16	17.04
ODC1	Ornithine	Protein Co	48	GC02M01(	17.04
HGF	Hepatocyt	Protein Co	53	GC07M08	16.97
PLG	Plasminog	Protein Co	49	GC06P160	16.71
MTHFR	Methylene	Protein Co	48	GC01M01	16.55
HLA-DQA	Major Hist	Protein Co	44	GC06P033	16.5
CDKN1A	Cyclin Dep	Protein Co	51	GC06P046	16.42
DEFB4A	Defensin B	Protein Co	38	GC08P007	16.41
FCGR3B	Fc Fragme	Protein Co	44	GC01M16	16.4
IRF1	Interferon	Protein Co	49	GC05M13	16.25
TFF1	Trefoil Fac	Protein Co	46	GC21M04	16.25
TYK2	Tyrosine K	Protein Co	54	GC19M01(	16.24
TERT	Telomeras	Protein Co	53	GC05M00	16.23
CD8A	CD8a Mol(	Protein Co	48	GC02M08(	16.2
MUC5AC	Mucin 5A(	Protein Co	40	GC11P001	16.16
APOH	Apolipopr	Protein Co	45	GC17M06(	16.11
WRN	WRN Rec(	Protein Co	47	GC08P031	16.03
PTEN	Phosphata	Protein Co	54	GC10P087	15.99
DEFA5	Defensin A	Protein Co	39	GC08M00	15.84
IL1A	Interleukin	Protein Co	45	GC02M11	15.81
MMP2	Matrix Me	Protein Co	55	GC16P055	15.8
ATP12A	ATPase H	Protein Co	44	GC13P024	15.79
MVK	Mevalonat	Protein Co	50	GC12P109	15.76
SERPINC1	Serpin Fan	Protein Co	49	GC01M17	15.6
PTPN2	Protein Ty	Protein Co	48	GC18M01(	15.58
MICA	MHC Class	Protein Co	40	GC06P031	15.53

GNAS	GNAS Con	Protein Co	53	GC20P058	15.52
VDR	Vitamin D	Protein Co	53	GC12M047	15.5
ITGA4	Integrin Su	Protein Co	50	GC02P181	15.43
PLCG2	Phospholip	Protein Co	52	GC16P081	15.43
H19	H19 Imprin	RNA Gene	28	GC11M001	15.39
CALCA	Calcitonin	Protein Co	44	GC11M014	15.35
ITGB4	Integrin Su	Protein Co	50	GC17P075	15.25
RASGRP1	RAS Guany	Protein Co	47	GC15M038	15.24
MMP3	Matrix Me	Protein Co	52	GC11M102	15.22
VCAM1	Vascular C	Protein Co	47	GC01P100	15.16
MIR143	MicroRNA	RNA Gene	23	GC05P149	15.15
EGFR	Epidermal	Protein Co	56	GC07P055	15.1
HP	Haptoglob	Protein Co	45	GC16P072	15.05
SERPINE1	Serpin Fam	Protein Co	52	GC07P101	15
BAX	BCL2 Asso	Protein Co	50	GC19P048	14.88
HLA-C	Major Hist	Protein Co	45	GC06M031	14.86
MIR34A	MicroRNA	RNA Gene	22	GC01M009	14.85
CSF2	Colony Sti	Protein Co	45	GC05P132	14.84
LTA	Lymphoto:	Protein Co	43	GC06P033	14.69
CXCR2	C-X-C Mo	Protein Co	50	GC02P218	14.68
PLA2G4A	Phospholip	Protein Co	50	GC01P186	14.65
ACE	Angiotens	Protein Co	51	GC17P063	14.56
CASR	Calcium Se	Protein Co	52	GC03P122	14.43
NCF4	Neutrophi	Protein Co	50	GC22P036	14.42
GHRL	Ghrelin An	Protein Co	44	GC03M010	14.32
IL5	Interleukin	Protein Co	46	GC05M132	14.31
INS	Insulin	Protein Co	50	GC11M002	14.3
DNMT1	DNA Meth	Protein Co	52	GC19M010	14.29
GRP	Gastrin Re	Protein Co	42	GC18P059	14.23
PTGER4	Prostaglan	Protein Co	48	GC05P040	14.19
TFF3	Trefoil Fac	Protein Co	43	GC21M042	14.15
CCR5	C-C Motif	Protein Co	48	GC03P046	14.15
MIF	Macropha	Protein Co	51	GC22P023	14.13
RIPK1	Receptor I	Protein Co	50	GC06P003	14.13
LAMC2	Laminin Su	Protein Co	47	GC01P183	14.11
CDKN2A	Cyclin Dep	Protein Co	52	GC09M021	14.1
MAP3K7	Mitogen-A	Protein Co	52	GC06M090	14.1
DLG5	Discs Larg	Protein Co	38	GC10M077	14.05
HLA-DPA1	Major Hist	Protein Co	42	GC06M033	14.03
CCL5	C-C Motif	Protein Co	45	GC17M035	13.98
MIR150	MicroRNA	RNA Gene	22	GC19M049	13.91
CDKN1B	Cyclin Dep	Protein Co	50	GC12P012	13.88
IFNGR1	Interferon	Protein Co	51	GC06M137	13.87
HMOX1	Heme Oxy	Protein Co	54	GC22P035	13.8
MASP2	Mannan B	Protein Co	45	GC01M011	13.77
PDGFB	Platelet De	Protein Co	51	GC22M044	13.7
CD79A	CD79a Mo	Protein Co	48	GC19P041	13.67
CCL11	C-C Motif	Protein Co	45	GC17P034	13.61
IFNG-AS1	IFNG Antis	RNA Gene	18	GC12P067	13.57
CARMIL2	Capping P	Protein Co	31	GC16P067	13.52
PRKCQ	Protein Kir	Protein Co	51	GC10M006	13.5
CD40	CD40 Mole	Protein Co	50	GC20P046	13.47
MIR141	MicroRNA	RNA Gene	22	GC12P007	13.44
CXCL1	C-X-C Mo	Protein Co	44	GC04P073	13.43
CLEC7A	C-Type Le	Protein Co	45	GC12M010	13.38
ZAP70	Zeta Chair	Protein Co	53	GC02P097	13.37
LYST	Lysosomal	Protein Co	39	GC01M235	13.34
IL33	Interleukin	Protein Co	41	GC09P006	13.29

RTEL1	Regulator	Protein Co	41	GC20P063	13.28
FGF7	Fibroblast	Protein Co	43	GC15P049	13.22
NAT2	N-Acetyltr	Protein Co	44	GC08P018	13.21
MMP7	Matrix Me	Protein Co	50	GC11M10	13.19
CDKN2B	Cyclin Dep	Protein Co	48	GC09M02	13.17
TLR3	Toll Like R	Protein Co	54	GC04P186	13.12
LRBA	LPS Respo	Protein Co	42	GC04M15	13.12
NFE2L2	Nuclear Fa	Protein Co	49	GC02M17	13.09
IGF1	Insulin Like	Protein Co	50	GC12M10	13.01
CYP2C19	Cytochrom	Protein Co	48	GC10P094	12.99
KRT7	Keratin 7	Protein Co	43	GC12P052	12.97
AXIN2	Axin 2	Protein Co	49	GC17M06	12.97
TAC1	Tachykinin	Protein Co	44	GC07P097	12.97
ITGAL	Integrin Su	Protein Co	47	GC16P030	12.94
IL15	Interleukin	Protein Co	42	GC04P141	12.94
HIF1A	Hypoxia In	Protein Co	49	GC14P061	12.93
EPX	Eosinophil	Protein Co	43	GC17P058	12.92
IL7R	Interleukin	Protein Co	48	GC05P035	12.88
MLH3	MutL Hom	Protein Co	43	GC14M07	12.86
CD80	CD80 Mol	Protein Co	43	GC03M11	12.85
IL26	Interleukin	Protein Co	35	GC12M06	12.82
MICB	MHC Class	Protein Co	43	GC06P033	12.78
TAP2	Transporte	Protein Co	45	GC06M03	12.76
POLE	DNA Polyr	Protein Co	50	GC12M13	12.75
POLD1	DNA Polyr	Protein Co	47	GC19P050	12.75
GALNT12	Polypeptic	Protein Co	43	GC09P098	12.75
MT-CO1	Mitochond	Protein Co	35	GCMTP00	12.75
CCL3	C-C Motif	Protein Co	41	GC17M03	12.72
SELP	Selectin P	Protein Co	47	GC01M16	12.68
VIP	Vasoactive	Protein Co	46	GC06P152	12.65
CSF3	Colony Sti	Protein Co	41	GC17P040	12.64
TFF2	Trefoil Fac	Protein Co	43	GC21M04	12.62
DEFB1	Defensin B	Protein Co	41	GC08M00	12.62
RNASE3	Ribonucle	Protein Co	41	GC14P020	12.62
MUC6	Mucin 6, C	Protein Co	39	GC11M00	12.58
FOXD2-AS	FOXD2 Ad	RNA Gene	16	GC01M04	12.58
TYMS	Thymidyla	Protein Co	50	GC18P000	12.52
LEP	Leptin	Protein Co	49	GC07P128	12.52
CXCL5	C-X-C Mo	Protein Co	42	GC04M07	12.51
IBD5	Inflammat	Genetic Lo	5	GC05U990	12.47
TTC7A	Tetratricop	Protein Co	39	GC02P046	12.46
HLA-DRA	Major Hist	Protein Co	48	GC06P032	12.46
IL6ST	Interleukin	Protein Co	47	GC05M05	12.46
IL12RB2	Interleukin	Protein Co	44	GC01P067	12.4
MET	MET Proto	Protein Co	56	GC07P116	12.37
IBD3	Inflammat	Genetic Lo	4	GC06U990	12.36
CASP10	Caspase 1	Protein Co	50	GC02P201	12.35
PYY	Peptide Y	Protein Co	43	GC17M04	12.33
IRAK1	Interleukin	Protein Co	51	GC0XM15	12.3
SOCS3	Suppresso	Protein Co	45	GC17M07	12.3
SOCS1	Suppresso	Protein Co	45	GC16M01	12.28
MIR200C	MicroRNA	RNA Gene	21	GC12P007	12.26
FGFR2	Fibroblast	Protein Co	55	GC10M12	12.23
PDGFRL	Platelet De	Protein Co	43	GC08P017	12.21
IL21	Interleukin	Protein Co	44	GC04M12	12.18
MIR196B	MicroRNA	RNA Gene	20	GC07M02	12.17
MADCAM	Mucosal V	Protein Co	39	GC19P000	12.16
MIR221	MicroRNA	RNA Gene	21	GC0XM04	12.16

PDGFRB	Platelet De	Protein Co	56	GC05M150	12.16
XIAP	X-Linked I	Protein Co	51	GC0XP123	12.15
HSPA1L	Heat Shoc	Protein Co	44	GC06M031	12.05
FN1	Fibronectir	Protein Co	51	GC02M215	12.05
PDCD1	Programm	Protein Co	49	GC02M241	12.05
IBD2	Inflammat	Genetic Lo	6	GC12U990	12.04
CLDN4	Claudin 4	Protein Co	43	GC07P073	12.03
IFNA2	Interferon	Protein Co	42	GC09M021	12.02
SELL	Selectin L	Protein Co	44	GC01M169	11.99
H2AC18	H2A Cluste	Protein Co	27	GC01M149	11.98
DOCK8	Dedicator	Protein Co	44	GC09P000	11.98
MIR25	MicroRNA	RNA Gene	20	GC07M100	11.94
CD55	CD55 Mol	Protein Co	48	GC01P207	11.93
SCT	Secretin	Protein Co	36	GC11M000	11.89
MLN	Motilin	Protein Co	36	GC06M033	11.86
B2M	Beta-2-Mi	Protein Co	50	GC15P044	11.85
IL1RAPL2	Interleukin	Protein Co	38	GC0XP104	11.85
HSPD1	Heat Shoc	Protein Co	49	GC02M197	11.81
RIPK2	Receptor I	Protein Co	48	GC08P089	11.79
DPYD	Dihydropy	Protein Co	54	GC01M097	11.77
FCGR3A	Fc Fragme	Protein Co	46	GC01M161	11.71
CCK	Cholecystc	Protein Co	42	GC03M042	11.67
MYC	MYC Protc	Protein Co	53	GC08P127	11.67
MUTYH	MutY DNA	Protein Co	47	GC01M045	11.66
FOS	Fos Proto-	Protein Co	52	GC14P075	11.65
TACR1	Tachykinin	Protein Co	47	GC02M075	11.65
ETS1	ETS Proto-	Protein Co	51	GC11M128	11.57
ADIPOQ	Adiponect	Protein Co	47	GC03P186	11.55
MIR155	MicroRNA	RNA Gene	19	GC21P025	11.54
MIR140	MicroRNA	RNA Gene	22	GC16P069	11.51
TJP1	Tight Junc	Protein Co	45	GC15M029	11.48
CAV1	Caveolin 1	Protein Co	50	GC07P116	11.48
TGFA	Transform	Protein Co	48	GC02M070	11.47
RFX5	Regulatory	Protein Co	43	GC01M151	11.47
COL7A1	Collagen T	Protein Co	44	GC03M048	11.46
AURKA	Aurora Kin	Protein Co	51	GC20M056	11.46
COL17A1	Collagen T	Protein Co	45	GC10M104	11.45
DCLRE1C	DNA Cross	Protein Co	44	GC10M014	11.44
NKX2-3	NK2 Home	Protein Co	38	GC10P099	11.41
CDX2	Caudal Typ	Protein Co	44	GC13M027	11.41
FERMT1	Fermitin Fa	Protein Co	42	GC20M006	11.4
TREM1	Triggering	Protein Co	43	GC06M041	11.36
PLAU	Plasminog	Protein Co	53	GC10P073	11.32
ALOX5	Arachidon	Protein Co	50	GC10P045	11.29
HMGB1	High Mobi	Protein Co	45	GC13M030	11.29
FUT2	Fucosyltra	Protein Co	45	GC19P048	11.28
CXCL10	C-X-C Mo	Protein Co	45	GC04M076	11.28
MIR222	MicroRNA	RNA Gene	21	GC0XM045	11.26
MUC1	Mucin 1, C	Protein Co	49	GC01M155	11.24
MUC3A	Mucin 3A,	Protein Co	36	GC07P100	11.2
LGALS3	Galectin 3	Protein Co	46	GC14P055	11.18
SRC	SRC Proto	Protein Co	53	GC20P037	11.11
CD14	CD14 Mol	Protein Co	46	GC05M140	11.09
MIRLET7A	MicroRNA	RNA Gene	22	GC09P094	11.05
MIR192	MicroRNA	RNA Gene	22	GC11M064	11.05
MIR20A	MicroRNA	RNA Gene	19	GC13P091	11.05
IL18RAP	Interleukin	Protein Co	40	GC02P102	11.04
SKIV2L	Ski2 Like R	Protein Co	44	GC06P031	11.02

IBD8	Inflammat	Genetic Lc	4	GC16U990	10.99
JUN	Jun Proto-	Protein Co	51	GC01M058	10.98
MAP2K2	Mitogen-A	Protein Co	55	GC19M004	10.97
HPS1	HPS1 Biog	Protein Co	43	GC10M098	10.97
F3	Coagulatic	Protein Co	47	GC01M094	10.92
KLF6	Kruppel Lil	Protein Co	45	GC10M003	10.91
PECAM1	Platelet Ar	Protein Co	41	GC17M064	10.9
NLRP12	NLR Family	Protein Co	44	GC19M053	10.87
CXCR3	C-X-C Mo	Protein Co	45	GC0XM071	10.86
MBL2	Mannose I	Protein Co	48	GC10M052	10.83
ENG	Endoglin	Protein Co	48	GC09M127	10.83
FGFR3	Fibroblast	Protein Co	56	GC04P001	10.82
GZMB	Granzyme	Protein Co	47	GC14M024	10.82
IBD21	Inflammat	Genetic Lc	2	GC18U900	10.8
ENO1	Enolase 1	Protein Co	48	GC01M008	10.8
C1R	Compleme	Protein Co	48	GC12M007	10.76
IL37	Interleukin	Protein Co	39	GC02P114	10.75
IL27	Interleukin	Protein Co	40	GC16M028	10.71
KRT20	Keratin 20	Protein Co	43	GC17M040	10.7
IL3	Interleukin	Protein Co	45	GC05P132	10.7
IL6R	Interleukin	Protein Co	50	GC01P154	10.68
MMP13	Matrix Me	Protein Co	51	GC11M102	10.68
CCR1	C-C Motif	Protein Co	47	GC03M046	10.65
IBD7	Inflammat	Genetic Lc	4	GC01U990	10.61
JAK3	Janus Kina	Protein Co	53	GC19M017	10.61
MIR127	MicroRNA	RNA Gene	21	GC14P104	10.55
IL19	Interleukin	Protein Co	41	GC01P206	10.54
CP	Ceruloplas	Protein Co	49	GC03M149	10.46
MKI67	Marker Of	Protein Co	44	GC10M128	10.46
MIR27A	MicroRNA	RNA Gene	22	GC19M013	10.45
SLC6A4	Solute Car	Protein Co	49	GC17M030	10.45
MAPK14	Mitogen-A	Protein Co	53	GC06P046	10.43
IFNA1	Interferon	Protein Co	40	GC09P021	10.42
CXCL2	C-X-C Mo	Protein Co	42	GC04M074	10.39
NR1I2	Nuclear Re	Protein Co	46	GC03P119	10.39
GSTP1	Glutathion	Protein Co	51	GC11P067	10.37
TLR1	Toll Like R	Protein Co	49	GC04M038	10.36
BGLAP	Bone Gam	Protein Co	41	GC01P156	10.36
NFKBIA	NFKB Inhib	Protein Co	51	GC14M035	10.34
HPGD	15-Hydrox	Protein Co	50	GC04M174	10.29
CCL20	C-C Motif	Protein Co	45	GC02P227	10.29
LCN2	Lipocalin 2	Protein Co	45	GC09P128	10.29
TAPBP	TAP Bindir	Protein Co	44	GC06M033	10.27
IL11	Interleukin	Protein Co	43	GC19M055	10.21
IBD6	Inflammat	Genetic Lc	4	GC19U990	10.2
HNF4A	Hepatocyt	Protein Co	52	GC20P044	10.18
CXCL12	C-X-C Mo	Protein Co	47	GC10M044	10.17
C4B	Compleme	Protein Co	42	GC06P032	10.11
MIR93	MicroRNA	RNA Gene	21	GC07M100	10.08
MIR15B	MicroRNA	RNA Gene	19	GC03P160	10.08
CASP1	Caspase 1	Protein Co	51	GC11M105	10.07
UBAC2	UBA Doma	Protein Co	38	GC13P099	10.05
GCG	Glucagon	Protein Co	42	GC02M162	10.04
SLC9A3	Solute Car	Protein Co	48	GC05M000	10.04
CFB	Compleme	Protein Co	47	GC06P031	10.02
GSTM1	Glutathion	Protein Co	43	GC01P109	10.01
TLR6	Toll Like R	Protein Co	45	GC04M038	10.01
RFXANK	Regulatory	Protein Co	43	GC19P019	10

RFXAP	Regulatory	Protein Co	37	GC13P036	10
PSC	Cholangiti	Genetic Lc	3	GC03U901	9.99
F2RL1	F2R Like Tı	Protein Co	47	GC05P076	9.98
LGALS1	Galectin 1	Protein Co	45	GC22P037	9.97
TMSB4X	Thymosin	Protein Co	41	GC0XP012	9.89
AREG	Amphiregu	Protein Co	45	GC04P074	9.88
NR3C1	Nuclear Re	Protein Co	51	GC05M143	9.88
CEACAM6	CEA Cell A	Protein Co	41	GC19P041	9.83
MGMT	O-6-Meth	Protein Co	50	GC10P129	9.8
EPO	Erythropoi	Protein Co	43	GC07P100	9.79
MIR483	MicroRNA	RNA Gene	19	GC11M002	9.73
NRAS	NRAS Prot	Protein Co	51	GC01M114	9.72
CXCL9	C-X-C Mo	Protein Co	40	GC04M076	9.66
HSPA4	Heat Shoc	Protein Co	43	GC05P133	9.65
IDH1	Isocitrate I	Protein Co	54	GC02M208	9.65
MIR106B	MicroRNA	RNA Gene	22	GC07M100	9.64
POMC	Proopiome	Protein Co	50	GC02M025	9.62
NTRK1	Neurotrop	Protein Co	50	GC01P156	9.6
IL17RA	Interleukin	Protein Co	46	GC22P017	9.6
IBD4	Inflammat	Genetic Lc	4	GC14U990	9.6
IBD9	Inflammat	Genetic Lc	4	GC03U900	9.6
IL2RB	Interleukin	Protein Co	50	GC22M037	9.59
ARID1A	AT-Rich In	Protein Co	46	GC01P026	9.58
ADAM17	ADAM Me	Protein Co	52	GC02M009	9.57
BCL2	BCL2 Apop	Protein Co	53	GC18M063	9.57
ORMDL3	ORMDL Sp	Protein Co	40	GC17M039	9.56
LIFR	LIF Recept	Protein Co	48	GC05M038	9.56
ESR1	Estrogen F	Protein Co	55	GC06P151	9.55
PMS2	PMS1 Hon	Protein Co	50	GC07M005	9.52
GPT	Glutamic--	Protein Co	42	GC08P144	9.51
CASP3	Caspase 3	Protein Co	52	GC04M184	9.5
TGM2	Transgluta	Protein Co	50	GC20M038	9.5
CXCR4	C-X-C Mo	Protein Co	54	GC02M136	9.46
IL1RL1	Interleukin	Protein Co	43	GC02P102	9.46
RORC	RAR Relate	Protein Co	47	GC01M151	9.44
SH2D1A	SH2 Doma	Protein Co	47	GC0XP124	9.4
CD44	CD44 Mol	Protein Co	49	GC11P035	9.38
GREM1	Gremlin 1,	Protein Co	45	GC15P032	9.36
IBD15	Inflammat	Genetic Lc	3	GC10U901	9.36
IBD18	Inflammat	Genetic Lc	3	GC05U901	9.36
IBD11	Inflammat	Genetic Lc	2	GC07U903	9.36
IBD12	Inflammat	Genetic Lc	2	GC03U901	9.36
IBD16	Inflammat	Genetic Lc	2	GC09U901	9.36
IBD20	Inflammat	Genetic Lc	2	GC10U901	9.36
IBD22	Inflammat	Genetic Lc	2	GC17U901	9.36
IBD23	Inflammat	Genetic Lc	2	GC01U902	9.36
IBD24	Inflammat	Genetic Lc	2	GC20U900	9.36
IBD26	Inflammat	Genetic Lc	2	GC12U901	9.36
IBD27	Inflammat	Genetic Lc	2	GC13U900	9.36
DPP4	Dipeptidyl	Protein Co	51	GC02M161	9.33
DMBT1	Deleted In	Protein Co	41	GC10P122	9.33
NOTCH1	Notch Rec	Protein Co	52	GC09M136	9.33
BCL2L1	BCL2 Like	Protein Co	49	GC20M031	9.32
STAT6	Signal Trai	Protein Co	51	GC12M057	9.31
CYP2C9	Cytochron	Protein Co	50	GC10P094	9.25
THBD	Thrombon	Protein Co	45	GC20M023	9.24
TNFRSF25	TNF Recep	Protein Co	45	GC01M006	9.2
MIR146A	MicroRNA	RNA Gene	24	GC05P160	9.2

CHUK	Componer	Protein Co	52	GC10M10(	9.19
SMAD2	SMAD Fan	Protein Co	48	GC18M04	9.19
CYP3A4	Cytochron	Protein Co	51	GC07M09	9.19
NTS	Neurotens	Protein Co	41	GC12P085	9.18
ALOX12	Arachidon	Protein Co	45	GC17P006	9.16
NR1H4	Nuclear Re	Protein Co	50	GC12P100	9.1
PTPN11	Protein Ty	Protein Co	55	GC12P112	9.1
MIR214	MicroRNA	RNA Gene	20	GC01M17	9.06
CYP2D6	Cytochron	Protein Co	50	GC22M04	9.05
PROS1	Protein S	Protein Co	48	GC03M09	9.04
BRCA2	BRCA2 DN	Protein Co	50	GC13P032	9.02
CFH	Compleme	Protein Co	47	GC01P196	9.02
CCL7	C-C Motif	Protein Co	43	GC17P034	9.02
SOD1	Superoxid	Protein Co	54	GC21P031	9
MAPK8	Mitogen-A	Protein Co	51	GC10P048	8.99
MYD88	MYD88 Inn	Protein Co	51	GC03P038	8.96
HSPA2	Heat Shoc	Protein Co	45	GC14P064	8.95
NOX1	NADPH O	Protein Co	43	GC0XM10(	8.9
CCR9	C-C Motif	Protein Co	42	GC03P045	8.89
KRT19	Keratin 19	Protein Co	47	GC17M04	8.83
KIF21B	Kinesin Fa	Protein Co	37	GC01M20(	8.82
KDR	Kinase Inse	Protein Co	55	GC04M05	8.81
HTR3A	5-Hydroxy	Protein Co	47	GC11P113	8.81
TIMP2	TIMP Meta	Protein Co	45	GC17M07	8.81
FGFR1OP	FGFR1 Onc	Protein Co	39	GC06P166	8.79
SIAE	Sialic Acid	Protein Co	38	GC11M12	8.79
CCL4	C-C Motif	Protein Co	42	GC17P036	8.79
ITGAE	Integrin Su	Protein Co	41	GC17M00	8.76
XRCC1	X-Ray Rep	Protein Co	45	GC19M04	8.74
EPCAM	Epithelial (	Protein Co	48	GC02P047	8.7
MUC4	Mucin 4, C	Protein Co	40	GC03M19	8.69
HPRT1	Hypoxanth	Protein Co	49	GC0XP134	8.68
AHR	Aryl Hydrc	Protein Co	50	GC07P016	8.67
TLR8	Toll Like R	Protein Co	49	GC0XP012	8.65
GUCY2C	Guanylate	Protein Co	47	GC12M01	8.63
IL18R1	Interleukin	Protein Co	44	GC02P102	8.62
IGF1R	Insulin Like	Protein Co	56	GC15P098	8.6
DCC	DCC Netri	Protein Co	47	GC18P052	8.6
GUSB	Glucuronic	Protein Co	48	GC07M06	8.59
ABCG2	ATP Bindir	Protein Co	52	GC04M08	8.58
BCL10	BCL10 Imr	Protein Co	47	GC01M08	8.58
MIR203A	MicroRNA	RNA Gene	20	GC14P104	8.56
BMP6	Bone Mor	Protein Co	45	GC06P007	8.55
IL9	Interleukin	Protein Co	45	GC05M13	8.55
EBI3	Epstein-Ba	Protein Co	41	GC19P004	8.55
TNFRSF4	TNF Recep	Protein Co	45	GC01M00	8.52
PLAUR	Plasminog	Protein Co	46	GC19M04	8.51
LGR5	Leucine Ri	Protein Co	44	GC12P071	8.49
PRKCD	Protein Kir	Protein Co	55	GC03P053	8.48
TLR7	Toll Like R	Protein Co	48	GC0XP012	8.48
MIR193A	MicroRNA	RNA Gene	18	GC17P031	8.47
RAC1	Rac Family	Protein Co	51	GC07P006	8.47
VWF	Von Willet	Protein Co	50	GC12M00	8.47
HDC	Histidine D	Protein Co	46	GC15M05(	8.45
MIR31	MicroRNA	RNA Gene	20	GC09M02	8.43
ICAM3	Intercellula	Protein Co	43	GC19M01(	8.43
NR1H2	Nuclear Re	Protein Co	49	GC19P050	8.43
NDUFA13	NADH:Ubi	Protein Co	45	GC19P019	8.43

MAPK3	Mitogen-A	Protein Co	51	GC16M030	8.43
PTH	Parathyroi	Protein Co	48	GC11M013	8.42
TMEFF2	Transmem	Protein Co	40	GC02M191	8.4
MIR200B	MicroRNA	RNA Gene	21	GC01P001	8.4
TNFRSF10A	TNF Recep	Protein Co	47	GC08M023	8.39
TNFRSF10I	TNF Recep	Protein Co	52	GC08M023	8.39
BRIP1	BRCA1 Int	Protein Co	49	GC17M061	8.39
CFHR1	Compleme	Protein Co	41	GC01P196	8.39
MIR34C	MicroRNA	RNA Gene	21	GC11P111	8.39
MIR215	MicroRNA	RNA Gene	19	GC01M220	8.39
MIR451A	MicroRNA	RNA Gene	17	GC17M028	8.39
IBD25	Inflammat	Genetic Lc	2	GC00U932	8.39
ACKR2	Atypical Cl	Protein Co	39	GC03P042	8.38
NCF1	Neutrophi	Protein Co	49	GC07P074	8.35
LOC11080	Solute Car	Biological	2	GC17U902	8.27
ITLN1	Intelectin 1	Protein Co	40	GC01M160	8.26
PIGR	Polymeric	Protein Co	43	GC01M206	8.25
CTSD	Cathepsin	Protein Co	54	GC11M001	8.13
IDO1	Indoleami	Protein Co	47	GC08P039	8.13
ICAM2	Intercellula	Protein Co	47	GC17M064	8.12
KLRC4	Killer Cell L	Protein Co	37	GC12M013	8.12
BIRC5	Baculovira	Protein Co	48	GC17P078	8.11
OCLN	Occludin	Protein Co	46	GC05P069	8.09
GATA3	GATA Binc	Protein Co	50	GC10P008	8.08
ANXA5	Annexin A	Protein Co	48	GC04M121	8.08
IGSF6	Immunogl	Protein Co	36	GC16M021	8.07
TOR1A	Torsin Fam	Protein Co	45	GC09M129	8.06
CDH3	Cadherin 3	Protein Co	49	GC16P068	8.05
CHI3L1	Chitinase 3	Protein Co	45	GC01M203	8.02
GAPDH	Glyceralde	Protein Co	50	GC12P006	8.02
KRT18	Keratin 18	Protein Co	50	GC12P052	7.99
CFTR	CF Transm	Protein Co	52	GC07P117	7.99
CD46	CD46 Mol	Protein Co	48	GC01P207	7.99
F2R	Coagulatic	Protein Co	48	GC05P076	7.98
SCG5	Secretogra	Protein Co	39	GC15P032	7.97
MIR195	MicroRNA	RNA Gene	20	GC17M007	7.96
IL18BP	Interleukin	Protein Co	40	GC11P071	7.95
ITGB1	Integrin Su	Protein Co	51	GC10M032	7.95
CYCS	Cytochron	Protein Co	50	GC07M025	7.93
HLA-G	Major Hist	Protein Co	46	GC06P033	7.92
ENTPD1	Ectonucleo	Protein Co	48	GC10P095	7.9
H2AX	H2A.X Var	Protein Co	37	GC11M119	7.9
CYP1A1	Cytochron	Protein Co	48	GC15M074	7.88
CX3CR1	C-X3-C M	Protein Co	46	GC03M039	7.87
CASP8	Caspase 8	Protein Co	54	GC02P201	7.85
MMP14	Matrix Me	Protein Co	52	GC14P025	7.83
IL16	Interleukin	Protein Co	44	GC15P081	7.8
AMACR	Alpha-Me	Protein Co	47	GC05M033	7.78
SP140	SP140 Nuc	Protein Co	37	GC02P230	7.76
TBX21	T-Box Trar	Protein Co	47	GC17P047	7.76
BIRC3	Baculovira	Protein Co	48	GC11P102	7.76
RUNX3	RUNX Fam	Protein Co	44	GC01M024	7.76
AP3B1	Adaptor R	Protein Co	45	GC05M078	7.75
ABCC2	ATP Bindir	Protein Co	49	GC10P099	7.74
PARP1	Poly(ADP-	Protein Co	51	GC01M226	7.74
AIRE	Autoimmu	Protein Co	46	GC21P044	7.73
PDE4A	Phosphod	Protein Co	45	GC19P010	7.72
GPX2	Glutathion	Protein Co	45	GC14M064	7.72

SRSF6	Serine Anc	Protein Co	40	GC20P043	7.7
EP300	E1A Bindir	Protein Co	52	GC22P041	7.7
MIR26A1	MicroRNA	RNA Gene	21	GC03P037	7.7
CYP1A2	Cytochron	Protein Co	47	GC15P074	7.68
SERPINA4	Serpin Fan	Protein Co	42	GC14P094	7.66
IGF2	Insulin Like	Protein Co	49	GC11M00	7.65
CAMP	Cathelicidi	Protein Co	43	GC03P048	7.64
TEK	TEK Recep	Protein Co	52	GC09P027	7.64
BUB1	BUB1 Mitc	Protein Co	51	GC02M11	7.64
BUB1B	BUB1 Mitc	Protein Co	51	GC15P040	7.64
PTPRJ	Protein Ty	Protein Co	47	GC11P048	7.64
DLC1	DLC1 Rho	Protein Co	46	GC08M01	7.64
PTPN12	Protein Ty	Protein Co	46	GC07P077	7.64
RAD54B	RAD54 Ho	Protein Co	43	GC08M09	7.64
FLCN	Folliculin	Protein Co	42	GC17M01	7.64
MCC	MCC Regu	Protein Co	41	GC05M11	7.64
MIR34B	MicroRNA	RNA Gene	22	GC11P111	7.64
MIR100	MicroRNA	RNA Gene	21	GC11M12	7.64
MIR342	MicroRNA	RNA Gene	20	GC14P100	7.64
CRCS11	Colorectal	Genetic Lc	3	GC20U90	7.64
CRCS2	Colorectal	Genetic Lc	3	GC08U901	7.64
CRCS5	Colorectal	Genetic Lc	3	GC10U901	7.64
CRCS6	Colorectal	Genetic Lc	3	GC08U901	7.64
CRCS9	Colorectal	Genetic Lc	3	GC16U901	7.64
CRCS7	Colorectal	Genetic Lc	2	GC11U901	7.64
CRCS8	Colorectal	Genetic Lc	2	GC14U90	7.64
CCL25	C-C Motif	Protein Co	40	GC19P008	7.64
MIR424	MicroRNA	RNA Gene	17	GC0XM13	7.63
MAPK1	Mitogen-/	Protein Co	52	GC22M02	7.63
TCF7L2	Transcripti	Protein Co	47	GC10P112	7.63
SLC15A1	Solute Car	Protein Co	45	GC13M09	7.61
PSMB9	Proteasom	Protein Co	47	GC06P033	7.61
CD163	CD163 Mc	Protein Co	44	GC12M00	7.59
MMP10	Matrix Me	Protein Co	48	GC11M10	7.54
HDAC9	Histone De	Protein Co	48	GC07P018	7.54
NT5E	5'-Nucleot	Protein Co	52	GC06P085	7.54
BDNF	Brain Deriv	Protein Co	48	GC11M02	7.51
FOSL1	FOS Like 1	Protein Co	45	GC11M06	7.51
MIR24-1	MicroRNA	RNA Gene	20	GC09P095	7.49
HSD11B1	Hydroxyst	Protein Co	51	GC01P209	7.48
RHOA	Ras Homo	Protein Co	48	GC03M04	7.48
RB1	RB Transcr	Protein Co	51	GC13P048	7.48
PSMB8	Proteasom	Protein Co	51	GC06M03	7.48
PCNA	Proliferatir	Protein Co	52	GC20M00	7.47
LCK	LCK Proto	Protein Co	54	GC01P032	7.46
MIR29A	MicroRNA	RNA Gene	22	GC07M13	7.46
TNFRSF18	TNF Recep	Protein Co	44	GC01M00	7.44
MIR223	MicroRNA	RNA Gene	22	GC0XP066	7.42
CLDN1	Claudin 1	Protein Co	49	GC03M19	7.38
PRSS1	Serine Pro	Protein Co	47	GC07P144	7.38
CARD8	Caspase R	Protein Co	40	GC19M04	7.37
CD24	CD24 Mol	Protein Co	32	GC06M10	7.36
TTC37	Tetratricop	Protein Co	40	GC05M09	7.36
MIR142	MicroRNA	RNA Gene	20	GC17M05	7.35
HSP90AA1	Heat Shoc	Protein Co	50	GC14M10	7.35
SRP54	Signal Rec	Protein Co	43	GC14P034	7.35
ANXA1	Annexin A	Protein Co	50	GC09P073	7.34
CEACAM5	CEA Cell A	Protein Co	43	GC19P041	7.31

TNFRSF9	TNF Recep	Protein Co	45	GC01M007	7.29
VIM	Vimentin	Protein Co	51	GC10P017	7.27
CASP9	Caspase 9	Protein Co	49	GC01M015	7.27
MUC3B	Mucin 3B,	Protein Co	18	GC07U903	7.27
ENSG00000278769		RNA Gene	4	GC15P042	7.27
MAP2K1	Mitogen-A	Protein Co	55	GC15P066	7.26
MCL1	MCL1 Apo	Protein Co	49	GC01M150	7.26
CCL26	C-C Motif	Protein Co	39	GC07M075	7.25
TNFSF10	TNF Super	Protein Co	48	GC03M172	7.24
RNF114	Ring Finge	Protein Co	41	GC20P049	7.22
BBC3	BCL2 Bind	Protein Co	43	GC19M047	7.2
PDGFRA	Platelet De	Protein Co	56	GC04P054	7.2
GPBAR1	G Protein-	Protein Co	41	GC02P218	7.2
TMEM201	Transmem	Protein Co	36	GC01P009	7.19
CFLAR	CASP8 And	Protein Co	48	GC02P201	7.19
EGR1	Early Grow	Protein Co	45	GC05P138	7.18
SLC26A3	Solute Car	Protein Co	47	GC07M107	7.16
XBP1	X-Box Bind	Protein Co	47	GC22M028	7.14
MIR125A	MicroRNA	RNA Gene	21	GC19P051	7.14
CTSB	Cathepsin	Protein Co	52	GC08M011	7.14
EPHX1	Epoxide H	Protein Co	48	GC01P225	7.13
FHIT	Fragile His	Protein Co	46	GC03M059	7.12
CDC42	Cell Divisio	Protein Co	53	GC01P022	7.11
PROCR	Protein C I	Protein Co	44	GC20P035	7.1
KRT8	Keratin 8	Protein Co	48	GC12M052	7.09
MDM2	MDM2 Pro	Protein Co	54	GC12P068	7.08
MTOR	Mechanist	Protein Co	54	GC01M011	7.06
C3	Compleme	Protein Co	49	GC19M006	7.05
AK2	Adenylate	Protein Co	50	GC01M033	7.05
EPB42	Erythrocyt	Protein Co	40	GC15M043	7.05
CALR	Calreticulin	Protein Co	52	GC19P012	7
FLT1	Fms Relate	Protein Co	52	GC13M028	6.99
CD274	CD274 Mo	Protein Co	46	GC09P005	6.99
TRAF6	TNF Recep	Protein Co	48	GC11M036	6.98
TLR10	Toll Like R	Protein Co	42	GC04M038	6.97
NAT1	N-Acetyltr	Protein Co	46	GC08P018	6.97
MIR17	MicroRNA	RNA Gene	21	GC13P091	6.96
NFATC1	Nuclear Fa	Protein Co	49	GC18P079	6.95
MIR144	MicroRNA	RNA Gene	17	GC17M029	6.94
MIR3936	MicroRNA	RNA Gene	14	GC05M132	6.92
REG3A	Regenerat	Protein Co	40	GC02M079	6.91
LRP6	LDL Recep	Protein Co	49	GC12M013	6.91
GGT1	Gamma-G	Protein Co	48	GC22P024	6.91
LRRK2	Leucine Ri	Protein Co	51	GC12P040	6.9
PRF1	Perforin 1	Protein Co	47	GC10M070	6.9
MIR30A	MicroRNA	RNA Gene	20	GC06M071	6.89
LGALS4	Galectin 4	Protein Co	40	GC19M041	6.87
FUT3	Fucosyltra	Protein Co	44	GC19M005	6.87
ATM	ATM Serin	Protein Co	55	GC11P108	6.86
CRHR2	Corticotro	Protein Co	43	GC07M030	6.85
DNMT3B	DNA Meth	Protein Co	51	GC20P032	6.85
MUC16	Mucin 16,	Protein Co	38	GC19M008	6.84
TGFBR2	Transform	Protein Co	54	GC03P030	6.82
MMP12	Matrix Me	Protein Co	45	GC11M102	6.82
MTR	5-Methylte	Protein Co	47	GC01P236	6.82
GAS5	Growth Ar	RNA Gene	24	GC01M174	6.81
DAPK1	Death Ass	Protein Co	50	GC09P087	6.81
ADA	Adenosine	Protein Co	51	GC20M044	6.79

BLOC1S6	Biogenesis	Protein Co	39	GC15P045	6.78
MIR9-1	MicroRNA	RNA Gene	21	GC01M156	6.77
MIR199A1	MicroRNA	RNA Gene	20	GC19M010	6.77
CRH	Corticotro	Protein Co	45	GC08M066	6.77
IL25	Interleukin	Protein Co	40	GC14P025	6.75
C1S	Compleme	Protein Co	47	GC12P007	6.74
SULT1A1	Sulfotransf	Protein Co	44	GC16M028	6.74
CDK2	Cyclin Dep	Protein Co	54	GC12P055	6.73
TYMP	Thymidine	Protein Co	48	GC22M050	6.72
CDK4	Cyclin Dep	Protein Co	56	GC12M057	6.72
FGF10	Fibroblast	Protein Co	48	GC05M044	6.7
ESR2	Estrogen R	Protein Co	51	GC14M064	6.69
OR5V1	Olfactory F	Protein Co	33	GC06M029	6.68
NCAM1	Neural Cel	Protein Co	47	GC11P112	6.67
CFHR3	Compleme	Protein Co	40	GC01P196	6.66
CFHR5	Compleme	Protein Co	40	GC01P196	6.66
PSMD4	Proteasom	Protein Co	45	GC01P151	6.66
IL4R	Interleukin	Protein Co	49	GC16P027	6.63
CCT5	Chaperoni	Protein Co	45	GC05P010	6.62
APEX1	Apurinic/A	Protein Co	47	GC14P020	6.62
MIR19A	MicroRNA	RNA Gene	19	GC13P091	6.61
IFNGR2	Interferon	Protein Co	44	GC21P033	6.61
SP1	Sp1 Transc	Protein Co	45	GC12P053	6.61
IL12RB1	Interleukin	Protein Co	46	GC19M018	6.58
TDGF1	Teratocarc	Protein Co	43	GC03P046	6.57
CCKBR	Cholecysto	Protein Co	46	GC11P006	6.56
ITGA2	Integrin Su	Protein Co	47	GC05P052	6.56
NQO1	NAD(P)H (	Protein Co	51	GC16M069	6.55
U2AF1	U2 Small N	Protein Co	42	GC21M043	6.55
NF1	Neurofibro	Protein Co	50	GC17P031	6.54
SLAMF1	Signaling L	Protein Co	41	GC01M160	6.54
PPM1L	Protein Ph	Protein Co	39	GC03P160	6.52
ADGRE3	Adhesion (	Protein Co	34	GC19M014	6.52
RNF186	Ring Finge	Protein Co	33	GC01M019	6.5
TXN	Thioredoxi	Protein Co	47	GC09M110	6.49
HLA-DPB1	Major Hist	Protein Co	45	GC06P033	6.47
BMP4	Bone Morp	Protein Co	50	GC14M053	6.46
PROM1	Prominin 1	Protein Co	46	GC04M015	6.45
SNAI1	Snail Famil	Protein Co	45	GC20P049	6.44
HLA-E	Major Hist	Protein Co	42	GC06P033	6.44
MIR124-1	MicroRNA	RNA Gene	21	GC08M009	6.42
EXO1	Exonucleas	Protein Co	44	GC01P241	6.4
COX5A	Cytochron	Protein Co	44	GC15M074	6.4
CFI	Compleme	Protein Co	48	GC04M109	6.39
KLF4	Kruppel Lil	Protein Co	47	GC09M107	6.39
PNLIP	Pancreatic	Protein Co	48	GC10P116	6.38
DLAT	Dihydrolip	Protein Co	47	GC11P112	6.38
CFHR4	Compleme	Protein Co	36	GC01P196	6.38
TGFBR1	Transform	Protein Co	54	GC09P099	6.37
HSPA8	Heat Shoc	Protein Co	48	GC11M123	6.34
CD59	CD59 Mole	Protein Co	48	GC11M033	6.34
CRHR1	Corticotro	Protein Co	46	GC17P045	6.33
MIR320A	MicroRNA	RNA Gene	20	GC08M022	6.32
FCGR1A	Fc Fragme	Protein Co	43	GC01P149	6.32
PPARA	Peroxisom	Protein Co	47	GC22P046	6.32
TIMP3	TIMP Meta	Protein Co	47	GC22P032	6.29
CBR3-AS1	CBR3 Antis	RNA Gene	17	GC21M036	6.29
ITPA	Inosine Tri	Protein Co	47	GC20P003	6.28

MIR200A	MicroRNA	RNA Gene	22	GC01P001	6.27
MIRLET7E	MicroRNA	RNA Gene	21	GC19P051	6.27
UBE4A	Ubiquitina	Protein Co	40	GC11P118	6.27
IGFBP3	Insulin Like	Protein Co	47	GC07M045	6.26
CD209	CD209 Mc	Protein Co	44	GC19M007	6.26
NUP210	Nucleopor	Protein Co	40	GC03M015	6.26
CLDN7	Claudin 7	Protein Co	43	GC17M007	6.26
F11R	F11 Recep	Protein Co	45	GC01M160	6.25
CYLD	CYLD Lysir	Protein Co	49	GC16P050	6.24
RETN	Resistin	Protein Co	45	GC19P007	6.23
AGER	Advanced	Protein Co	45	GC06M032	6.21
RPS6KB1	Ribosomal	Protein Co	51	GC17P059	6.2
SLC2A1	Solute Car	Protein Co	54	GC01M042	6.19
MLKL	Mixed Line	Protein Co	42	GC16M074	6.18
FADD	Fas Associ	Protein Co	48	GC11P070	6.18
ERCC6	ERCC Excis	Protein Co	46	GC10M049	6.17
MIR23A	MicroRNA	RNA Gene	21	GC19M013	6.17
MIR196A1	MicroRNA	RNA Gene	20	GC17M048	6.17
CNR1	Cannabinc	Protein Co	48	GC06M088	6.17
PIK3R1	Phosphoir	Protein Co	52	GC05P068	6.17
JAK1	Janus Kina	Protein Co	52	GC01M064	6.16
MIR4435-2	MIR4435-2	RNA Gene	17	GC02M111	6.16
MIR4284	MicroRNA	RNA Gene	15	GC07P073	6.16
CCNA2	Cyclin A2	Protein Co	46	GC04M122	6.16
UGT1A1	UDP Glucu	Protein Co	50	GC02P233	6.16
FASN	Fatty Acid	Protein Co	51	GC17M082	6.16
TPI1	Triosephos	Protein Co	48	GC12P006	6.15
ALPI	Alkaline Pl	Protein Co	44	GC02P232	6.15
MIR642A	MicroRNA	RNA Gene	17	GC19P045	6.13
WAS	WASP Acti	Protein Co	50	GC0XP048	6.12
YAP1	Yes Associ	Protein Co	49	GC11P102	6.11
CHEK2	Checkpoin	Protein Co	55	GC22M028	6.09
KIT	KIT Proto-	Protein Co	54	GC04P054	6.08
CCL24	C-C Motif	Protein Co	38	GC07M075	6.08
EDN1	Endothelir	Protein Co	48	GC06P012	6.07
LRP5	LDL Recep	Protein Co	50	GC11P068	6.07
MIR15A	MicroRNA	RNA Gene	16	GC13M050	6.07
XRCC6	X-Ray Rep	Protein Co	47	GC22P041	6.06
TF	Transferrir	Protein Co	51	GC03P133	6.06
NOS3	Nitric Oxic	Protein Co	52	GC07P150	6.06
STAT5B	Signal Trar	Protein Co	50	GC17M042	6.04
NUDT15	Nudix Hyd	Protein Co	37	GC13P048	6.04
TNFRSF11	TNF Recep	Protein Co	49	GC08M118	6.03
TRPV1	Transient I	Protein Co	47	GC17M003	6.02
HLA-DRB9	Major Hist	Pseudoger	13	GC06M032	6.02
IFIH1	Interferon	Protein Co	49	GC02M162	6.01
FOXO3	Forkhead I	Protein Co	45	GC06P108	5.98
CD36	CD36 Mol	Protein Co	50	GC07P080	5.98
MIR10B	MicroRNA	RNA Gene	21	GC02P176	5.97
MIR139	MicroRNA	RNA Gene	20	GC11M072	5.97
MIR338	MicroRNA	RNA Gene	18	GC17M081	5.97
MIR18A	MicroRNA	RNA Gene	18	GC13P091	5.97
IL21R	Interleukin	Protein Co	43	GC16P027	5.95
CDX1	Caudal Typ	Protein Co	37	GC05P150	5.94
XRCC5	X-Ray Rep	Protein Co	45	GC02P216	5.94
CX3CL1	C-X3-C M	Protein Co	44	GC16P057	5.94
ARG1	Arginase 1	Protein Co	51	GC06P131	5.94
MRAP	Melanocoi	Protein Co	37	GC21P032	5.94

ALG9	ALG9 Alph	Protein Co	43	GC11M11[illegible]	5.93
PLA2G4F	Phospholi[illegible]	Protein Co	40	GC15M04[illegible]	5.93
EPC1	Enhancer (	Protein Co	39	GC10M03[illegible]	5.93
METTL9	Methyltrar	Protein Co	35	GC16P021	5.93
IGSF6-DRE	Region Co	Uncategor	2	GC16U900	5.93
KAT5	Lysine Ace	Protein Co	48	GC11P065	5.93
TSLP	Thymic Str	Protein Co	40	GC05P111	5.92
CD69	CD69 Mol	Protein Co	42	GC12M01[illegible]	5.92
BACH2	BTB Doma	Protein Co	43	GC06M08[illegible]	5.9
ADORA3	Adenosine	Protein Co	47	GC01M11[illegible]	5.89
AFAP1-AS	AFAP1 Ant	RNA Gene	17	GC04P007	5.88
TCN2	Transcoba	Protein Co	45	GC22P030	5.88
AMBP	Alpha-1-M	Protein Co	43	GC09M11[illegible]	5.87
SOD2	Superoxid	Protein Co	52	GC06M15[illegible]	5.87
TGFB3	Transform	Protein Co	48	GC14M07[illegible]	5.83
SFRP2	Secreted F	Protein Co	44	GC04M15[illegible]	5.8
GUCA2A	Guanylate	Protein Co	37	GC01M04[illegible]	5.8
NLRC4	NLR Family	Protein Co	45	GC02M03[illegible]	5.8
ITK	IL2 Inducib	Protein Co	53	GC05P157	5.79
TGFB2	Transform	Protein Co	51	GC01P218	5.79
DNMT3A	DNA Meth	Protein Co	52	GC02M02[illegible]	5.77
ERCC1	ERCC Excis	Protein Co	47	GC19M04[illegible]	5.77
WNT2	Wnt Family	Protein Co	45	GC07M11[illegible]	5.77
MALAT1	Metastasis	RNA Gene	25	GC11P065	5.77
NEAT1	Nuclear Pa	RNA Gene	24	GC11P065	5.77
TP73	Tumor Pro	Protein Co	47	GC01P003	5.77
GPX1	Glutathion	Protein Co	49	GC03M04[illegible]	5.76
MIR149	MicroRNA	RNA Gene	21	GC02P240	5.76
MIRLET7I	MicroRNA	RNA Gene	20	GC12P062	5.76
MIR675	MicroRNA	RNA Gene	17	GC11M00[illegible]	5.76
CYP1B1	Cytochron	Protein Co	50	GC02M03[illegible]	5.75
LOX	Lysyl Oxid	Protein Co	46	GC05M12[illegible]	5.75
MIR29B1	MicroRNA	RNA Gene	22	GC07M13[illegible]	5.74
PON1	Paraoxona	Protein Co	47	GC07M09[illegible]	5.73
ALK	ALK Recep	Protein Co	52	GC02M02[illegible]	5.72
DST	Dystonin	Protein Co	43	GC06M05[illegible]	5.72
AGT	Angiotens	Protein Co	51	GC01M23[illegible]	5.7
MIR146B	MicroRNA	RNA Gene	20	GC10P102	5.7
CHEK1	Checkpoin	Protein Co	51	GC11P125	5.7
GUCA2B	Guanylate	Protein Co	40	GC01P042	5.7
KDM4C	Lysine Der	Protein Co	41	GC09P006	5.69
VWA2	Von Willeb	Protein Co	36	GC10P114	5.69
SEPSECS	Sep (O-Ph	Protein Co	45	GC04M02[illegible]	5.68
FURIN	Furin, Pair	Protein Co	48	GC15P090	5.68
TNFRSF6B	TNF Recep	Protein Co	44	GC20P063	5.67
AGR2	Anterior G	Protein Co	42	GC07M01[illegible]	5.67
OTUD3	OTU Deub	Protein Co	35	GC01P019	5.67
ALOX15	Arachidon	Protein Co	45	GC17M00[illegible]	5.66
DENND1B	DENN Dor	Protein Co	36	GC01M19[illegible]	5.65
MMP8	Matrix Me	Protein Co	48	GC11M10[illegible]	5.63
LAMB1	Laminin Su	Protein Co	49	GC07M10[illegible]	5.61
BMP7	Bone Mor	Protein Co	46	GC20M05[illegible]	5.6
BECN1	Beclin 1	Protein Co	48	GC17M04[illegible]	5.59
CSF1	Colony Sti	Protein Co	45	GC01P109	5.59
SNAI2	Snail Famil	Protein Co	46	GC08M04[illegible]	5.59
KRT15	Keratin 15	Protein Co	41	GC17M04[illegible]	5.58
DSG2	Desmoglei	Protein Co	47	GC18P031	5.58
TOP1	DNA Topo	Protein Co	48	GC20P041	5.56

CYP2E1	Cytochron	Protein Co	47	GC10P133	5.56
SLC17A5	Solute Car	Protein Co	45	GC06M07:	5.55
ANXA2	Annexin A	Protein Co	49	GC15M06(	5.54
TWIST1	Twist Fami	Protein Co	46	GC07M01	5.54
CDKN2B-/	CDKN2B A	RNA Gene	22	GC09P021	5.54
NPSR1	Neuropep	Protein Co	41	GC07P034	5.53
JAG1	Jagged Ca	Protein Co	51	GC20M01(	5.52
MIR181A1	MicroRNA	RNA Gene	20	GC01M19	5.52
MIR27B	MicroRNA	RNA Gene	21	GC09P095	5.52
EPHA3	EPH Recep	Protein Co	46	GC03P089	5.51
GLI3	GLI Family	Protein Co	50	GC07M04:	5.51
CD86	CD86 Mol	Protein Co	45	GC03P122	5.5
GRB2	Growth Fa	Protein Co	51	GC17M07	5.5
HTR4	5-Hydroxy	Protein Co	45	GC05M14	5.5
ATG5	Autophag	Protein Co	45	GC06M10(	5.49
NHEJ1	Non-Hom	Protein Co	43	GC02M21	5.49
FCGR2B	Fc Fragme	Protein Co	49	GC01P161	5.49
GSTT1	Glutathion	Protein Co	35	GC22Mi00	5.48
FZD8	Frizzled Cl	Protein Co	42	GC10M03	5.46
F13A1	Coagulatic	Protein Co	47	GC06M00(	5.45
MIR598	MicroRNA	RNA Gene	17	GC08M01:	5.44
CCN4	Cellular Cc	Protein Co	33	GC08P133	5.44
CLDN8	Claudin 8	Protein Co	36	GC21M03(	5.44
RASSF1	Ras Associ	Protein Co	45	GC03M05(	5.44
BRINP3	BMP/Retin	Protein Co	29	GC01M19(	5.42
BMI1	BMI1 Prot	Protein Co	45	GC10P022	5.42
THBS1	Thrombos	Protein Co	45	GC15P039	5.4
PRRT2	Proline Ric	Protein Co	40	GC16P029	5.4
CDK5RAP1	CDK5 Reg	Protein Co	38	GC20M03:	5.4
ALLC	Allantoica	Protein Co	36	GC02P003	5.4
STX11	Syntaxin 1	Protein Co	41	GC06P144	5.39
FSCN1	Fascin Acti	Protein Co	44	GC07P005	5.38
EZH2	Enhancer (	Protein Co	54	GC07M14	5.37
HSPA5	Heat Shoc	Protein Co	48	GC09M12	5.33
MIR92A1	MicroRNA	RNA Gene	20	GC13P091	5.33
LGALS3BP	Galectin 3	Protein Co	42	GC17M07	5.31
SLIT2	Slit Guidar	Protein Co	47	GC04P020	5.3
GSDMB	Gasdermir	Protein Co	37	GC17M03	5.3
LINC01475	Long Inter	RNA Gene	12	GC10M09	5.28
MUC17	Mucin 17,	Protein Co	36	GC07P101	5.28
ABCB11	ATP Bindir	Protein Co	46	GC02M16	5.27
FGF19	Fibroblast	Protein Co	45	GC11M06	5.27
SP100	SP100 Nuc	Protein Co	42	GC02P230	5.27
BLOC1S1	Biogenesis	Protein Co	37	GC12P055	5.27
GGT2	Gamma-G	Protein Co	32	GC22M02:	5.27
GGTLC3	Gamma-G	Protein Co	21	GC22M01	5.27
MIR182	MicroRNA	RNA Gene	20	GC07M12	5.27
MIR103A1	MicroRNA	RNA Gene	18	GC05M16	5.27
RAD51	RAD51 Re	Protein Co	52	GC15P040	5.27
BRCA1	BRCA1 DN	Protein Co	52	GC17M04:	5.27
WNT3	Wnt Famil	Protein Co	48	GC17M04(	5.27
ANTXR1	ANTXR Ce	Protein Co	47	GC02P068	5.27
SLAMF6	SLAM Fam	Protein Co	41	GC01M16(	5.27
DACT1	Dishevelle	Protein Co	40	GC14P058	5.27
MIR130B	MicroRNA	RNA Gene	18	GC22P023	5.27
CTNNA1	Catenin Al	Protein Co	49	GC05P138	5.26
CTNND1	Catenin De	Protein Co	47	GC11P057	5.26
CYP7A1	Cytochron	Protein Co	43	GC08M05	5.26

SIRT1	Sirtuin 1	Protein Co	51	GC10P067	5.25
PLA2G2E	Phospholi	Protein Co	40	GC01M01	5.25
ERGIC1	Endoplasm	Protein Co	40	GC05P172	5.25
HLA-DRB5	Major Hist	Protein Co	40	GC06M032	5.24
SULT2A1	Sulfotrans	Protein Co	46	GC19M047	5.23
SLC10A2	Solute Car	Protein Co	41	GC13M103	5.23
KNG1	Kininogen	Protein Co	45	GC03P186	5.23
HPS4	HPS4 Biog	Protein Co	40	GC22M026	5.22
CXCR1	C-X-C Mo	Protein Co	44	GC02M218	5.22
SERPINB5	Serpin Fan	Protein Co	45	GC18P063	5.21
ALPP	Alkaline Pl	Protein Co	46	GC02P232	5.21
GLI1	GLI Family	Protein Co	48	GC12P057	5.2
LY9	Lymphocy	Protein Co	40	GC01P160	5.18
PVT1	Pvt1 Onco	RNA Gene	26	GC08P127	5.18
HOTTIP	HOXA Dist	RNA Gene	24	GC07P027	5.18
HOXA11-A	HOXA11 A	RNA Gene	20	GC07P027	5.18
PKM	Pyruvate K	Protein Co	47	GC15M072	5.18
RAF1	Raf-1 Prot	Protein Co	56	GC03M012	5.17
WNT3A	Wnt Family	Protein Co	49	GC01P228	5.16
STXBP2	Syntaxin B	Protein Co	46	GC19P007	5.15
RAB27A	RAB27A, M	Protein Co	50	GC15M055	5.15
RECK	Reversion	Protein Co	42	GC09P036	5.13
MUC19	Mucin 19,	Protein Co	29	GC12P040	5.13
FLT4	Fms Relate	Protein Co	54	GC05M180	5.1
MDH2	Malate De	Protein Co	50	GC07P076	5.09
ENPP7	Ectonucleo	Protein Co	39	GC17P079	5.09
UGT1A6	UDP Glucu	Protein Co	41	GC02P233	5.08
ITGAX	Integrin Su	Protein Co	45	GC16P031	5.07
MAPK9	Mitogen-A	Protein Co	50	GC05M180	5.07
BSN	Bassoon P	Protein Co	36	GC03P049	5.07
RDX	Radixin	Protein Co	50	GC11M109	5.06
MIR423	MicroRNA	RNA Gene	18	GC17P030	5.06
ABCB4	ATP Bindir	Protein Co	46	GC07M087	5.05
STK11	Serine/Thr	Protein Co	50	GC19P001	5.04
PSMG1	Proteasom	Protein Co	38	GC21M039	5.04
SOX2	SRY-Box T	Protein Co	48	GC03P181	5.04
CCNB1	Cyclin B1	Protein Co	48	GC05P069	5.04
ERCC2	ERCC Excis	Protein Co	48	GC19M045	5.04
WNT16	Wnt Family	Protein Co	41	GC07P121	5.04
HFM1	Helicase F	Protein Co	40	GC01M091	5.04
SDSL	Serine Deh	Protein Co	40	GC12P113	5.04
MIR132	MicroRNA	RNA Gene	22	GC17M002	5
SETDB1	SET Doma	Protein Co	43	GC01P150	5
PCCA	Propionyl-	Protein Co	47	GC13P100	4.99
SLC10A1	Solute Car	Protein Co	44	GC14M069	4.99
DNAL1	Dynein Ax	Protein Co	43	GC14P073	4.99
SCTR	Secretin R	Protein Co	42	GC02M119	4.99
CYP8B1	Cytochron	Protein Co	41	GC03M042	4.99
ATP8B1	ATPase Ph	Protein Co	41	GC18M057	4.99
LCOR	Ligand De	Protein Co	39	GC10P096	4.99
DDX53	DEAD-Box	Protein Co	34	GC0XP022	4.99
MT-CYB	Mitochond	Protein Co	32	GCMTP014	4.99
MIRLET7B	MicroRNA	RNA Gene	21	GC22P046	4.99
MIR542	MicroRNA	RNA Gene	17	GC0XM134	4.99
MIR1537	MicroRNA	RNA Gene	14	GC01M235	4.99
G6PC3	Glucose-6	Protein Co	42	GC17P044	4.97
SYNE1	Spectrin R	Protein Co	42	GC06M152	4.97
MMP26	Matrix Me	Protein Co	35	GC11P004	4.95

MSN	Moesin	Protein Co	48	GC0XP065	4.94
CDK1	Cyclin Dep	Protein Co	47	GC10P060	4.94
SLC44A4	Solute Car	Protein Co	41	GC06M031	4.94
LMNA	Lamin A/C	Protein Co	49	GC01P156	4.93
ZNF148	Zinc Finge	Protein Co	41	GC03M125	4.93
PUS10	Pseudouri	Protein Co	36	GC02M060	4.92
MIR30C1	MicroRNA	RNA Gene	22	GC01P040	4.91
PTGER2	Prostaglan	Protein Co	49	GC14P052	4.9
EDNRA	Endothelir	Protein Co	50	GC04P147	4.9
NRON	Non-Codi	RNA Gene	18	GC09M126	4.89
IRF8	Interferon	Protein Co	47	GC16P085	4.89
MIR185	MicroRNA	RNA Gene	22	GC22P020	4.86
MIR99A	MicroRNA	RNA Gene	21	GC21P016	4.86
MIR486-1	MicroRNA	RNA Gene	17	GC08M041	4.86
GJB6	Gap Juncti	Protein Co	45	GC13M020	4.85
AXIN1	Axin 1	Protein Co	48	GC16M000	4.85
MRE11	MRE11 Ho	Protein Co	43	GC11M094	4.85
COMT	Catechol-(	Protein Co	52	GC22P019	4.85
WNT5A	Wnt Family	Protein Co	51	GC03M055	4.85
MMP11	Matrix Me	Protein Co	47	GC22P023	4.85
FABP2	Fatty Acid	Protein Co	44	GC04M119	4.85
APOB	Apolipopr	Protein Co	45	GC02M020	4.84
SOX9	SRY-Box T	Protein Co	48	GC17P072	4.84
CEACAM1	CEA Cell A	Protein Co	43	GC19M042	4.84
WNT4	Wnt Family	Protein Co	48	GC01M022	4.81
HBEGF	Heparin Bi	Protein Co	44	GC05M140	4.81
DVL1	Dishevelle	Protein Co	48	GC01M001	4.8
MAP2K5	Mitogen-A	Protein Co	48	GC15P071	4.8
MAPRE1	Microtubu	Protein Co	45	GC20P032	4.8
HOTAIRM1	HOXA Trar	RNA Gene	20	GC07P027	4.8
SCARNA5	Small Caja	RNA Gene	19	GC02P233	4.8
LINC00460	Long Inter	RNA Gene	17	GC13P106	4.8
CCNY	Cyclin Y	Protein Co	37	GC10P035	4.8
MUC20	Mucin 20,	Protein Co	39	GC03P195	4.79
CSK	C-Termina	Protein Co	49	GC15P074	4.79
WNT1	Wnt Family	Protein Co	48	GC12P049	4.79
LEF1	Lymphoid	Protein Co	48	GC04M108	4.79
MAP2K4	Mitogen-A	Protein Co	47	GC17P012	4.79
WNT2B	Wnt Family	Protein Co	45	GC01P112	4.79
VEGFD	Vascular E	Protein Co	36	GC0XM015	4.79
HOTAIR	HOX Trans	RNA Gene	25	GC12M053	4.79
CYP24A1	Cytochron	Protein Co	48	GC20M054	4.78
HTR2A	5-Hydroxy	Protein Co	49	GC13M046	4.78
ZEB1	Zinc Finge	Protein Co	50	GC10P031	4.77
P2RX7	Purinergic	Protein Co	47	GC12P122	4.77
TREX1	Three Prim	Protein Co	44	GC03P048	4.76
RARB	Retinoic A	Protein Co	51	GC03P025	4.75
ABCC1	ATP Bindir	Protein Co	48	GC16P015	4.75
GDF15	Growth Di	Protein Co	43	GC19P022	4.75
STAT5A	Signal Trar	Protein Co	47	GC17P042	4.74
HAVCR2	Hepatitis A	Protein Co	45	GC05M157	4.74
MIR22	MicroRNA	RNA Gene	20	GC17M001	4.74
RAG2	Recombina	Protein Co	44	GC11M036	4.73
CDH2	Cadherin 2	Protein Co	51	GC18M027	4.72
OPRM1	Opioid Rec	Protein Co	50	GC06P154	4.71
SFRP1	Secreted F	Protein Co	44	GC08M041	4.71
RSPO1	R-Spondir	Protein Co	45	GC01M037	4.71
CEACAM7	CEA Cell A	Protein Co	39	GC19M041	4.71

MAF	MAF BZIP	Protein Co	47	GC16M079	4.68
MIR224	MicroRNA	RNA Gene	18	GC0XM151	4.68
ATF3	Activating	Protein Co	45	GC01P212	4.67
NAMPT	Nicotinam	Protein Co	50	GC07M106	4.67
ZFP36L1	ZFP36 Ring	Protein Co	44	GC14M068	4.67
SLAMF7	SLAM Fam	Protein Co	43	GC01P160	4.67
ADAM30	ADAM Me	Protein Co	36	GC01M119	4.67
FIP1L1	Factor Inte	Protein Co	39	GC04P053	4.66
CYBA	Cytochrom	Protein Co	47	GC16M088	4.65
ITGA6	Integrin Su	Protein Co	51	GC02P172	4.65
ANTXR2	ANTXR Ce	Protein Co	48	GC04M079	4.64
TYR	Tyrosinase	Protein Co	48	GC11P089	4.63
SERPINH1	Serpin Fan	Protein Co	47	GC11P075	4.63
IGFBP2	Insulin Like	Protein Co	45	GC02P216	4.62
CASP5	Caspase 5	Protein Co	45	GC11M104	4.62
PDCD4	Programm	Protein Co	44	GC10P110	4.62
TRAF3IP2	TRAF3 Inte	Protein Co	45	GC06M111	4.62
VEGFC	Vascular E	Protein Co	48	GC04M176	4.61
CCL21	C-C Motif	Protein Co	44	GC09M034	4.6
TNFRSF14	TNF Recep	Protein Co	45	GC01P002	4.6
NME1	NME/NM2	Protein Co	49	GC17P051	4.59
MSH3	MutS Hom	Protein Co	44	GC05P080	4.59
ELAVL1	ELAV Like	Protein Co	43	GC19M007	4.59
ZPBP2	Zona Pellu	Protein Co	36	GC17P039	4.59
IGHE	Immunogl	Protein Co	28	GC14M105	4.59
DHFR	Dihydrofol	Protein Co	51	GC05M080	4.58
UNC13D	Unc-13 Hc	Protein Co	44	GC17M075	4.58
ABCA1	ATP Bindir	Protein Co	49	GC09M104	4.58
TAP1	Transporte	Protein Co	48	GC06M032	4.58
CREB1	CAMP Res	Protein Co	50	GC02P207	4.57
CDK6	Cyclin Dep	Protein Co	55	GC07M092	4.57
PRODH	Proline De	Protein Co	47	GC22M018	4.57
ERBB4	Erb-B2 Re	Protein Co	56	GC02M211	4.57
YES1	YES Proto-	Protein Co	50	GC18M000	4.57
UGT1A7	UDP Gluc	Protein Co	39	GC02P233	4.57
MIRLET7F1	MicroRNA	RNA Gene	20	GC09P094	4.57
HTR1A	5-Hydroxy	Protein Co	48	GC05M063	4.56
CDKAL1	CDK5 Reg	Protein Co	39	GC06P020	4.55
CD27	CD27 Mol	Protein Co	47	GC12P006	4.55
CEACAM3	CEA Cell A	Protein Co	41	GC19P041	4.53
FCAR	Fc Fragme	Protein Co	43	GC19P055	4.52
ICOS	Inducible T	Protein Co	44	GC02P203	4.52
NRP1	Neuropilin	Protein Co	48	GC10M033	4.51
RHOD	Ras Homo	Protein Co	40	GC11P067	4.51
ERBB3	Erb-B2 Re	Protein Co	55	GC12P056	4.51
EPHB2	EPH Recep	Protein Co	53	GC01P022	4.51
PIK3R2	Phosphoir	Protein Co	51	GC19P018	4.51
CDC25C	Cell Divisic	Protein Co	50	GC05M138	4.51
OGG1	8-Oxogua	Protein Co	48	GC03P009	4.51
RAG1	Recombin	Protein Co	47	GC11P036	4.51
CA9	Carbonic A	Protein Co	46	GC09P035	4.51
E2F1	E2F Transc	Protein Co	45	GC20M033	4.51
WNT5B	Wnt Family	Protein Co	45	GC12P001	4.51
WNT11	Wnt Family	Protein Co	45	GC11M076	4.51
TBX1	T-Box Trar	Protein Co	44	GC22P019	4.51
CHSY1	Chondroiti	Protein Co	44	GC15M101	4.51
HAVCR1	Hepatitis A	Protein Co	43	GC05M157	4.51
XRCC3	X-Ray Rep	Protein Co	42	GC14M103	4.51

NAT9	N-Acetyltr	Protein Co	37	GC17M07	4.51
PRDM10	PR/SET Dc	Protein Co	35	GC11M12	4.51
MIR191	MicroRNA	RNA Gene	21	GC03M04	4.51
MIR345	MicroRNA	RNA Gene	20	GC14P100	4.51
MIR454	MicroRNA	RNA Gene	19	GC17M05	4.51
MIR296	MicroRNA	RNA Gene	18	GC20M05	4.51
CYTOR	Cytoskelet	RNA Gene	18	GC02P087	4.51
MIR106A	MicroRNA	RNA Gene	18	GC0XM13	4.51
SPRY4-IT1	SPRY4 Intr	RNA Gene	10	GC05U901	4.51
DSP	Desmoplal	Protein Co	51	GC06P007	4.51
IKZF1	IKAROS Fa	Protein Co	48	GC07P050	4.5
PTK2	Protein Ty	Protein Co	49	GC08M14	4.5
SETD2	SET Doma	Protein Co	47	GC03M04	4.5
ECM1	Extracellul	Protein Co	45	GC01P150	4.48
PRKCA	Protein Kir	Protein Co	52	GC17P066	4.48
SERPINB2	Serpin Fan	Protein Co	45	GC18P063	4.48
KIAA1109	KIAA1109	Protein Co	35	GC04P122	4.48
BANK1	B Cell Scaf	Protein Co	37	GC04P101	4.47
TPO	Thyroid Pe	Protein Co	49	GC02P001	4.47
MUC7	Mucin 7, S	Protein Co	39	GC04P070	4.47
MIR30E	MicroRNA	RNA Gene	21	GC01P040	4.47
SI	Sucrase-Is	Protein Co	44	GC03M16	4.47
TRPA1	Transient	Protein Co	45	GC08M07	4.46
GSK3B	Glycogen	Protein Co	51	GC03M11	4.44
ANGPT1	Angiopoie	Protein Co	47	GC08M10	4.44
PRKD1	Protein Kir	Protein Co	51	GC14M02	4.44
IFI27	Interferon	Protein Co	39	GC14P094	4.43
NOTCH4	Notch Rec	Protein Co	47	GC06M03	4.43
LAMP1	Lysosomal	Protein Co	44	GC13P113	4.43
MIR574	MicroRNA	RNA Gene	19	GC04P038	4.41
MIR455	MicroRNA	RNA Gene	18	GC09P114	4.41
MIR135B	MicroRNA	RNA Gene	20	GC01M20	4.41
LITAF	Lipopolysa	Protein Co	45	GC16M01	4.4
TKT	Transketol	Protein Co	49	GC03M05	4.4
PIK3CB	Phosphatid	Protein Co	48	GC03M13	4.38
TOP2A	DNA Topc	Protein Co	51	GC17M04	4.38
DLD	Dihydrolip	Protein Co	51	GC07P107	4.36
MST1R	Macropha	Protein Co	50	GC03M04	4.35
TOLLIP	Toll Intera	Protein Co	45	GC11M00	4.34
FZD7	Frizzled Cl	Protein Co	45	GC02P202	4.32
BLK	BLK Proto-	Protein Co	52	GC08P011	4.32
AOC1	Amine Oxi	Protein Co	41	GC07P150	4.32
ILK	Integrin Li	Protein Co	48	GC11P006	4.31
ADAD1	Adenosine	Protein Co	35	GC04P122	4.3
BCL2L11	BCL2 Like	Protein Co	47	GC02P111	4.3
CCND3	Cyclin D3	Protein Co	49	GC06M04	4.28
IL20	Interleukin	Protein Co	41	GC01P206	4.28
SMURF1	SMAD Spe	Protein Co	47	GC07M09	4.27
GNA12	G Protein	Protein Co	43	GC07M00	4.26
GATD3A	Glutamine	Protein Co	27	GC21P044	4.26
IL2RG	Interleukin	Protein Co	50	GC0XM07	4.25
BAD	BCL2 Asso	Protein Co	48	GC11M06	4.25
UMPS	Uridine M	Protein Co	46	GC03P124	4.25
CD82	CD82 Mol	Protein Co	44	GC11P044	4.25
TUG1	Taurine Up	RNA Gene	24	GC22P030	4.25
UCA1	Urothelial	RNA Gene	23	GC19P015	4.25
PTENP1	Phosphata	Pseudoger	22	GC09M03	4.25
ATF6	Activating	Protein Co	49	GC01P161	4.25

EZR	Ezrin	Protein Co	47	GC06M158	4.23
TOX	Thymocyte	Protein Co	41	GC08M058	4.22
CTBP1	C-Termina	Protein Co	48	GC04M001	4.21
IL13RA2	Interleukin	Protein Co	41	GC0XM115	4.21
NTN1	Netrin 1	Protein Co	46	GC17P009	4.2
IL1RL2	Interleukin	Protein Co	43	GC02P102	4.2
AKT2	AKT Serine	Protein Co	56	GC19M040	4.19
CCND2	Cyclin D2	Protein Co	51	GC12P006	4.19
PLK1	Polo Like K	Protein Co	50	GC16P023	4.19
ZEB2	Zinc Finge	Protein Co	50	GC02M144	4.19
BAK1	BCL2 Anta	Protein Co	46	GC06M033	4.19
PIK3R3	Phosphoir	Protein Co	45	GC01M046	4.19
KIF1B	Kinesin Fa	Protein Co	45	GC01P010	4.19
S100A4	S100 Calci	Protein Co	45	GC01M153	4.19
PTGES2	Prostaglan	Protein Co	44	GC09M128	4.19
FERMT3	Fermitin Fa	Protein Co	44	GC11P064	4.19
PTPRU	Protein Ty	Protein Co	43	GC01P029	4.19
TPX2	TPX2 Micro	Protein Co	43	GC20P031	4.19
GRPR	Gastrin Re	Protein Co	43	GC0XP016	4.19
WNT6	Wnt Family	Protein Co	42	GC02P218	4.19
RUNX1T1	RUNX1 Pa	Protein Co	41	GC08M091	4.19
WNT8B	Wnt Family	Protein Co	41	GC10P100	4.19
PDPN	Podoplani	Protein Co	41	GC01P013	4.19
FERMT2	Fermitin Fa	Protein Co	39	GC14M052	4.19
PIF1	PIF1 5'-To	Protein Co	37	GC15M064	4.19
MEG3	Maternally	RNA Gene	29	GC14P104	4.19
CASC2	Cancer Sus	RNA Gene	26	GC10P118	4.19
KCNQ1OT	KCNQ1 Op	RNA Gene	25	GC11M002	4.19
SNHG12	Small Nucl	RNA Gene	24	GC01M028	4.19
FER1L4	Fer-1 Like	Pseudoger	24	GC20M035	4.19
TINCR	TINCR Ubi	RNA Gene	24	GC19M005	4.19
XIST	X Inactive	RNA Gene	24	GC0XM073	4.19
SOX2-OT	SOX2 Ove	RNA Gene	22	GC03P180	4.19
DANCR	Differentia	RNA Gene	22	GC04P052	4.19
SNHG5	Small Nucl	RNA Gene	21	GC06M085	4.19
SNHG1	Small Nucl	RNA Gene	21	GC11M063	4.19
HULC	Hepatocel	RNA Gene	21	GC06P008	4.19
CRNDE	Colorectal	Protein Co	21	GC16M054	4.19
MIR10A	MicroRNA	RNA Gene	21	GC17M048	4.19
MIR375	MicroRNA	RNA Gene	20	GC02M219	4.19
MIRLET7G	MicroRNA	RNA Gene	20	GC03M052	4.19
HNF1A-AS	HNF1A An	RNA Gene	20	GC12M121	4.19
MIR101-1	MicroRNA	RNA Gene	20	GC01M065	4.19
MIR107	MicroRNA	RNA Gene	20	GC10M089	4.19
LINC00261	Long Inter	RNA Gene	20	GC20M022	4.19
ZFAS1	ZNFX1 An	RNA Gene	20	GC20P049	4.19
PCAT1	Prostate C	RNA Gene	19	GC08P126	4.19
MIR24-2	MicroRNA	RNA Gene	19	GC19M013	4.19
RPL34-AS	RPL34 Ant	RNA Gene	19	GC04M108	4.19
SNHG16	Small Nucl	RNA Gene	18	GC17P076	4.19
SNHG20	Small Nucl	RNA Gene	18	GC17P077	4.19
HOXA-AS	HOXA Clus	RNA Gene	18	GC07P027	4.19
BCAR4	Breast Car	RNA Gene	18	GC16M011	4.19
TUSC7	Tumor Sup	RNA Gene	18	GC03P116	4.19
SNHG6	Small Nucl	RNA Gene	17	GC08M066	4.19
ZEB1-AS1	ZEB1 Antis	RNA Gene	17	GC10M031	4.19
LINC-ROR	Long Inter	RNA Gene	17	GC18M057	4.19
BLACAT1	Bladder Ca	RNA Gene	16	GC01M205	4.19

FEZF1-AS1	FEZF1 Anti	RNA Gene	16	GC07P122	4.19
CCAT1	Colon Can	RNA Gene	15	GC08M127	4.19
TMEM238L	Transmem	RNA Gene	15	GC17M010	4.19
PRNCR1	Prostate C	RNA Gene	14	GC08P127	4.19
HEIH	Hepatocel	RNA Gene	14	GC05M180	4.19
GAPLINC	Gastric Ad	RNA Gene	14	GC18P003	4.19
CCAT2	Colon Can	RNA Gene	14	GC08P127	4.19
NPTN-IT1	NPTN Intro	RNA Gene	13	GC15M073	4.19
PANDAR	Promoter (	RNA Gene	13	GC06M036	4.19
NCRUPAR	Non-Prote	RNA Gene	13	GC05P076	4.19
BANCR	BRAF-Acti	RNA Gene	13	GC09M069	4.19
GHET1	Gastric Ca	RNA Gene	12	GC07P149	4.19
DUXAP9	Double Hc	Pseudoger	9	GC14P019	4.19
TP53COR1	Tumor Prc	RNA Gene	9	GC06U903	4.19
LNCRNA-/	Long Non	RNA Gene	4	GC14U902	4.19
LOC10602	Williams-E	Biological	2	GC07U904	4.19
SERPINF2	Serpin Fan	Protein Co	45	GC17P001	4.19
TET2	Tet Methyl	Protein Co	45	GC04P105	4.18
NRIP1	Nuclear Re	Protein Co	44	GC21M014	4.17
CASP7	Caspase 7	Protein Co	51	GC10P113	4.16
MIR23B	MicroRNA	RNA Gene	21	GC09P095	4.16
MIR28	MicroRNA	RNA Gene	20	GC03P188	4.16
MIR324	MicroRNA	RNA Gene	19	GC17M007	4.16
MIR29C	MicroRNA	RNA Gene	18	GC01M207	4.16
MIR151A	MicroRNA	RNA Gene	18	GC08M140	4.16
WIF1	WNT Inhib	Protein Co	46	GC12M065	4.16
LACTB	Lactamase	Protein Co	37	GC15P071	4.14
TNFSF4	TNF Super	Protein Co	42	GC01M173	4.13
CLDN5	Claudin 5	Protein Co	43	GC22M019	4.13
ADAMTS1	ADAM Me	Protein Co	47	GC09P133	4.12
HSPA6	Heat Shoc	Protein Co	45	GC01P161	4.12
ACVR2A	Activin A F	Protein Co	46	GC02P147	4.11
PTPRT	Protein Ty	Protein Co	43	GC20M042	4.11
DNTT	DNA Nucle	Protein Co	43	GC10P096	4.1
IL1R2	Interleukin	Protein Co	47	GC02P101	4.1
HDAC2	Histone De	Protein Co	53	GC06M113	4.06
SLC29A1	Solute Car	Protein Co	48	GC06P044	4.06
IGFBP7	Insulin Like	Protein Co	47	GC04M057	4.06
STUB1	STIP1 Hon	Protein Co	47	GC16P001	4.06
PSMA7	Proteasom	Protein Co	46	GC20M062	4.06
ST6GAL1	ST6 Beta-(	Protein Co	45	GC03P186	4.06
EREG	Epiregulin	Protein Co	43	GC04P074	4.06
CLCA1	Chloride C	Protein Co	41	GC01P086	4.06
CSE1L	Chromoso	Protein Co	41	GC20P049	4.05
MT-CO2	Mitochonc	Protein Co	35	GCMTP007	4.05
THY1	Thy-1 Cell	Protein Co	45	GC11M119	4.05
LEPR	Leptin Rec	Protein Co	51	GC01P065	4.05
TFRC	Transferrir	Protein Co	50	GC03M196	4.04
MIR29B2	MicroRNA	RNA Gene	20	GC01M207	4.03
RHO	Rhodopsir	Protein Co	49	GC03P130	4.03
MB	Myoglobir	Protein Co	45	GC22M035	4.02
MTRR	5-Methylte	Protein Co	44	GC05P007	4.02
CHAT	Choline O	Protein Co	49	GC10P049	4.02
AQP8	Aquaporin	Protein Co	39	GC16P026	4.01
INPP5E	Inositol Po	Protein Co	41	GC09M136	4.01
DDIT3	DNA Dam	Protein Co	47	GC12M057	4.01
COL14A1	Collagen T	Protein Co	43	GC08P120	3.99
DES	Desmin	Protein Co	49	GC02P219	3.99

EPHX2	Epoxide H	Protein Co	49	GC08P027	3.99
MTHFD1	Methylene	Protein Co	45	GC14P064	3.97
SAG	S-Antigen	Protein Co	46	GC02P233	3.96
ERAP2	Endoplasm	Protein Co	39	GC05P096	3.96
HSPA1B	Heat Shoc	Protein Co	41	GC06P033	3.96
EPHA2	EPH Recep	Protein Co	54	GC01M016	3.95
PRKCZ	Protein Kin	Protein Co	50	GC01P002	3.95
ARPC2	Actin Relat	Protein Co	44	GC02P218	3.94
WASHC5	WASH Cor	Protein Co	33	GC08M127	3.94
ABCB5	ATP Bindir	Protein Co	43	GC07P020	3.92
GSR	Glutathion	Protein Co	50	GC08M030	3.92
TNIP1	TNFAIP3 Ir	Protein Co	42	GC05M151	3.91
CD63	CD63 Mole	Protein Co	43	GC12M055	3.91
NFKBIL1	NFKB Inhib	Protein Co	38	GC06P033	3.91
DDC	Dopa Deca	Protein Co	53	GC07M050	3.91
PPARD	Peroxisom	Protein Co	48	GC06P046	3.91
GCNT3	Glucosami	Protein Co	39	GC15P059	3.91
GADD45A	Growth Ar	Protein Co	46	GC01P067	3.9
IL17REL	Interleukin	Protein Co	28	GC22M049	3.89
WT1	WT1 Trans	Protein Co	50	GC11M032	3.88
CR1	Compleme	Protein Co	45	GC01P207	3.88
C7	Compleme	Protein Co	44	GC05P040	3.88
SIGLEC5	Sialic Acid	Protein Co	39	GC19M051	3.88
MIR615	MicroRNA	RNA Gene	20	GC12P054	3.88
MIR186	MicroRNA	RNA Gene	19	GC01M071	3.88
PRMT1	Protein Ar	Protein Co	50	GC19P049	3.88
EIF4G1	Eukaryotic	Protein Co	47	GC03P184	3.88
FBXW7	F-Box Anc	Protein Co	45	GC04M152	3.88
MTA1	Metastasis	Protein Co	44	GC14P105	3.88
TPBG	Trophobla	Protein Co	40	GC06P082	3.88
DKK1	Dickkopf V	Protein Co	47	GC10P052	3.88
FABP4	Fatty Acid	Protein Co	46	GC08M081	3.88
PTPN13	Protein Ty	Protein Co	45	GC04P086	3.88
FUT4	Fucosyltra	Protein Co	38	GC11P094	3.88
PTPA	Protein Ph	Protein Co	36	GC09P129	3.88
MIR133B	MicroRNA	RNA Gene	21	GC06P052	3.88
MIR129-2	MicroRNA	RNA Gene	19	GC11P043	3.88
MIR137	MicroRNA	RNA Gene	18	GC01M098	3.88
UBE2L3	Ubiquitin (	Protein Co	46	GC22P021	3.88
MIR122	MicroRNA	RNA Gene	21	GC18P058	3.87
EDNRB	Endothelir	Protein Co	50	GC13M077	3.85
HOXA13	Homeobo:	Protein Co	43	GC07M027	3.85
FZD4	Frizzled Cl	Protein Co	52	GC11M086	3.82
HDAC1	Histone De	Protein Co	51	GC01P032	3.82
CASP2	Caspase 2	Protein Co	50	GC07P144	3.82
RRM2	Ribonucle	Protein Co	50	GC02P010	3.82
IRS1	Insulin Rec	Protein Co	49	GC02M226	3.82
P2RY12	Purinergic	Protein Co	49	GC03M151	3.82
PPP2R1B	Protein Ph	Protein Co	49	GC11M111	3.82
DIABLO	Diablo IAP	Protein Co	49	GC12M122	3.82
CACNA1G	Calcium V	Protein Co	49	GC17P050	3.82
PLK4	Polo Like I	Protein Co	48	GC04P127	3.82
SOX5	SRY-Box T	Protein Co	48	GC12M023	3.82
FOLH1	Folate Hyc	Protein Co	48	GC11M059	3.82
FZD6	Frizzled Cl	Protein Co	48	GC08P103	3.82
ADORA1	Adenosine	Protein Co	48	GC01P203	3.82
LRP1	LDL Recep	Protein Co	48	GC12P057	3.82
IGF2R	Insulin Like	Protein Co	47	GC06P159	3.82

HTR7	5-Hydroxy	Protein Co	47	GC10M09(	3.82
RALA	RAS Like P	Protein Co	47	GC07P039	3.82
PXN	Paxillin	Protein Co	47	GC12M12(	3.82
PSMB7	Proteasom	Protein Co	47	GC09M12	3.82
E2F4	E2F Transc	Protein Co	47	GC16P067	3.82
SAT1	Spermidin	Protein Co	47	GC0XP023	3.82
MAP2K7	Mitogen-/	Protein Co	47	GC19P007	3.82
TCF3	Transcripti	Protein Co	47	GC19M00	3.82
WNT10A	Wnt Famil	Protein Co	47	GC02P218	3.82
TFDP1	Transcripti	Protein Co	46	GC13P113	3.82
TIAM1	TIAM Rac1	Protein Co	46	GC21M03	3.82
PIK3R5	Phosphoir	Protein Co	46	GC17M00	3.82
CPE	Carboxype	Protein Co	46	GC04P165	3.82
SEMA4A	Semaphor	Protein Co	46	GC01P156	3.82
ROS1	ROS Proto	Protein Co	46	GC06M11	3.82
MELK	Maternal E	Protein Co	46	GC09P036	3.82
EPHB6	EPH Recep	Protein Co	46	GC07P144	3.82
STK24	Serine/Thr	Protein Co	45	GC13M09	3.82
TCF7	Transcripti	Protein Co	45	GC05P134	3.82
FZD1	Frizzled Cl	Protein Co	45	GC07P091	3.82
FZD3	Frizzled Cl	Protein Co	45	GC08P028	3.82
FOXM1	Forkhead I	Protein Co	45	GC12M00	3.82
TPH1	Tryptopha	Protein Co	45	GC11M01	3.82
PDGFD	Platelet De	Protein Co	45	GC11M10	3.82
SLCO1B3	Solute Car	Protein Co	45	GC12P020	3.82
TCOF1	Treacle Rib	Protein Co	44	GC05P150	3.82
QKI	QKI, KH D	Protein Co	44	GC06P163	3.82
KLF5	Kruppel Lil	Protein Co	44	GC13P073	3.82
FZD10	Frizzled Cl	Protein Co	44	GC12P130	3.82
MBD4	Methyl-Cp	Protein Co	44	GC03M12	3.82
ACSL5	Acyl-CoA	Protein Co	44	GC10P112	3.82
BNIP3	BCL2 Inter	Protein Co	44	GC10M13	3.82
TRIM28	Tripartite I	Protein Co	44	GC19P058	3.82
LASP1	LIM And S	Protein Co	44	GC17P038	3.82
PTTG1	PTTG1 Reg	Protein Co	44	GC05P160	3.82
SLC16A7	Solute Car	Protein Co	43	GC12P059	3.82
RALGDS	Ral Guanir	Protein Co	43	GC09M13	3.82
TCF7L1	Transcripti	Protein Co	43	GC02P085	3.82
DCLK1	Doublecor	Protein Co	43	GC13M03	3.82
FPGS	Folylpolyg	Protein Co	43	GC09P127	3.82
LGR6	Leucine Ri	Protein Co	43	GC01P202	3.82
XRCC2	X-Ray Rep	Protein Co	43	GC07M15	3.82
PLD3	Phospholi	Protein Co	42	GC19P040	3.82
PHLPP2	PH Domai	Protein Co	42	GC16M07	3.82
PMAIP1	Phorbol-1	Protein Co	41	GC18P059	3.82
PMS1	PMS1 Hon	Protein Co	41	GC02P189	3.82
SIL1	SIL1 Nucle	Protein Co	41	GC05M13	3.82
KMT2C	Lysine Me	Protein Co	41	GC07M15	3.82
EIF5A2	Eukaryotic	Protein Co	41	GC03M17(	3.82
NFYB	Nuclear Tr	Protein Co	41	GC12M10	3.82
AGO2	Argonaute	Protein Co	41	GC08M14(	3.82
AKAP12	A-Kinase /	Protein Co	41	GC06P151	3.82
UHRF1	Ubiquitin L	Protein Co	41	GC19P004	3.82
USP28	Ubiquitin S	Protein Co	41	GC11M11	3.82
PHLPP1	PH Domai	Protein Co	41	GC18P062	3.82
SLC45A2	Solute Car	Protein Co	41	GC05M03	3.82
PZP	PZP Alpha	Protein Co	40	GC12M00	3.82
HLTF	Helicase Li	Protein Co	40	GC03M14	3.82

GPA33	Glycoprote	Protein Co	40	GC01M167	3.82
CDCP1	CUB Doma	Protein Co	40	GC03M045	3.82
LLGL1	LLGL Scrib	Protein Co	40	GC17P018	3.82
RNF43	Ring Finge	Protein Co	39	GC17M058	3.82
EVL	Enah/Vasp	Protein Co	39	GC14P099	3.82
FAT4	FAT Atypic	Protein Co	39	GC04P125	3.82
TNS4	Tensin 4	Protein Co	39	GC17M040	3.82
HAPLN3	Hyalurona	Protein Co	38	GC15M088	3.82
ZNF217	Zinc Finge	Protein Co	38	GC20M053	3.82
HTR3E	5-Hydroxy	Protein Co	37	GC03P184	3.82
MACC1	MET Trans	Protein Co	37	GC07M020	3.82
PSG2	Pregnancy	Protein Co	36	GC19M043	3.82
CSMD3	CUB And S	Protein Co	36	GC08M112	3.82
SEPTIN9	Septin 9	Protein Co	36	GC17P077	3.82
USP40	Ubiquitin S	Protein Co	36	GC02M233	3.82
CLDND1	Claudin Do	Protein Co	36	GC03M098	3.82
KRTDAP	Keratinocy	Protein Co	34	GC19M041	3.82
P3H3	Prolyl 3-H	Protein Co	33	GC12P007	3.82
VWA8	Von Willeb	Protein Co	32	GC13M041	3.82
MRGPRE	MAS Relat	Protein Co	30	GC11M003	3.82
KRTAP9-2	Keratin As	Protein Co	29	GC17P041	3.82
COLCA2	Colorectal	Protein Co	25	GC11P111	3.82
COLCA1	Colorectal	Protein Co	24	GC11M111	3.82
TP53TG1	TP53 Targ	RNA Gene	24	GC07M087	3.82
LINC00472	Long Inter	RNA Gene	24	GC06M071	3.82
MIR96	MicroRNA	RNA Gene	21	GC07M129	3.82
MIR128-2	MicroRNA	RNA Gene	21	GC03P035	3.82
FBXL19-AS	FBXL19 An	RNA Gene	21	GC16M030	3.82
MIR339	MicroRNA	RNA Gene	20	GC07M001	3.82
MIR181A2	MicroRNA	RNA Gene	20	GC09P124	3.82
MIR32	MicroRNA	RNA Gene	20	GC09M109	3.82
HOXB-AS3	HOXB Clus	RNA Gene	20	GC17P048	3.82
MRGPRG-	MRGPRG A	RNA Gene	20	GC11P003	3.82
MIR183	MicroRNA	RNA Gene	19	GC07M129	3.82
SNHG3	Small Nucl	RNA Gene	19	GC01P028	3.82
MIR328	MicroRNA	RNA Gene	19	GC16M067	3.82
SCARNA6	Small Caja	RNA Gene	19	GC02P233	3.82
MIR7-3	MicroRNA	RNA Gene	18	GC19P004	3.82
SNHG17	Small Nucl	RNA Gene	18	GC20M038	3.82
SNORA54	Small Nucl	RNA Gene	18	GC11M002	3.82
MIR497	MicroRNA	RNA Gene	18	GC17M007	3.82
BCYRN1	Brain Cyto	RNA Gene	18	GC02P047	3.82
FTX	FTX Transc	RNA Gene	18	GC0XM073	3.82
MIR372	MicroRNA	RNA Gene	17	GC19P054	3.82
MIR422A	MicroRNA	RNA Gene	17	GC15M063	3.82
SOX21-AS	SOX21 An	RNA Gene	17	GC13P094	3.82
MIR95	MicroRNA	RNA Gene	17	GC04M008	3.82
MNX1-AS	MNX1 Ant	RNA Gene	17	GC07P157	3.82
DLEU7-AS	DLEU7 Ant	RNA Gene	17	GC13P050	3.82
CAHM	Colon Ade	RNA Gene	17	GC06M163	3.82
FAM83H-A	FAM83H A	RNA Gene	17	GC08P143	3.82
LEF1-AS1	LEF1 Antis	RNA Gene	17	GC04P108	3.82
LINC-PINT	Long Inter	RNA Gene	17	GC07M130	3.82
POU5F1P4	POU Class	Pseudoger	16	GC01P155	3.82
MIR625	MicroRNA	RNA Gene	16	GC14P065	3.82
HIF1A-AS1	HIF1A Ant	RNA Gene	16	GC14M061	3.82
SBDSP1	SBDS Pseu	Pseudoger	16	GC07P072	3.82
CASC11	Cancer Su	RNA Gene	16	GC08M127	3.82

RNY1	RNA, Ro6(	RNA Gene	16	GC07M14[illegible]	3.82
CASC8	Cancer Su	RNA Gene	16	GC08M12[illegible]	3.82
MACROD2	MACROD2	RNA Gene	16	GC20M01[illegible]	3.82
VIM-AS1	VIM Antise	RNA Gene	16	GC10M01[illegible]	3.82
LINC0065[illegible]	Long Inter	RNA Gene	16	GC20M06[illegible]	3.82
LINC0156[illegible]	Long Inter	RNA Gene	16	GC16M02[illegible]	3.82
LINC0113[illegible]	Long Inter	RNA Gene	16	GC01P159	3.82
FOXP4-AS	FOXP4 An	RNA Gene	15	GC06M04[illegible]	3.82
DPP10-AS	DPP10 Ant	RNA Gene	15	GC02M11[illegible]	3.82
RNY3	RNA, Ro6(	RNA Gene	15	GC07P149	3.82
NORAD	Non-Codi	RNA Gene	15	GC20M03[illegible]	3.82
NNT-AS1	NNT Antis	RNA Gene	15	GC05M04[illegible]	3.82
SLC25A25	SLC25A25	RNA Gene	15	GC09M12[illegible]	3.82
HIPK1-AS[illegible]	HIPK1 Ant	RNA Gene	14	GC01M11[illegible]	3.82
DLGAP4-A	DLGAP4 A	RNA Gene	14	GC20M03[illegible]	3.82
GABPB1-A	GABPB1 A	RNA Gene	14	GC15P050	3.82
BACE1-AS	BACE1 Ant	RNA Gene	14	GC11P117	3.82
C10orf143	Chromoso	Protein Co	14	GC10M13[illegible]	3.82
CASC19	Cancer Su	RNA Gene	14	GC08M12[illegible]	3.82
LINC0085[illegible]	Long Inter	RNA Gene	14	GC10P084	3.82
ZNF582-A	ZNF582 Ar	RNA Gene	14	GC19P056	3.82
LINC0150[illegible]	Long Inter	RNA Gene	14	GC09P079	3.82
CLMAT3	Colorectal	RNA Gene	13	GC05P151	3.82
GSEC	G-Quadru	RNA Gene	13	GC11M12[illegible]	3.82
EHHADH-	EHHADH A	RNA Gene	13	GC03P185	3.82
MAMDC2-	MAMDC2	RNA Gene	13	GC09M07[illegible]	3.82
DUXAP10	Double Hc	Pseudoger	12	GC14M01[illegible]	3.82
RBM5-AS1	RBM5 Anti	RNA Gene	12	GC03M05[illegible]	3.82
PURPL	P53 Upreg	RNA Gene	12	GC05P027	3.82
MROCKI	MARCKS (	RNA Gene	12	GC06M11[illegible]	3.82
AOC4P	Amine Oxi	Pseudoger	12	GC17P042	3.82
LINC0053[illegible]	Long Inter	RNA Gene	12	GC01P213	3.82
WSPAR	WNT Signa	RNA Gene	12	GC05P133	3.82
TUSC8	Tumor Sup	RNA Gene	12	GC13M04[illegible]	3.82
PINCR	P53-Induc	RNA Gene	11	GC0XP043	3.82
NCF4-AS1	NCF4 Antis	RNA Gene	11	GC22M03[illegible]	3.82
LAMC1-AS	LAMC1 Ar	RNA Gene	11	GC01M18[illegible]	3.82
ENSG00000228082		RNA Gene	10	GC06P100	3.82
BTG3-AS1	BTG3 Antis	RNA Gene	10	GC21P017	3.82
LINC0222[illegible]	Long Inter	RNA Gene	10	GC05P017	3.82
LINC0244[illegible]	Long Inter	RNA Gene	10	GC12P010	3.82
LINC0208[illegible]	Long Inter	RNA Gene	10	GC17P048	3.82
LINC0161[illegible]	Long Inter	RNA Gene	10	GC08P073	3.82
ENSG00000237588		RNA Gene	9	GC01M15[illegible]	3.82
ENSG00000260196		RNA Gene	9	GC11P017	3.82
SEC63P1	SEC63 Hor	Pseudoger	8	GC01P097	3.82
ENSG00000257241		RNA Gene	8	GC12M06[illegible]	3.82
ENSG00000266680		RNA Gene	8	GC06M06[illegible]	3.82
DACOR1	DNMT1-A	RNA Gene	7	GC15U902	3.82
FGF7P5	Fibroblast	Pseudoger	6	GC09P043	3.82
IBD19	Inflammat	Genetic Lc	2	GC00U934	3.82
TNFSF13B	TNF Super	Protein Co	47	GC13P108	3.81
SCG2	Secretogra	Protein Co	40	GC02M22[illegible]	3.8
RPL39P28	Ribosomal	Pseudoger	8	GC12M06[illegible]	3.8
CYP21A2	Cytochron	Protein Co	46	GC06P033	3.8
SCYL1	SCY1 Like	Protein Co	43	GC11P065	3.78
PI3	Peptidase	Protein Co	41	GC20P045	3.78
F2RL3	F2R Like Tl	Protein Co	46	GC19P016	3.77

CHGB	Chromogr	Protein Co	41	GC20P005	3.75
INSL6	Insulin Like	Protein Co	36	GC09M004	3.75
REN	Renin	Protein Co	50	GC01M204	3.75
RPS14P1	Ribosomal	Pseudoger	6	GC01M206	3.74
ENO2	Enolase 2	Protein Co	49	GC12P006	3.73
PTGER3	Prostaglan	Protein Co	47	GC01M070	3.72
DNAH8	Dynein Ax	Protein Co	38	GC06P046	3.71
DEFB103B	Defensin E	Protein Co	31	GC08M007	3.71
TRA	T Cell Rece	Protein Co	17	GC14P021	3.7
GPD2	Glycerol-3	Protein Co	47	GC02P156	3.7
CYP3A5	Cytochron	Protein Co	48	GC07M099	3.69
RTEL1-TN	RTEL1-TN	RNA Gene	18	GC20P063	3.69
CCS	Copper Ch	Protein Co	44	GC11P066	3.68
ACTA2	Actin Alph	Protein Co	49	GC10M088	3.67
UCN2	Urocortin :	Protein Co	35	GC03M048	3.65
KLRK1	Killer Cell I	Protein Co	41	GC12M013	3.63
PRDM1	PR/SET Dc	Protein Co	47	GC06P105	3.63
C5AR1	Compleme	Protein Co	45	GC19P047	3.63
TNPO3	Transporti	Protein Co	40	GC07M128	3.62
DPF2	Double PH	Protein Co	43	GC11P065	3.62
TH	Tyrosine H	Protein Co	53	GC11M002	3.62
TYRP1	Tyrosinase	Protein Co	48	GC09P012	3.62
BIRC2	Baculovira	Protein Co	47	GC11P102	3.6
PTX3	Pentraxin :	Protein Co	43	GC03P157	3.59
CFP	Compleme	Protein Co	45	GC0XM047	3.59
DAXX	Death Dor	Protein Co	45	GC06M033	3.58
LGALS9	Galectin 9	Protein Co	40	GC17P027	3.57
FBLIM1	Filamin Bir	Protein Co	39	GC01P015	3.57
CD19	CD19 Mole	Protein Co	50	GC16P028	3.56
TNFSF11	TNF Super	Protein Co	49	GC13P042	3.56
FCN2	Ficolin 2	Protein Co	41	GC09P134	3.56
FCN3	Ficolin 3	Protein Co	41	GC01M027	3.56
CFHR2	Compleme	Protein Co	37	GC01P196	3.56
KRT1	Keratin 1	Protein Co	45	GC12M052	3.56
GOT1	Glutamic-(	Protein Co	48	GC10M099	3.56
AICDA	Activation	Protein Co	47	GC12M008	3.55
VDAC1	Voltage De	Protein Co	47	GC05M133	3.55
CCHCR1	Coiled-Co	Protein Co	39	GC06M031	3.54
BVES	Blood Ves	Protein Co	41	GC06M105	3.53
JAZF1	JAZF Zinc	Protein Co	40	GC07M027	3.5
SERPINB1	Serpin Fan	Protein Co	41	GC06M002	3.49
FOXP2	Forkhead I	Protein Co	45	GC07P114	3.48
HLA-F-AS	HLA-F Ant	RNA Gene	18	GC06M030	3.47
ZGPAT	Zinc Finge	Protein Co	38	GC20P063	3.47
PARK7	Parkinsoni	Protein Co	47	GC01P007	3.47
HLA-DRB3	Major Hist	Protein Co	28	GC06Mn03	3.46
FAF1	Fas Associ	Protein Co	45	GC01M050	3.45
MUC13	Mucin 13,	Protein Co	37	GC03M124	3.45
KRT9	Keratin 9	Protein Co	41	GC17M041	3.45
APOE	Apolipopr	Protein Co	52	GC19P044	3.44
DEFB103A	Defensin E	Protein Co	29	GC08P007	3.43
IKZF3	IKAROS Fa	Protein Co	43	GC17M039	3.42
LSP1	Lymphocy	Protein Co	43	GC11P001	3.42
PLCH2	Phospholip	Protein Co	40	GC01P002	3.42
DAP	Death Ass	Protein Co	39	GC05M010	3.42
TNFSF8	TNF Super	Protein Co	38	GC09M114	3.42
SLC2A4RG	SLC2A4 Re	Protein Co	37	GC20P063	3.42
MMEL1	Membrane	Protein Co	36	GC01M002	3.42

NXPE1	Neurexopl	Protein Co	30	GC11M114	3.42
ENTR1	Endosome	Protein Co	29	GC09M136	3.42
C5orf66	Chromoso	RNA Gene	21	GC05P135	3.42
LINC00598	Long Inter	RNA Gene	15	GC13M040	3.42
WASL	WASP Like	Protein Co	43	GC07M123	3.41
RPN2	Ribophorin	Protein Co	43	GC20P037	3.39
DCT	Dopachror	Protein Co	42	GC13M094	3.39
CSN1S1	Casein Alp	Protein Co	34	GC04P069	3.39
TNFSF12	TNF Super	Protein Co	42	GC17P007	3.38
STK39	Serine/Thr	Protein Co	45	GC02M167	3.38
PLA2G7	Phospholip	Protein Co	51	GC06M046	3.38
LIF	LIF Interleu	Protein Co	44	GC22M030	3.37
SEC16A	SEC16 Hor	Protein Co	38	GC09M136	3.37
S100B	S100 Calci	Protein Co	47	GC21M047	3.37
PTGES	Prostaglan	Protein Co	42	GC09M129	3.35
IL13RA1	Interleukin	Protein Co	44	GC0XP118	3.35
SATB2	SATB Hom	Protein Co	45	GC02M199	3.34
TPPP	Tubulin Po	Protein Co	41	GC05M000	3.34
CEP72	Centrosom	Protein Co	37	GC05P000	3.34
FCGR2C	Fc Fragme	Protein Co	35	GC01P161	3.34
MROH3P	Maestro H	Pseudogen	12	GC01P200	3.34
HAMP	Hepcidin A	Protein Co	44	GC19P037	3.34
CD3G	CD3g Mol	Protein Co	48	GC11P118	3.33
CD151	CD151 Mo	Protein Co	47	GC11P000	3.33
GCH1	GTP Cyclo	Protein Co	47	GC14M054	3.33
METAP2	Methionyl	Protein Co	44	GC12P095	3.33
ALG3	ALG3 Alph	Protein Co	43	GC03M184	3.33
SSR2	Signal Seq	Protein Co	41	GC01M156	3.33
TRDMT1	TRNA Asp	Protein Co	41	GC10M017	3.33
CTDSPL	CTD Small	Protein Co	40	GC03P037	3.33
PSMC3IP	PSMC3 Int	Protein Co	40	GC17M042	3.33
NEK5	NIMA Rela	Protein Co	36	GC13M052	3.33
IDNK	IDNK Gluc	Protein Co	35	GC09P083	3.33
IGLL5	Immunogl	Protein Co	30	GC22P023	3.33
MT-RNR2	Mitochonc	RNA Gene	20	GCMTP001	3.33
HSD11B2	Hydroxyste	Protein Co	47	GC16P067	3.32
SERPINA1	Serpin Fan	Protein Co	51	GC14M094	3.32
USP4	Ubiquitin S	Protein Co	43	GC03M049	3.31
KCP	Kielin Cyst	Protein Co	33	GC07M128	3.3
AFP	Alpha Fetc	Protein Co	47	GC04P073	3.29
JUP	Junction P	Protein Co	48	GC17M041	3.29
TAGLN	Transgelin	Protein Co	43	GC11P117	3.29
MSH5	MutS Hom	Protein Co	42	GC06P033	3.27
NOS1	Nitric Oxic	Protein Co	50	GC12M117	3.26
APEH	Acylamino	Protein Co	41	GC03P049	3.26
MDK	Midkine	Protein Co	45	GC11P046	3.25
KIR3DL1	Killer Cell I	Protein Co	41	GC19P055	3.25
C5	Compleme	Protein Co	48	GC09M120	3.25
PFKFB4	6-Phosphc	Protein Co	43	GC03M048	3.25
IP6K2	Inositol He	Protein Co	41	GC03M048	3.25
IP6K1	Inositol He	Protein Co	40	GC03M049	3.25
SBNO2	Strawberry	Protein Co	33	GC19M001	3.25
GZMA	Granzyme	Protein Co	43	GC05P055	3.25
COL1A1	Collagen T	Protein Co	52	GC17M050	3.23
NTF3	Neurotrop	Protein Co	45	GC12P005	3.23
LYZ	Lysozyme	Protein Co	49	GC12P069	3.22
HSPA1A	Heat Shoc	Protein Co	45	GC06P033	3.22
TRAF2	TNF Recep	Protein Co	46	GC09P136	3.22

GJA1	Gap Juncti	Protein Co	52	GC06P121	3.22
G6PD	Glucose-6	Protein Co	52	GC0XM154	3.22
PRG2	Proteoglyc	Protein Co	41	GC11M057	3.22
RETNLB	Resistin Lik	Protein Co	36	GC03M108	3.21
PSORS1C1	Psoriasis S	Protein Co	29	GC06P031	3.21
ANPEP	Alanyl Am	Protein Co	50	GC15M089	3.21
MIR16-1	MicroRNA	RNA Gene	22	GC13M050	3.2
TARDBP	TAR DNA	Protein Co	47	GC01P011	3.19
CTSC	Cathepsin	Protein Co	46	GC11M088	3.19
TCN1	Transcoba	Protein Co	41	GC11M060	3.19
ARMH3	Armadillo	Protein Co	24	GC10M101	3.19
MIR135A1	MicroRNA	RNA Gene	21	GC03M052	3.19
MIR378A	MicroRNA	RNA Gene	20	GC05P149	3.19
MIR103A2	MicroRNA	RNA Gene	20	GC20P003	3.19
MIR582	MicroRNA	RNA Gene	18	GC05M059	3.19
MIR490	MicroRNA	RNA Gene	17	GC07P136	3.19
RCL1	RNA Term	Protein Co	38	GC09P004	3.18
HERC2	HECT And	Protein Co	45	GC15M028	3.18
FLOT1	Flotillin 1	Protein Co	43	GC06M030	3.17
TRIM39	Tripartite M	Protein Co	39	GC06P033	3.17
MYB	MYB Protc	Protein Co	51	GC06P135	3.17
HDGF	Heparin Bi	Protein Co	42	GC01M156	3.17
NLRP7	NLR Family	Protein Co	43	GC19M054	3.16
TXK	TXK Tyrosi	Protein Co	47	GC04M048	3.16
KIR2DL1	Killer Cell I	Protein Co	37	GC19P055	3.16
GAL	Galanin Ar	Protein Co	45	GC11P068	3.16
USP12	Ubiquitin S	Protein Co	37	GC13M027	3.16
INSL4	Insulin Like	Protein Co	33	GC09P005	3.16
TBXT	T-Box Trar	Protein Co	33	GC06M166	3.14
MIR196A2	MicroRNA	RNA Gene	22	GC12P054	3.13
DUOXA2	Dual Oxida	Protein Co	36	GC15P045	3.13
GPR65	G Protein-	Protein Co	40	GC14P088	3.13
C1orf141	Chromoso	Protein Co	28	GC01M067	3.11
IL7	Interleukin	Protein Co	44	GC08M078	3.11
PRDX1	Peroxiredc	Protein Co	51	GC01M045	3.11
P2RX3	Purinergic	Protein Co	42	GC11P057	3.11
PADI4	Peptidyl A	Protein Co	45	GC01P017	3.11
CYBB	Cytochron	Protein Co	48	GC0XP037	3.11
PNMT	Phenyletha	Protein Co	45	GC17P039	3.1
GMPPB	GDP-Man	Protein Co	44	GC03M049	3.1
NKX3-1	NK3 Home	Protein Co	43	GC08M023	3.1
UBA7	Ubiquitin L	Protein Co	43	GC03M049	3.1
UTS2	Urotensin	Protein Co	42	GC01M007	3.1
PIM3	Pim-3 Pro	Protein Co	40	GC22P049	3.1
SERINC3	Serine Inc	Protein Co	39	GC20M044	3.1
STMN3	Stathmin 3	Protein Co	38	GC20M063	3.1
EXOC3	Exocyst Cc	Protein Co	38	GC05P000	3.1
SNAPC4	Small Nucl	Protein Co	37	GC09M136	3.1
OLIG3	Oligodenc	Protein Co	36	GC06M137	3.1
AAMP	Angio Ass	Protein Co	36	GC02M218	3.1
ZBTB46	Zinc Finge	Protein Co	36	GC20M063	3.1
AMIGO3	Adhesion I	Protein Co	32	GC03M049	3.1
PRXL2B	Peroxiredc	Protein Co	27	GC01P002	3.1
LOC10099	Uncharact	RNA Gene	10	GC01P002	3.1
GLP2R	Glucagon	Protein Co	45	GC17P009	3.1
MME	Membrane	Protein Co	52	GC03P155	3.09
RNASEH2C	Ribonuclea	Protein Co	40	GC11M065	3.09
CCL17	C-C Motif	Protein Co	40	GC16P057	3.07

GPSM3	G Protein !	Protein Co	34	GC06M032	3.07
PBX2	PBX Home	Protein Co	39	GC06M032	3.06
CYP4F3	Cytochron	Protein Co	43	GC19P015	3.06
GPR12	G Protein-	Protein Co	39	GC13M02(	3.04
LBR	Lamin B Re	Protein Co	49	GC01M225	3.03
PHB	Prohibitin	Protein Co	49	GC17M049	3.02
IER3	Immediate	Protein Co	39	GC06M03(	3.02
CREM	CAMP Res	Protein Co	43	GC10P035	3.01
SDC1	Syndecan	Protein Co	45	GC02M02(	3.01
REG1A	Regenerat	Protein Co	43	GC02P079	3.01
PRPF8	Pre-MRNA	Protein Co	42	GC17M001	3
COG6	Componer	Protein Co	38	GC13P039	3
HNMT	Histamine	Protein Co	44	GC02P137	2.99
MIR210	MicroRNA	RNA Gene	21	GC11M00(	2.99
ADCY10	Adenylate	Protein Co	45	GC01M167	2.99
NGFR	Nerve Gro	Protein Co	47	GC17P049	2.98
CLIC1	Chloride Ir	Protein Co	43	GC06M032	2.98
IL15RA	Interleukin	Protein Co	43	GC10M005	2.96
LINC00243	Long Inter	RNA Gene	16	GC06M03(	2.96
HMGB2	High Mob	Protein Co	44	GC04M173	2.96
WASHC4	WASH Cor	Protein Co	31	GC12P105	2.95
STING1	Stimulator	Protein Co	35	GC05M139	2.95
ASGR1	Asialoglyc	Protein Co	41	GC17M007	2.94
IKBKB	Inhibitor C	Protein Co	54	GC08P042	2.94
CLDN3	Claudin 3	Protein Co	41	GC07M073	2.94
RNASE2	Ribonuclea	Protein Co	39	GC14P021	2.93
PERP	P53 Apopt	Protein Co	43	GC06M138	2.93
GSN	Gelsolin	Protein Co	50	GC09P121	2.93
LPL	Lipoprotei	Protein Co	51	GC08P019	2.93
NAGLU	N-Acetyl-,	Protein Co	44	GC17P042	2.92
IFNAR1	Interferon	Protein Co	47	GC21P033	2.92
TUBB6	Tubulin Be	Protein Co	43	GC18P012	2.91
CD244	CD244 Mc	Protein Co	44	GC01M16(	2.91
PTPRS	Protein Ty	Protein Co	44	GC19M005	2.9
SULT1A3	Sulfotrans	Protein Co	39	GC16P030	2.9
S1PR1	Sphingosir	Protein Co	46	GC01P101	2.9
SLC22A23	Solute Car	Protein Co	37	GC06M003	2.9
SFTPD	Surfactant	Protein Co	45	GC10M079	2.88
MAGI1	Membrane	Protein Co	41	GC03M065	2.88
MIR499A	MicroRNA	RNA Gene	22	GC20P034	2.88
DDR1	Discoidin I	Protein Co	48	GC06P033	2.86
CACNA2D	Calcium V	Protein Co	47	GC07M081	2.86
VARS2	Valyl-TRN	Protein Co	44	GC06P033	2.86
COL13A1	Collagen T	Protein Co	42	GC10P069	2.86
GCKR	Glucokinas	Protein Co	42	GC02P027	2.86
GTF2H4	General Tr	Protein Co	41	GC06P033	2.86
ACTR3B	Actin Relat	Protein Co	41	GC07P152	2.86
CPXM2	Carboxype	Protein Co	37	GC10M123	2.86
PRRC2A	Proline Ric	Protein Co	34	GC06P033	2.86
KPNA7	Karyopher	Protein Co	33	GC07M099	2.86
CAVIN1	Caveolae /	Protein Co	33	GC17M042	2.86
CALHM6	Calcium H	Protein Co	25	GC06P116	2.86
MIR26B	MicroRNA	RNA Gene	22	GC02P218	2.86
HCG9	HLA Comp	RNA Gene	21	GC06P033	2.86
HCG26	HLA Comp	RNA Gene	14	GC06P033	2.86
MIR588	MicroRNA	RNA Gene	14	GC06P126	2.86
CCR2	C-C Motif	Protein Co	47	GC03P046	2.86
THADA	THADA Ar	Protein Co	38	GC02M043	2.85

IL1RAP	Interleukin	Protein Co	45	GC03P190	2.85
RARRES2	Retinoic A	Protein Co	41	GC07M15(	2.85
BDKRB2	Bradykinin	Protein Co	45	GC14P096	2.84
ABL1	ABL Proto-	Protein Co	54	GC09P130	2.84
TERF2	Telomeric	Protein Co	41	GC16M069	2.82
CCL22	C-C Motif	Protein Co	39	GC16P057	2.81
OSCAR	Osteoclast	Protein Co	39	GC19M054	2.81
PAK1	P21 (RAC1	Protein Co	49	GC11M077	2.81
CD1A	CD1a Mole	Protein Co	43	GC01P158	2.8
TEP1	Telomeras	Protein Co	41	GC14M02(	2.8
CLU	Clusterin	Protein Co	48	GC08M027	2.79
FPR1	Formyl Pep	Protein Co	49	GC19M051	2.78
SULT1A2	Sulfotransf	Protein Co	43	GC16M028	2.77
NR5A2	Nuclear Re	Protein Co	47	GC01P199	2.77
ALDH1A1	Aldehyde	Protein Co	48	GC09M072	2.77
CXCL11	C-X-C Mo	Protein Co	41	GC04M076	2.76
ACHE	Acetylchol	Protein Co	47	GC07M10(	2.75
IKBKG	Inhibitor C	Protein Co	50	GC0XP154	2.73
ADORA2A	Adenosine	Protein Co	46	GC22P024	2.73
S100A1	S100 Calci	Protein Co	43	GC01P153	2.73
MFGE8	Milk Fat Gl	Protein Co	45	GC15M088	2.72
CBL	Cbl Proto-	Protein Co	52	GC11P119	2.71
TMPO	Thymopoi	Protein Co	45	GC12P098	2.71
GSDMA	Gasdermin	Protein Co	37	GC17P039	2.71
HPS3	HPS3 Biog	Protein Co	40	GC03P149	2.71
MTM1	Myotubula	Protein Co	45	GC0XP150	2.7
FTCD	Formimido	Protein Co	44	GC21M047	2.7
ASPH	Aspartate	Protein Co	44	GC08M061	2.7
PPP2R2C	Protein Ph	Protein Co	43	GC04M006	2.7
PPM1K	Protein Ph	Protein Co	43	GC04M088	2.7
MSLN	Mesothelin	Protein Co	42	GC16P001	2.7
MUC5B	Mucin 5B,	Protein Co	42	GC11P001	2.7
ANKH	ANKH Inor	Protein Co	41	GC05M014	2.7
ASGR2	Asialoglyco	Protein Co	41	GC17M007	2.7
GOLM1	Golgi Mem	Protein Co	40	GC09M086	2.7
SIGLEC8	Sialic Acid	Protein Co	39	GC19M051	2.7
PPFIBP2	PPFIA Binc	Protein Co	39	GC11P007	2.7
HOGA1	4-Hydroxy	Protein Co	39	GC10P097	2.7
AGBL2	ATP/GTP E	Protein Co	38	GC11M059	2.7
ZNF354A	Zinc Finge	Protein Co	38	GC05M178	2.7
TCFL5	Transcripti	Protein Co	37	GC20M062	2.7
PAM16	Presequen	Protein Co	37	GC16M004	2.7
LIN54	Lin-54 DRI	Protein Co	37	GC04M082	2.7
ISG20L2	Interferon	Protein Co	36	GC01M156	2.7
AGBL3	ATP/GTP E	Protein Co	36	GC07P134	2.7
CDH26	Cadherin 2	Protein Co	36	GC20P059	2.7
MUC15	Mucin 15,	Protein Co	35	GC11M026	2.7
USP50	Ubiquitin S	Protein Co	35	GC15M05(	2.7
MUCL1	Mucin Like	Protein Co	33	GC12P054	2.7
C6orf62	Chromoso	Protein Co	33	GC06M024	2.7
MUC21	Mucin 21,	Protein Co	32	GC06P033	2.7
KAAG1	Kidney Ass	Protein Co	28	GC06P024	2.7
FRG2C	FSHD Regi	Protein Co	24	GC03P075	2.7
MIR4741	MicroRNA	RNA Gene	14	GC18P022	2.7
MT-TF	Mitochonc	RNA Gene	14	GCMTP00(	2.7
FCRL3	Fc Recepto	Protein Co	37	GC01M157	2.7
KIR2DL3	Killer Cell I	Protein Co	37	GC19P055	2.69
PRDX4	Peroxiredo	Protein Co	44	GC0XP023	2.69

ZMIZ1	Zinc Finge	Protein Co	40	GC10P079	2.68
RMI2	RecQ Med	Protein Co	35	GC16P011	2.67
TRB	T Cell Rece	Protein Co	17	GC07P144	2.66
FKBP5	FKBP Proly	Protein Co	47	GC06M041	2.66
PSD	Pleckstrin /	Protein Co	41	GC10M102	2.66
TACR2	Tachykinin	Protein Co	44	GC10M069	2.65
DAB2	DAB Adap	Protein Co	44	GC05M039	2.65
TPM1	Tropomyo	Protein Co	50	GC15P072	2.64
LBP	Lipopolysa	Protein Co	45	GC20P038	2.63
CISD1	CDGSH Irc	Protein Co	39	GC10P058	2.62
PTGDR2	Prostaglan	Protein Co	43	GC11M060	2.61
SMARCB1	SWI/SNF F	Protein Co	47	GC22P023	2.6
SLC3A2	Solute Car	Protein Co	43	GC11P062	2.59
IRF4	Interferon	Protein Co	45	GC06P000	2.58
RHOH	Ras Homo	Protein Co	44	GC04P040	2.58
ACP1	Acid Phos	Protein Co	45	GC02P000	2.57
ABCB8	ATP Bindir	Protein Co	44	GC07P151	2.57
FCGRT	Fc Fragme	Protein Co	41	GC19P049	2.57
CCL19	C-C Motif	Protein Co	43	GC09M034	2.57
POU5F1	POU Class	Protein Co	48	GC06M031	2.57
PSMA6	Proteasom	Protein Co	47	GC14P035	2.57
RUNX1	RUNX Fam	Protein Co	50	GC21M034	2.56
PPP1R14A	Protein Ph	Protein Co	42	GC19M038	2.56
MRC1	Mannose I	Protein Co	40	GC10P017	2.56
ITGB7	Integrin Su	Protein Co	45	GC12M053	2.56
SIGIRR	Single Ig A	Protein Co	41	GC11M000	2.56
TNFRSF8	TNF Recep	Protein Co	44	GC01P012	2.55
LALBA	Lactalbum	Protein Co	40	GC12M048	2.55
GART	Phosphori	Protein Co	43	GC21M033	2.54
CD34	CD34 Mol	Protein Co	45	GC01M207	2.54
SAA4	Serum Am	Protein Co	40	GC11M018	2.54
DRD2	Dopamine	Protein Co	52	GC11M113	2.53
IFNAR2	Interferon	Protein Co	49	GC21P033	2.53
FGF23	Fibroblast	Protein Co	47	GC12M004	2.52
HLA-T	Major Hist	Pseudoger	10	GC06P033	2.51
LST1	Leukocyte	Protein Co	36	GC06P033	2.5
EPN3	Epsin 3	Protein Co	37	GC17P050	2.49
AQP4	Aquaporin	Protein Co	45	GC18M026	2.49
AQP7	Aquaporin	Protein Co	45	GC09M033	2.49
MAGI2	Membrane	Protein Co	44	GC07M078	2.49
ADRA2A	Adrenocep	Protein Co	48	GC10P111	2.49
TRAF1	TNF Recep	Protein Co	44	GC09M120	2.48
FLNA	Filamin A	Protein Co	50	GC0XM154	2.48
CYP27B1	Cytochron	Protein Co	48	GC12M057	2.47
RAVER2	Ribonucle	Protein Co	35	GC01P064	2.47
CAVIN3	Caveolae /	Protein Co	32	GC11M006	2.47
PLIN2	Perilipin 2	Protein Co	44	GC09M019	2.47
HLA-DQA	Major Hist	Protein Co	37	GC06P032	2.47
PRKCB	Protein Kir	Protein Co	49	GC16P023	2.46
CNTF	Ciliary Neu	Protein Co	43	GC11P058	2.46
HPSE	Heparanas	Protein Co	46	GC04M083	2.46
G6PC	Glucose-6	Protein Co	44	GC17P042	2.46
ETS2	ETS Proto-	Protein Co	43	GC21P038	2.45
IL24	Interleukin	Protein Co	44	GC01P206	2.44
SYP	Synaptopł	Protein Co	45	GC0XM049	2.44
TRG	T Cell Rece	Protein Co	12	GC07M038	2.43
ANGPT2	Angiopoie	Protein Co	46	GC08M006	2.43
CAMK4	Calcium/C	Protein Co	47	GC05P111	2.43

RBP4	Retinol Bir	Protein Co	45	GC10M093	2.43
VIL1	Villin 1	Protein Co	40	GC02P218	2.43
FYN	FYN Proto	Protein Co	50	GC06M111	2.42
USF1	Upstream	Protein Co	44	GC01M161	2.42
MAML2	Mastermin	Protein Co	39	GC11M095	2.42
LIME1	Lck Interac	Protein Co	33	GC20P063	2.42
ARHGAP45	Rho GTPas	Protein Co	30	GC19P001	2.42
RBP3	Retinol Bir	Protein Co	41	GC10P047	2.42
HLA-DMA	Major Hist	Protein Co	41	GC06M032	2.41
TRPV4	Transient I	Protein Co	51	GC12M109	2.41
LTB	Lymphoto:	Protein Co	41	GC06M032	2.41
ALDOB	Aldolase, F	Protein Co	45	GC09M101	2.4
SGF29	SAGA Con	Protein Co	31	GC16P028	2.4
CA2	Carbonic A	Protein Co	53	GC08P085	2.39
DEFA6	Defensin A	Protein Co	37	GC08M006	2.38
FLG	Filaggrin	Protein Co	41	GC01M152	2.37
RNF128	Ring Finge	Protein Co	39	GC0XP106	2.37
SLC6A14	Solute Car	Protein Co	41	GC0XP116	2.36
KHDRBS3	KH RNA Bi	Protein Co	39	GC08P135	2.36
APOA4	Apolipopr	Protein Co	43	GC11M116	2.36
NBN	Nibrin	Protein Co	49	GC08M089	2.35
TMPRSS6	Transmem	Protein Co	45	GC22M037	2.35
ADCYAP1	Adenylate	Protein Co	42	GC18P000	2.33
TNFSF14	TNF Super	Protein Co	43	GC19M006	2.33
CCR7	C-C Motif	Protein Co	46	GC17M040	2.33
ANXA7	Annexin A	Protein Co	44	GC10M073	2.32
PMEL	Premelanc	Protein Co	39	GC12M055	2.32
CYP2J2	Cytochron	Protein Co	45	GC01M059	2.32
IL17RD	Interleukin	Protein Co	43	GC03M057	2.32
MCM2	Minichrom	Protein Co	48	GC03P127	2.31
BLVRB	Biliverdin I	Protein Co	41	GC19M040	2.3
ELF3	E74 Like E	Protein Co	40	GC01P202	2.29
LTBR	Lymphoto:	Protein Co	43	GC12P006	2.28
STX2	Syntaxin 2	Protein Co	40	GC12M130	2.28
CASP14	Caspase 14	Protein Co	46	GC19P015	2.27
TFAP2A	Transcripti	Protein Co	48	GC06M010	2.27
ACADS	Acyl-CoA	Protein Co	47	GC12P120	2.27
PARD3	Par-3 Fam	Protein Co	43	GC10M034	2.27
CXCL3	C-X-C Mo	Protein Co	41	GC04M074	2.27
PTGDS	Prostaglan	Protein Co	46	GC09P136	2.26
IGES	Immunogl	Genetic Lo	6	GC05U990	2.25
SMARCA4	SWI/SNF F	Protein Co	52	GC19P010	2.23
RNF5	Ring Finge	Protein Co	41	GC06P033	2.23
IFITM3	Interferon	Protein Co	43	GC11M000	2.23
HLA-DMB	Major Hist	Protein Co	43	GC06M032	2.23
HLA-DOB	Major Hist	Protein Co	42	GC06M032	2.23
DEFA1	Defensin A	Protein Co	40	GC08M006	2.23
KLKB1	Kallikrein E	Protein Co	49	GC04P186	2.22
CCN1	Cellular Cc	Protein Co	33	GC01P085	2.22
HLA-DOA	Major Hist	Protein Co	42	GC06M033	2.21
BCL3	BCL3 Tran:	Protein Co	42	GC19P044	2.21
POSTN	Periostin	Protein Co	45	GC13M037	2.21
PF4	Platelet Fa	Protein Co	43	GC04M073	2.2
TBXAS1	Thrombox	Protein Co	51	GC07P139	2.2
PPBP	Pro-Platel	Protein Co	45	GC04M073	2.2
SOD3	Superoxid	Protein Co	40	GC04P024	2.19
BCL2L12	BCL2 Like	Protein Co	39	GC19P049	2.19
LYRM4	LYR Motif	Protein Co	39	GC06M005	2.19

ADCY7	Adenylate	Protein Co	47	GC16P050	2.18
MTMR3	Myotubula	Protein Co	43	GC22P029	2.18
HORMAD2	HORMA D	Protein Co	36	GC22P030	2.18
STC1	Stanniocal	Protein Co	41	GC08M023	2.18
TICAM1	Toll Like R	Protein Co	46	GC19M004	2.17
MECP2	Methyl-Cp	Protein Co	47	GC0XM154	2.17
FEN1	Flap Struct	Protein Co	48	GC11P061	2.17
ADAR	Adenosine	Protein Co	46	GC01M154	2.16
MIR124-3	MicroRNA	RNA Gene	18	GC20P063	2.16
CXCR5	C-X-C Mo	Protein Co	43	GC11P118	2.16
UBC	Ubiquitin (	Protein Co	44	GC12M124	2.15
EIF2S1	Eukaryotic	Protein Co	46	GC14P067	2.14
PSMB10	Proteasom	Protein Co	44	GC16M067	2.13
IL36A	Interleukin	Protein Co	37	GC02P113	2.12
RARG	Retinoic A	Protein Co	48	GC12M053	2.12
PITX1	Paired Like	Protein Co	46	GC05M135	2.12
IL1F10	Interleukin	Protein Co	41	GC02P113	2.12
GABPA	GA Binding	Protein Co	39	GC21P025	2.12
MAFK	MAF BZIP	Protein Co	37	GC07P001	2.12
IL36B	Interleukin	Protein Co	36	GC02M113	2.12
RPL35P3	Ribosomal	Pseudogen	8	GC06M105	2.12
ITGA5	Integrin Su	Protein Co	50	GC12M054	2.08
CXCL13	C-X-C Mo	Protein Co	43	GC04P077	2.07
CEBPB	CCAAT En	Protein Co	45	GC20P050	2.07
C1GALT1C	C1GALT1 S	Protein Co	38	GC0XM120	2.07
ADAM10	ADAM Me	Protein Co	54	GC15M058	2.07
FFAR2	Free Fatty	Protein Co	41	GC19P037	2.07
SYVN1	Synoviolin	Protein Co	41	GC11M065	2.07
AGFG1	ArfGAP Wi	Protein Co	40	GC02P227	2.06
BLOC1S2	Biogenesis	Protein Co	36	GC10M100	2.06
DPEP1	Dipeptidas	Protein Co	43	GC16P089	2.06
NTSR1	Neurotens	Protein Co	44	GC20P062	2.05
HTRA1	HtrA Serin	Protein Co	44	GC10P122	2.05
CYSLTR1	Cysteinyl L	Protein Co	44	GC0XM078	2.05
RPS2P34	Ribosomal	Pseudogen	6	GC09P082	2.05
LOC44242	Putative U	Pseudogen	4	GC09M082	2.05
HSP90B1	Heat Shoc	Protein Co	47	GC12P103	2.05
MGAM	Maltase-G	Protein Co	43	GC07P144	2.05
MAP3K8	Mitogen-A	Protein Co	50	GC10P030	2.05
SLC16A1	Solute Car	Protein Co	50	GC01M112	2.03
EED	Embryonic	Protein Co	44	GC11P086	2.03
PHOX2B	Paired Like	Protein Co	44	GC04M041	2.03
MAPKAPK	MAPK Acti	Protein Co	50	GC01P206	2.03
PPP5C	Protein Ph	Protein Co	46	GC19P046	2.03
TRIB1	Tribbles Ps	Protein Co	39	GC08P125	2.03
TSPAN33	Tetraspani	Protein Co	37	GC07P129	2.03
CIDEB	Cell Death	Protein Co	37	GC14M024	2.03
CD5	CD5 Molec	Protein Co	43	GC11P061	2.03
CCRL2	C-C Motif	Protein Co	40	GC03P046	2.02
HCRTR1	Hypocretin	Protein Co	44	GC01P031	2.02
CCR4	C-C Motif	Protein Co	47	GC03P032	2.01
GABBR1	Gamma-A	Protein Co	48	GC06M029	2.01
C2	Compleme	Protein Co	45	GC06P031	2.01
ATRIP	ATR Intera	Protein Co	43	GC03P048	2.01
TNC	Tenascin C	Protein Co	49	GC09M115	2
SHC1	SHC Adap	Protein Co	47	GC01M154	2
KHDRBS1	KH RNA Bi	Protein Co	42	GC01P031	2
HSPB1	Heat Shoc	Protein Co	52	GC07P076	1.99

CA1	Carbonic A	Protein Co	48	GC08M085	1.98
GZMM	Granzyme	Protein Co	40	GC19P000	1.98
FOXE1	Forkhead I	Protein Co	44	GC09P097	1.98
LEPQTL1	Leptin, Ser	Genetic Lo	4	GC02U903	1.98
IFNB1	Interferon	Protein Co	43	GC09M021	1.98
MAD2L1	Mitotic Arr	Protein Co	45	GC04M120	1.98
NPHP1	Nephrocys	Protein Co	44	GC02M110	1.98
HSF2	Heat Shoc	Protein Co	44	GC06P122	1.98
TNFRSF10I	TNF Recep	Protein Co	44	GC08M023	1.98
PRKN	Parkin RBR	Protein Co	41	GC06M161	1.98
TNFRSF10	TNF Recep	Protein Co	41	GC08P023	1.98
NACA	Nascent Po	Protein Co	39	GC12M056	1.98
CSNK1A1L	Casein Kin	Protein Co	37	GC13M037	1.98
FKBP15	FKBP Proly	Protein Co	35	GC09M113	1.98
FAM215A	Family Wit	RNA Gene	24	GC17P043	1.98
BORCS8-N	BORCS8-N	Protein Co	24	GC19M019	1.98
OCTN3	Organic Ca	Protein Co	4	GC05U900	1.98
FOSL2	FOS Like 2	Protein Co	42	GC02P028	1.98
TNFSF18	TNF Super	Protein Co	40	GC01M173	1.98
TAGAP	T Cell Acti	Protein Co	39	GC06M159	1.98
RIPK3	Receptor I	Protein Co	44	GC14M024	1.97
TRIM21	Tripartite M	Protein Co	43	GC11M004	1.97
CLSTN2	Calsynteni	Protein Co	40	GC03P139	1.97
DDIT4	DNA Dama	Protein Co	45	GC10P072	1.97
CAV2	Caveolin 2	Protein Co	44	GC07P116	1.97
CAV3	Caveolin 3	Protein Co	44	GC03P008	1.97
LRG1	Leucine Ri	Protein Co	40	GC19M004	1.97
DEFB104A	Defensin B	Protein Co	34	GC08P007	1.97
FLVCR1	FLVCR Her	Protein Co	41	GC01P212	1.96
CD160	CD160 Mo	Protein Co	41	GC01P145	1.96
NCF2	Neutrophi	Protein Co	48	GC01M183	1.95
CPOX	Coproporp	Protein Co	44	GC03M098	1.95
LAG3	Lymphocy	Protein Co	39	GC12P006	1.93
ALDH2	Aldehyde	Protein Co	52	GC12P111	1.93
ACAT2	Acetyl-Co	Protein Co	46	GC06P159	1.93
LGALS2	Galectin 2	Protein Co	42	GC22M037	1.93
TXNIP	Thioredoxi	Protein Co	39	GC01M145	1.93
MIR206	MicroRNA	RNA Gene	21	GC06P052	1.93
KCNQ1	Potassium	Protein Co	51	GC11P002	1.93
CBS	Cystathion	Protein Co	50	GC21M043	1.93
APAF1	Apoptotic	Protein Co	48	GC12P098	1.93
ATF2	Activating	Protein Co	47	GC02M175	1.93
TRAP1	TNF Recep	Protein Co	43	GC16M003	1.93
NTAN1	N-Termina	Protein Co	38	GC16M015	1.93
KIR2DS2	Killer Cell I	Protein Co	25	GC19MR0	1.93
KIR2DL2	Killer Cell I	Protein Co	24	GC19MP0	1.93
CD48	CD48 Mol	Protein Co	40	GC01M160	1.92
GIMAP5	GTPase, IM	Protein Co	38	GC07P150	1.92
CD1D	CD1d Mol	Protein Co	44	GC01P158	1.91
SLPI	Secretory I	Protein Co	41	GC20M045	1.91
ANKRD55	Ankyrin Re	Protein Co	33	GC05M056	1.91
INTS11	Integrator	Protein Co	29	GC01M001	1.91
AHSG	Alpha 2-H	Protein Co	45	GC03P186	1.89
DSG1	Desmoglei	Protein Co	45	GC18P031	1.89
TNFAIP6	TNF Alpha	Protein Co	42	GC02P151	1.89
SCNN1B	Sodium Ch	Protein Co	50	GC16P023	1.88
RRM2B	Ribonucle	Protein Co	50	GC08M102	1.88
SDHA	Succinate	Protein Co	48	GC05P000	1.88

HSD17B10	Hydroxyst	Protein Co	47	GC0XM053	1.88
HMGA1	High Mobi	Protein Co	47	GC06P046	1.88
SCNN1G	Sodium Ch	Protein Co	47	GC16P023	1.88
TFPI	Tissue Fac	Protein Co	46	GC02M187	1.88
SERPINF1	Serpin Fan	Protein Co	46	GC17P001	1.88
PTPRO	Protein Ty	Protein Co	46	GC12P015	1.88
CYC1	Cytochrom	Protein Co	46	GC08P144	1.88
KCNE3	Potassium	Protein Co	45	GC11M074	1.88
SLC18A3	Solute Car	Protein Co	45	GC10P049	1.88
CITED2	Cbp/P300	Protein Co	45	GC06M139	1.88
LMAN1	Lectin, Ma	Protein Co	45	GC18M059	1.88
GTF2E2	General Tr	Protein Co	44	GC08M030	1.88
RHOB	Ras Homo	Protein Co	44	GC02P020	1.88
AQP9	Aquaporin	Protein Co	44	GC15P058	1.88
PDCD1LG2	Programm	Protein Co	43	GC09P005	1.88
IFITM1	Interferon	Protein Co	43	GC11P000	1.88
MXI1	MAX Intera	Protein Co	43	GC10P110	1.88
EYA4	EYA Transc	Protein Co	43	GC06P133	1.88
TUSC3	Tumor Sup	Protein Co	43	GC08P015	1.88
UQCRQ	Ubiquinol-	Protein Co	43	GC05P132	1.88
TTBK2	Tau Tubuli	Protein Co	43	GC15M042	1.88
NMB	Neuromec	Protein Co	42	GC15M084	1.88
GPR55	G Protein-	Protein Co	41	GC02M230	1.88
GAS7	Growth Ar	Protein Co	41	GC17M009	1.88
SERPINA12	Serpin Fan	Protein Co	40	GC14M094	1.88
NEK6	NIMA Rela	Protein Co	40	GC09P124	1.88
MGAT5	Alpha-1,6-	Protein Co	40	GC02P134	1.88
ACSM3	Acyl-CoA	Protein Co	40	GC16P020	1.88
SMOX	Spermine (	Protein Co	40	GC20P004	1.88
MARVELD3	MARVEL D	Protein Co	40	GC05P069	1.88
CCL16	C-C Motif	Protein Co	39	GC17M035	1.88
APBA1	Amyloid B	Protein Co	39	GC09M069	1.88
CADM2	Cell Adhes	Protein Co	38	GC03P085	1.88
NUCKS1	Nuclear Ca	Protein Co	37	GC01M205	1.88
RC3H1	Ring Finge	Protein Co	36	GC01M173	1.88
BAHD1	Bromo Ad	Protein Co	36	GC15P040	1.88
ANP32E	Acidic Nuc	Protein Co	35	GC01M150	1.88
TAC4	Tachykinin	Protein Co	33	GC17M049	1.88
MT-ND4	Mitochond	Protein Co	33	GCMTP010	1.88
S100Z	S100 Calci	Protein Co	33	GC05P076	1.88
IGHG3	Immunogl	Protein Co	27	GC14M105	1.88
EXOC3-AS	EXOC3 An	RNA Gene	25	GC05M000	1.88
PRAC2	PRAC2 Sm	Protein Co	21	GC17P048	1.88
MIR4728	MicroRNA	RNA Gene	12	GC17P039	1.88
LOC11059	MS1 Minis	Biological	2	GC01U905	1.88
KIR3DL2	Killer Cell I	Protein Co	39	GC19P055	1.88
PDGFA	Platelet De	Protein Co	45	GC07M000	1.87
KSR1	Kinase Sup	Protein Co	42	GC17P027	1.87
MPG	N-Methylp	Protein Co	43	GC16P001	1.87
GALC	Galactosyl	Protein Co	44	GC14M087	1.87
CTSW	Cathepsin	Protein Co	41	GC11P065	1.87
CRTC1	CREB Regu	Protein Co	44	GC19P022	1.86
IL22RA2	Interleukin	Protein Co	40	GC06M137	1.86
NR4A1	Nuclear Re	Protein Co	48	GC12P052	1.85
AZU1	Azurocidin	Protein Co	40	GC19P000	1.85
SGK1	Serum/Glu	Protein Co	50	GC06M134	1.85
VNN1	Vanin 1	Protein Co	45	GC06M132	1.84
CXCL6	C-X-C Mo	Protein Co	41	GC04P073	1.84

ITGB3	Integrin Su	Protein Co	51	GC17P047	1.84
CTSL	Cathepsin	Protein Co	48	GC09P087	1.84
PTGIR	Prostaglan	Protein Co	48	GC19M046	1.83
GPX4	Glutathion	Protein Co	48	GC19P001	1.83
ADCY3	Adenylate	Protein Co	48	GC02M02	1.83
CARD11	Caspase R	Protein Co	48	GC07M00	1.83
ATF4	Activating	Protein Co	48	GC22P039	1.83
RIT1	Ras Like W	Protein Co	47	GC01M15	1.83
HDAC7	Histone De	Protein Co	47	GC12M04	1.83
RPS6KA4	Ribosomal	Protein Co	47	GC11P064	1.83
LNPEP	Leucyl Anc	Protein Co	46	GC05P096	1.83
TNNI2	Troponin I	Protein Co	46	GC11P001	1.83
REV3L	REV3 Like,	Protein Co	45	GC06M11	1.83
RGS14	Regulator	Protein Co	45	GC05P177	1.83
PMPCA	Peptidase,	Protein Co	44	GC09P136	1.83
ITIH4	Inter-Alph	Protein Co	44	GC03M05	1.83
CD226	CD226 Mc	Protein Co	44	GC18M06	1.83
FADS2	Fatty Acid	Protein Co	44	GC11P061	1.83
PNKD	PNKD Met	Protein Co	43	GC02P218	1.83
ITPKA	Inositol-Tr	Protein Co	43	GC15P041	1.83
FIBP	FGF1 Intra	Protein Co	43	GC11M06	1.83
CALM3	Calmoduli	Protein Co	43	GC19P046	1.83
NDUFAF1	NADH:Ubi	Protein Co	42	GC15M04	1.83
MANBA	Mannosida	Protein Co	42	GC04M10	1.83
BRD7	Bromodon	Protein Co	42	GC16M05	1.83
RASSF5	Ras Associ	Protein Co	41	GC01P206	1.83
SKAP2	Src Kinase	Protein Co	41	GC07M02	1.83
CTDSP1	CTD Small	Protein Co	41	GC02P218	1.83
CEP250	Centrosom	Protein Co	41	GC20P035	1.83
LPXN	Leupaxin	Protein Co	41	GC11M05	1.83
DUSP16	Dual Speci	Protein Co	41	GC12M01	1.83
CD6	CD6 Mole	Protein Co	41	GC11P060	1.83
ACSL6	Acyl-CoA	Protein Co	41	GC05M13	1.83
PDLIM4	PDZ And L	Protein Co	40	GC05P132	1.83
SLC7A10	Solute Car	Protein Co	40	GC19M03	1.83
PLCL1	Phospholi	Protein Co	40	GC02P197	1.83
CRTC3	CREB Regu	Protein Co	40	GC15P090	1.83
MSTO1	Misato Mit	Protein Co	40	GC01P155	1.83
SEMA6D	Semaphor	Protein Co	40	GC15P047	1.83
GPR18	G Protein-	Protein Co	40	GC13M09	1.83
CHP1	Calcineuri	Protein Co	39	GC15P041	1.83
ZNF365	Zinc Finge	Protein Co	39	GC10P062	1.83
NDFIP1	Nedd4 Far	Protein Co	38	GC05P142	1.83
ATXN2L	Ataxin 2 Li	Protein Co	38	GC16P028	1.83
SLC39A11	Solute Car	Protein Co	38	GC17M07	1.83
CPEB4	Cytoplasm	Protein Co	37	GC05P173	1.83
SFMBT1	Scm Like V	Protein Co	37	GC03M05	1.83
DOK3	Docking P	Protein Co	37	GC05M17	1.83
FCRLA	Fc Recepto	Protein Co	37	GC01P161	1.83
TMBIM1	Transmem	Protein Co	37	GC02M21	1.83
ZNF300	Zinc Finge	Protein Co	37	GC05M15	1.83
SNX20	Sorting Ne	Protein Co	36	GC16M05	1.83
SNX32	Sorting Ne	Protein Co	36	GC11P065	1.83
ARHGAP3	Rho GTPas	Protein Co	36	GC01M16	1.83
TSPAN14	Tetraspani	Protein Co	36	GC10P083	1.83
PHTF1	Putative H	Protein Co	36	GC01M11	1.83
TMEM50B	Transmem	Protein Co	35	GC21M03	1.83
RFTN2	Raftlin Fan	Protein Co	35	GC02M19	1.83

NUSAP1	Nucleolar	Protein Co	35	GC15P041	1.83
TTYH3	Tweety Fa	Protein Co	35	GC07P002	1.83
CCDC85B	Coiled-Co	Protein Co	34	GC11P065	1.83
YDJC	YdjC Chito	Protein Co	34	GC22M02	1.83
JRKL	JRK Like	Protein Co	33	GC11P096	1.83
PUSL1	Pseudouri	Protein Co	33	GC01P001	1.83
CCDC116	Coiled-Co	Protein Co	33	GC22P023	1.83
ZNF831	Zinc Finge	Protein Co	33	GC20P059	1.83
NXPE4	Neurexopl	Protein Co	31	GC11M11	1.83
C10orf55	Chromoso	RNA Gene	29	GC10M07	1.83
AHSA2P	Activator (	Pseudoger	24	GC02P061	1.83
TRAF3IP2-	TRAF3IP2	RNA Gene	18	GC06P111	1.83
IRF1-AS1	IRF1 Antis	RNA Gene	17	GC05P132	1.83
FLJ31356	Uncharact	RNA Gene	13	GC02M02	1.83
IRF3	Interferon	Protein Co	48	GC19M04	1.83
LAMP2	Lysosomal	Protein Co	46	GC0XM12	1.82
STX8	Syntaxin 8	Protein Co	40	GC17M00	1.82
NFKB2	Nuclear Fa	Protein Co	54	GC10P102	1.82
EPHB4	EPH Recep	Protein Co	52	GC07M10	1.82
SUFU	SUFU Neg	Protein Co	44	GC10P102	1.82
BANF1	BAF Nucle	Protein Co	43	GC11P066	1.82
CAPN10	Calpain 10	Protein Co	42	GC02P240	1.82
SLC35D1	Solute Car	Protein Co	41	GC01M06	1.82
ARFRP1	ADP Ribos	Protein Co	39	GC20M06	1.82
ZNF341	Zinc Finge	Protein Co	37	GC20P033	1.82
WDR6	WD Repea	Protein Co	36	GC03P049	1.82
MIR611	MicroRNA	RNA Gene	16	GC11M06	1.82
TEC	Tec Protei	Protein Co	47	GC04M04	1.82
KRT17	Keratin 17	Protein Co	47	GC17M04	1.81
ANG	Angiogeni	Protein Co	47	GC14P020	1.81
BLZF1	Basic Leuc	Protein Co	40	GC01P169	1.8
FOXP1	Forkhead	Protein Co	47	GC03M07	1.8
DNAL4	Dynein Ax	Protein Co	43	GC22M03	1.8
OSM	Oncostatir	Protein Co	44	GC22M03	1.79
DDX58	DExD/H-B	Protein Co	48	GC09M03	1.79
ADAM15	ADAM Me	Protein Co	41	GC01P155	1.78
RING1	Ring Finge	Protein Co	41	GC06P033	1.78
CYBC1	Cytochron	Protein Co	27	GC17M08	1.78
SUOX	Sulfite Oxi	Protein Co	46	GC12P055	1.77
FLNC	Filamin C	Protein Co	45	GC07P128	1.77
AIF1	Allograft I	Protein Co	41	GC06P033	1.77
ZNRD1	Zinc Ribbc	Protein Co	40	GC06P033	1.77
MX1	MX Dynan	Protein Co	42	GC21P041	1.77
VIPR1	Vasoactive	Protein Co	47	GC03P042	1.77
MIR130A	MicroRNA	RNA Gene	20	GC11P057	1.77
MIR429	MicroRNA	RNA Gene	20	GC01P001	1.76
CACNA1E	Calcium V	Protein Co	46	GC01P181	1.76
CD83	CD83 Mol	Protein Co	40	GC06P014	1.75
ARFGAP1	ADP Ribos	Protein Co	44	GC20P063	1.74
C4BPB	Compleme	Protein Co	41	GC01P207	1.74
SLC23A3	Solute Car	Protein Co	37	GC02M21	1.74
RO60	Ro60, Y RI	Protein Co	31	GC01P193	1.73
PER3	Period Circ	Protein Co	44	GC01P007	1.73
BDH2	3-Hydroxy	Protein Co	39	GC04M10	1.73
LRRC3C	Leucine Ri	Protein Co	26	GC17P039	1.73
GPR183	G Protein-	Protein Co	41	GC13M09	1.73
MEP1A	Meprin A	Protein Co	43	GC06P046	1.72
FOXO1	Forkhead	Protein Co	50	GC13M04	1.72

CASP4	Caspase 4	Protein Co	47	GC11M10	1.7
SYK	Spleen Ass	Protein Co	51	GC09P091	1.69
PEX5	Peroxisom	Protein Co	43	GC12P007	1.68
NFIL3	Nuclear Fa	Protein Co	41	GC09M09	1.68
GBGT1	Globoside	Protein Co	37	GC09M13	1.68
NEU1	Neuramini	Protein Co	45	GC06M03	1.67
CHRNA5	Cholinergi	Protein Co	45	GC15P078	1.66
CUX1	Cut Like H	Protein Co	44	GC07P101	1.66
KCNN1	Potassium	Protein Co	40	GC19P022	1.66
DDX39B	DExD-Box	Protein Co	40	GC06M03	1.65
ATP6V1G2	ATPase H	Protein Co	39	GC06M03	1.65
DEFA3	Defensin	Protein Co	37	GC08M00	1.65
SLC9A1	Solute Car	Protein Co	52	GC01M02	1.65
ST14	Suppressio	Protein Co	47	GC11P130	1.64
SPI1	Spi-1 Prot	Protein Co	45	GC11M05	1.64
PRSS8	Serine Pro	Protein Co	45	GC16M03	1.64
IL9R	Interleukin	Protein Co	36	GC0XP155	1.64
ATP6V0A1	ATPase H	Protein Co	43	GC17P042	1.64
SCD	Stearoyl-C	Protein Co	49	GC10P100	1.64
HADH	Hydroxyac	Protein Co	48	GC04P107	1.64
TERF2IP	TERF2 Inte	Protein Co	44	GC16P075	1.64
ATG7	Autophag	Protein Co	44	GC03P011	1.64
ACAP1	ArfGAP Wi	Protein Co	41	GC17P007	1.64
OVGP1	Oviductal	Protein Co	40	GC01M11	1.64
CHD4	Chromodo	Protein Co	46	GC12M00	1.63
CD38	CD38 Mol	Protein Co	46	GC04P015	1.63
VCL	Vinculin	Protein Co	49	GC10P073	1.63
CORO1A	Coronin 1	Protein Co	44	GC16P030	1.63
TRAF3	TNF Recep	Protein Co	48	GC14P104	1.63
IL17C	Interleukin	Protein Co	39	GC16P088	1.62
OSMR	Oncostatir	Protein Co	47	GC05P038	1.62
BDKRB1	Bradykinin	Protein Co	44	GC14P096	1.62
GP2	Glycoprote	Protein Co	40	GC16M02	1.61
ARRB2	Arrestin Be	Protein Co	45	GC17P004	1.59
FLI1	Fli-1 Proto	Protein Co	51	GC11P128	1.59
CD2	CD2 Mole	Protein Co	45	GC01P116	1.59
PTPN6	Protein Ty	Protein Co	51	GC12P007	1.58
TNFRSF17	TNF Recep	Protein Co	45	GC16P011	1.58
PLEK	Pleckstrin	Protein Co	41	GC02P068	1.58
SSRP1	Structure	Protein Co	41	GC11M05	1.58
FGL2	Fibrinoger	Protein Co	40	GC07M07	1.58
SUN2	Sad1 And	Protein Co	38	GC22M04	1.58
AKR1A1	Aldo-Keto	Protein Co	45	GC01P045	1.58
ETV5	ETS Varian	Protein Co	40	GC03M18	1.58
CD207	CD207 Mc	Protein Co	40	GC02M07	1.58
KEAP1	Kelch Like	Protein Co	48	GC19M01	1.57
RXRB	Retinoid X	Protein Co	48	GC06M03	1.57
CD81	CD81 Mol	Protein Co	48	GC11P002	1.57
SH2B3	SH2B Ada	Protein Co	47	GC12P111	1.57
GRB7	Growth Fa	Protein Co	45	GC17P039	1.57
ABCD4	ATP Bindir	Protein Co	44	GC14M07	1.57
DNMT3L	DNA Meth	Protein Co	43	GC21M04	1.57
CTDP1	CTD Phos	Protein Co	42	GC18P079	1.57
SLC39A7	Solute Car	Protein Co	42	GC06P033	1.57
HSD17B8	Hydroxyst	Protein Co	41	GC06P033	1.57
MYT1L	Myelin Tra	Protein Co	41	GC02M00	1.57
CDK12	Cyclin Dep	Protein Co	41	GC17P039	1.57
FNBP1	Formin Bir	Protein Co	40	GC09M12	1.57

IL22RA1	Interleukin	Protein Co	39	GC01M024	1.57
NCKIPSD	NCK Intera	Protein Co	37	GC03M048	1.57
NOP2	NOP2 Nuc	Protein Co	37	GC12M006	1.57
OR2H2	Olfactory I	Protein Co	36	GC06P033	1.57
SLC48A1	Solute Car	Protein Co	36	GC12P047	1.57
ENGASE	Endo-Beta	Protein Co	35	GC17P079	1.57
MPIG6B	Megakaryo	Protein Co	32	GC06P033	1.57
C3orf62	Chromoso	Protein Co	30	GC03M049	1.57
INS-IGF2	INS-IGF2 I	Protein Co	28	GC11M002	1.57
CCDC26	CCDC26 Lo	RNA Gene	27	GC08M128	1.57
PHETA1	PH Domai	Protein Co	27	GC12M111	1.57
SNHG7	Small Nucl	RNA Gene	21	GC09M136	1.57
MSTO2P	Misato Far	Pseudogen	14	GC01P155	1.57
FAS-AS1	FAS Antise	RNA Gene	14	GC10M088	1.57
TNK2	Tyrosine K	Protein Co	48	GC03M195	1.54
MLNR	Motilin Re	Protein Co	41	GC13P049	1.53
IRF7	Interferon	Protein Co	48	GC11M000	1.53
GAS6	Growth Ar	Protein Co	45	GC13M113	1.52
PFKM	Phosphofr	Protein Co	51	GC12P048	1.51
SLC23A1	Solute Car	Protein Co	44	GC05M139	1.51
SLC2A14	Solute Car	Protein Co	35	GC12M007	1.51
IL32	Interleukin	Protein Co	41	GC16P004	1.49
ARRB1	Arrestin Be	Protein Co	45	GC11M075	1.48
CAMK2G	Calcium/C	Protein Co	48	GC10M073	1.48
MACF1	Microtubu	Protein Co	41	GC01P039	1.47
GIP	Gastric Inh	Protein Co	40	GC17M048	1.46
HLA-DRB4	Major Hist	Protein Co	31	GC06Mo03	1.46
CYP2A6	Cytochrom	Protein Co	48	GC19M040	1.45
MCCD1	Mitochond	Protein Co	32	GC06P031	1.45
PROC	Protein C,	Protein Co	51	GC02P127	1.45
KCNMA1	Potassium	Protein Co	51	GC10M076	1.45
GHSR	Growth Ho	Protein Co	48	GC03M172	1.44
SLC39A8	Solute Car	Protein Co	44	GC04M102	1.44
APOBEC3C	Apolipopro	Protein Co	43	GC22P039	1.44
SLC39A12	Solute Car	Protein Co	39	GC10P017	1.44
DCLRE1B	DNA Cross	Protein Co	35	GC01P113	1.44
SELPLG	Selectin P	Protein Co	44	GC12M108	1.43
HLA-DQB1	Major Hist	Protein Co	37	GC06M032	1.43
PRRT1	Proline Ric	Protein Co	33	GC06M032	1.43
SNHG32	Small Nucl	RNA Gene	22	GC06P046	1.43
AOC3	Amine Oxi	Protein Co	45	GC17P042	1.43
MDC1	Mediator (	Protein Co	42	GC06M030	1.43
IGHM	Immunogl	Protein Co	32	GC14M105	1.42
SLC19A1	Solute Car	Protein Co	47	GC21M045	1.42
CD300LF	CD300 Mo	Protein Co	41	GC17M074	1.41
MYDGF	Myeloid D	Protein Co	35	GC19M004	1.41
C19orf33	Chromoso	Protein Co	31	GC19P038	1.41
RGN	Regucalcin	Protein Co	41	GC0XP047	1.4
SESN2	Sestrin 2	Protein Co	39	GC01P028	1.4
TIAL1	TIA1 Cytot	Protein Co	41	GC10M119	1.4
CSNK2B	Casein Kin	Protein Co	49	GC06P033	1.39
EHMT2	Euchromat	Protein Co	47	GC06M031	1.39
BRD2	Bromodom	Protein Co	45	GC06P033	1.39
LSM2	LSM2 Hom	Protein Co	41	GC06M032	1.39
B3GALT4	Beta-1,3-(	Protein Co	40	GC06P033	1.39
ATF6B	Activating	Protein Co	39	GC06M032	1.39
GPANK1	G-Patch D	Protein Co	33	GC06M032	1.39
HLA-DRB6	Major Hist	Pseudogen	17	GC06M032	1.39

CYP21A1P	Cytochron	Pseudoger	17	GC06P032	1.39
HLA-DQB:	HLA-DQB:	RNA Gene	12	GC06P032	1.39
ALOX15B	Arachidon	Protein Co	43	GC17P008	1.39
IGSF3	Immunogl	Protein Co	40	GC01M11(	1.39
CARD16	Caspase R	Protein Co	39	GC11M10!	1.39
TRD	T Cell Rece	Protein Co	13	GC14P022	1.39
MIR19B1	MicroRNA	RNA Gene	18	GC13P091	1.38
MAP3K11	Mitogen-A	Protein Co	49	GC11M06!	1.37
TRIM31	Tripartite M	Protein Co	39	GC06M03(	1.37
MLXIPL	MLX Intera	Protein Co	42	GC07M07:	1.37
TST	Thiosulfate	Protein Co	43	GC22M03	1.36
C1GALT1	Core 1 Syr	Protein Co	41	GC07P007	1.35
DUSP1	Dual Speci	Protein Co	48	GC05M17	1.35
CGN	Cingulin	Protein Co	40	GC01P151	1.34
HCG27	HLA Comp	RNA Gene	22	GC06P031	1.34
ENSG00000271581		Pseudoger	6	GC06P033	1.34
APOA1	Apolipopro	Protein Co	50	GC11M11(	1.34
OTC	Ornithine (	Protein Co	48	GC0XP038	1.34
UCP2	Uncouplin	Protein Co	47	GC11M07:	1.34
IL17D	Interleukin	Protein Co	39	GC13P020	1.34
SECTM1	Secreted A	Protein Co	39	GC17M08	1.34
MMP19	Matrix Me	Protein Co	48	GC12M05!	1.33
DSC2	Desmocoll	Protein Co	47	GC18M03	1.33
CMA1	Chymase 1	Protein Co	45	GC14M02	1.32
H2AC21	H2A Cluste	Protein Co	28	GC01M14!	1.32
CD84	CD84 Mole	Protein Co	43	GC01M16(	1.32
GDNF	Glial Cell D	Protein Co	49	GC05M03	1.31
SKI	SKI Proto-	Protein Co	46	GC01P002	1.31
SLC22A2	Solute Car	Protein Co	45	GC06M16(	1.31
SLC26A6	Solute Car	Protein Co	40	GC03M04{	1.31
CXCR6	C-X-C Mo	Protein Co	41	GC03P045	1.31
NFATC2	Nuclear Fa	Protein Co	47	GC20M05	1.3
MARCKS	Myristoyla	Protein Co	41	GC06P113	1.3
RXRA	Retinoid X	Protein Co	51	GC09P134	1.3
APLN	Apelin	Protein Co	39	GC0XM12!	1.28
AKR1B1	Aldo-Keto	Protein Co	49	GC07M13	1.28
CYTH1	Cytohesin	Protein Co	43	GC17M07{	1.27
FBXW11	F-Box Anc	Protein Co	43	GC05M17	1.27
RUNX2	RUNX Fam	Protein Co	48	GC06P045	1.27
EDN2	Endothelir	Protein Co	41	GC01M04	1.26
TNPO1	Transporti	Protein Co	40	GC05P072	1.25
MFSD2A	Major Faci	Protein Co	41	GC01P039	1.23
RBPJ	Recombina	Protein Co	48	GC04P026	1.23
CUEDC2	CUE Doma	Protein Co	40	GC10M10	1.23
INSL5	Insulin Like	Protein Co	37	GC01M06(	1.23
PRKAA1	Protein Kir	Protein Co	49	GC05M04(	1.22
DAG1	Dystroglyc	Protein Co	48	GC03P049	1.22
P4HA2	Prolyl 4-H	Protein Co	47	GC05M13	1.22
CNTNAP2	Contactin	Protein Co	45	GC07P146	1.22
SYNGR1	Synaptogy	Protein Co	43	GC22P039	1.22
CCL8	C-C Motif	Protein Co	40	GC17P034	1.22
TENM3	Teneurin T	Protein Co	37	GC04P182	1.22
CLEC16A	C-Type Le	Protein Co	37	GC16P010	1.22
CTIF	Cap Bindir	Protein Co	33	GC18P048	1.22
PROX2	Prospero H	Protein Co	31	GC14M07	1.22
KRT38	Keratin 38	Protein Co	36	GC17M04	1.21
AIM2	Absent In	Protein Co	43	GC01M15!	1.21
SMPD1	Sphingom	Protein Co	49	GC11P006	1.21

FAAH	Fatty Acid	Protein Co	48	GC01P046	1.21
CTSE	Cathepsin	Protein Co	44	GC01M20(	1.2
EIF3C	Eukaryotic	Protein Co	39	GC16P028	1.2
NUPR1	Nuclear Pr	Protein Co	37	GC16M02(	1.2
FAM92B	Family Wit	Protein Co	33	GC16M08!	1.2
PFKFB3	6-Phosph(	Protein Co	46	GC10P006	1.2
SLC9A8	Solute Car	Protein Co	40	GC20P049	1.2
ASCL2	Achaete-S	Protein Co	38	GC11M00	1.2
ZFP91	ZFP91 Zin(	Protein Co	34	GC11P058	1.2
CIRBP	Cold Induc	Protein Co	39	GC19P001	1.2
SPHK1	Sphingosir	Protein Co	48	GC17P076	1.19
DBH	Dopamine	Protein Co	51	GC09P133	1.19
DDAH2	Dimethylaı	Protein Co	44	GC06M03	1.19
SYNGAP1	Synaptic R	Protein Co	44	GC06P033	1.19
KIFC1	Kinesin Faı	Protein Co	42	GC06P033	1.19
PPT2	Palmitoyl-	Protein Co	39	GC06P032	1.19
WDR46	WD Repea	Protein Co	37	GC06M03	1.19
VPS52	VPS52 Sub	Protein Co	37	GC06M03	1.19
FKBPL	FKBP Prol	Protein Co	37	GC06M03	1.19
NELFE	Negative E	Protein Co	36	GC06M03	1.19
EGFL8	EGF Like D	Protein Co	35	GC06P033	1.19
ZBTB12	Zinc Finge	Protein Co	34	GC06M03	1.19
SAPCD1	Suppresso	Protein Co	27	GC06P033	1.19
HCG23	HLA Comp	RNA Gene	14	GC06P033	1.19
HCG25	HLA Comp	RNA Gene	13	GC06P033	1.19
NONHSAG045982.2		RNA Gene	5	GC06M03	1.19
HSALNG0049430		RNA Gene	5	GC06M03	1.19
PTPN1	Protein Ty	Protein Co	52	GC20P050	1.19
PIP5K1A	Phosphati(	Protein Co	45	GC01P151	1.19
TNXB	Tenascin X	Protein Co	45	GC06M03	1.19
CRB1	Crumbs C(	Protein Co	44	GC01P197	1.19
RAB5B	RAB5B, M(	Protein Co	41	GC12P055	1.19
DHX16	DEAH-Box	Protein Co	41	GC06M03(	1.19
CDSN	Corneodes	Protein Co	41	GC06M03	1.19
ATP6V1F	ATPase H+	Protein Co	41	GC07P128	1.19
NSMCE2	NSE2 (MM	Protein Co	40	GC08P125	1.19
NCR3	Natural Cy	Protein Co	40	GC06M03	1.19
ABCF1	ATP Bindir	Protein Co	40	GC06P030	1.19
ZFP57	ZFP57 Zin(	Protein Co	40	GC06M02!	1.19
PPP1R10	Protein Ph	Protein Co	39	GC06M03(	1.19
ZNF687	Zinc Finge	Protein Co	39	GC01P151	1.19
GNL1	G Protein	Protein Co	37	GC06M03(	1.19
LRRC2	Leucine Ri	Protein Co	37	GC03M04(	1.19
TCF19	Transcripti	Protein Co	37	GC06P033	1.19
NRM	Nurim	Protein Co	35	GC06M03(	1.19
ATAT1	Alpha Tub	Protein Co	35	GC06P030	1.19
PRR3	Proline Ric	Protein Co	33	GC06P033	1.19
PSORS1C2	Psoriasis S	Protein Co	32	GC06M03	1.19
LY6G5B	Lymphocy	Protein Co	31	GC06P033	1.19
C6orf47	Chromoso	Protein Co	31	GC06M03	1.19
HLA-H	Major Hist	Pseudoger	27	GC06P033	1.19
SPATA48	Spermato(	Protein Co	21	GC07P050	1.19
HCG22	HLA Comp	Protein Co	21	GC06P031	1.19
HLA-J	Major Hist	Pseudoger	18	GC06P033	1.19
HCG18	HLA Comp	RNA Gene	17	GC06M03(	1.19
HCG4B	HLA Comp	RNA Gene	16	GC06M03(	1.19
TPI1P2	Triosepho:	Pseudoger	16	GC07P129	1.19
ZNRD1ASI	Zinc Ribbc	Pseudoger	16	GC06M03(	1.19

HLA-L	Major Hist	Pseudoger	14	GC06P033	1.19
HLA-K	Major Hist	Pseudoger	13	GC06P033	1.19
LINC0127:	Long Inter	RNA Gene	13	GC20P050	1.19
MICD	MHC Class	Pseudoger	12	GC06M03(	1.19
TRIM31-A	TRIM31 Ar	RNA Gene	12	GC06P033	1.19
LINC01271	Long Inter	RNA Gene	12	GC20M05(	1.19
FLNC-AS1	FLNC Antis	RNA Gene	11	GC07M128	1.19
HLA-W	Major Hist	Pseudoger	10	GC06P033	1.19
RPL23AP1	Ribosomal	Pseudoger	10	GC06M03(	1.19
LOC28562	Uncharact	RNA Gene	10	GC05P159	1.19
STK19B	Serine/Thr	Pseudoger	9	GC06P032	1.19
ENSG00000250264		Protein Co	9	GC06M032	1.19
MICE	MHC Class	Pseudoger	9	GC06M03(	1.19
HLA-U	Major Hist	Pseudoger	9	GC06P033	1.19
RPL3P2	Ribosomal	Pseudoger	9	GC06P031	1.19
LINC0200	Long Inter	RNA Gene	9	GC03M04(	1.19
WASHC5-	WASHC5 A	RNA Gene	9	GC08P125	1.19
TSBP1-AS	TSBP1 Anc	RNA Gene	9	GC06P046	1.19
PAIP1P1	Poly(A) Bir	Pseudoger	8	GC06M03(	1.19
ENSG00000249738		Uncategor	8	GC05P159	1.19
ENSG00000230533		RNA Gene	8	GC06P137	1.19
RN7SL636	RNA, 7SL,	Pseudoger	7	GC20M05(	1.19
ENSG00000235620		RNA Gene	7	GC07M05(	1.19
ENSG00000271553		RNA Gene	7	GC07P128	1.19
ENSG00000269667		RNA Gene	7	GC16P086	1.19
ENSG00000270120		RNA Gene	7	GC16P050	1.19
ENSG00000251136		Uncategor	6	GC08M08	1.19
lnc-IKZF1-6		RNA Gene	6	GC07P050	1.19
ENSG00000272540		RNA Gene	6	GC06M03(	1.19
lnc-IRF5-3		RNA Gene	5	GC07P129	1.19
ENSG00000242162		Pseudoger	5	GC07M12	1.19
lnc-NBN-5		RNA Gene	5	GC08M08	1.19
ENSG00000285040		RNA Gene	5	GC16M08	1.19
LOC64526	Pre-MRNA	Pseudoger	5	GC09P114	1.19
RF00017-6602		RNA Gene	5	GC07M128	1.19
RF00005-109		RNA Gene	5	GC20M05(	1.19
ENSG00000237669		Pseudoger	4	GC06M03(	1.19
ENSG00000227766		Pseudoger	4	GC06M03(	1.19
ENSG00000230521		Pseudoger	4	GC06M03(	1.19
NONHSAG019426.2		RNA Gene	4	GC16P050	1.19
ENSG00000224163		Pseudoger	4	GC07M128	1.19
piR-56133-186		RNA Gene	4	GC05M15	1.19
LOC10537	Uncharact	RNA Gene	3	GC20P050	1.19
SELENBP1	Selenium E	Protein Co	45	GC01M151	1.18
MAZ	MYC Asso	Protein Co	41	GC16P029	1.18
CHMP5	Charged M	Protein Co	40	GC09P033	1.18
GC	GC Vitami	Protein Co	44	GC04M071	1.18
AR	Androgen	Protein Co	54	GC0XP067	1.17
HMGCR	3-Hydroxy	Protein Co	47	GC05P075	1.17
CNR2	Cannabinc	Protein Co	47	GC01M02	1.16
HHIP	Hedgehog	Protein Co	43	GC04P144	1.16
HRH1	Histamine	Protein Co	47	GC03P011	1.15
BIN1	Bridging Ir	Protein Co	48	GC02M127	1.15
CCDC88B	Coiled-Co	Protein Co	33	GC11P064	1.14
NR3C2	Nuclear Re	Protein Co	49	GC04M148	1.14
SIK2	Salt Induci	Protein Co	45	GC11P111	1.14
LILRB4	Leukocyte	Protein Co	41	GC19P054	1.14
CCL13	C-C Motif	Protein Co	40	GC17P034	1.14

MIR133A1	MicroRNA	RNA Gene	18	GC18M021	1.14
PAH	Phenylalar	Protein Co	50	GC12M102	1.13
MRTFA	Myocardin	Protein Co	33	GC22M044	1.13
DSC1	Desmocoll	Protein Co	39	GC18M031	1.13
IL34	Interleukin	Protein Co	40	GC16P070	1.13
ETV6	ETS Varian	Protein Co	48	GC12P011	1.12
CXCL16	C-X-C Mo	Protein Co	41	GC17M004	1.12
IKZF4	IKAROS Fa	Protein Co	37	GC12P056	1.11
MIR346	MicroRNA	RNA Gene	19	GC10M086	1.11
PDZK1	PDZ Doma	Protein Co	41	GC01M145	1.11
MIR449A	MicroRNA	RNA Gene	21	GC05M055	1.11
SPINK1	Serine Pep	Protein Co	45	GC05M147	1.11
PLAA	Phospholip	Protein Co	44	GC09M026	1.11
MAPK10	Mitogen-A	Protein Co	52	GC04M085	1.09
FAP	Fibroblast	Protein Co	44	GC02M162	1.09
SLC11A2	Solute Car	Protein Co	48	GC12M050	1.08
DSC3	Desmocoll	Protein Co	43	GC18M030	1.08
LY96	Lymphocy	Protein Co	43	GC08P073	1.08
MIR590	MicroRNA	RNA Gene	19	GC07P074	1.08
RAC2	Rac Family	Protein Co	52	GC22M037	1.08
BMP2	Bone Morp	Protein Co	48	GC20P006	1.08
KLF1	Kruppel Lil	Protein Co	46	GC19M012	1.08
CST3	Cystatin C	Protein Co	45	GC20M023	1.08
CDB2	Corneal Dy	Genetic Lo	5	GC10U990	1.08
PA2G4	Proliferatic	Protein Co	42	GC12P056	1.08
PEBP1	Phosphatid	Protein Co	46	GC12P118	1.08
PRKG1	Protein Kir	Protein Co	52	GC10P050	1.07
ACAT1	Acetyl-Co	Protein Co	51	GC11P108	1.07
CD247	CD247 Mo	Protein Co	50	GC01M167	1.07
PIK3CG	Phosphatid	Protein Co	49	GC07P106	1.07
ABCC6	ATP Bindir	Protein Co	46	GC16M016	1.07
B3GAT1	Beta-1,3-G	Protein Co	44	GC11M134	1.07
WARS1	Tryptopha	Protein Co	39	GC14M100	1.07
BCL2L15	BCL2 Like	Protein Co	34	GC01M113	1.07
NPY	Neuropep	Protein Co	47	GC07P024	1.07
UBE2N	Ubiquitin C	Protein Co	48	GC12M093	1.06
NELL1	Neural EGI	Protein Co	40	GC11P020	1.06
TIMP4	TIMP Meta	Protein Co	42	GC03M012	1.05
CFL1	Cofilin 1	Protein Co	47	GC11M065	1.04
CUL2	Cullin 2	Protein Co	43	GC10M035	1.04
USP20	Ubiquitin S	Protein Co	41	GC09P129	1.04
UBAP2L	Ubiquitin A	Protein Co	35	GC01P154	1.04
TMEM258	Transmem	Protein Co	31	GC11M061	1.04
EGR2	Early Grow	Protein Co	44	GC10M062	1.04
BRMS1	BRMS1 Tra	Protein Co	39	GC11M066	1.04
UBASH3A	Ubiquitin A	Protein Co	39	GC21P042	1.04
TRIM39-R	TRIM39-R	Protein Co	22	GC06P033	1.04
ADM	Adrenome	Protein Co	45	GC11P010	1.02
USP7	Ubiquitin S	Protein Co	48	GC16M008	1.02
IRAK3	Interleukin	Protein Co	50	GC12P066	1.02
ENPP2	Ectonucleo	Protein Co	45	GC08M119	1.02
NUP107	Nucleopor	Protein Co	43	GC12P068	1
LCT	Lactase	Protein Co	44	GC02M135	0.99
ITGA1	Integrin Su	Protein Co	43	GC05P052	0.99
ARHGEF6	Rac/Cdc42	Protein Co	43	GC0XM136	0.99
REEP6	Receptor A	Protein Co	40	GC19P001	0.99
CAMK2A	Calcium/C	Protein Co	52	GC05M150	0.98
RAD50	RAD50 Do	Protein Co	51	GC05P132	0.98

TUBB	Tubulin Be	Protein Co	51	GC06P030	0.98
THRA	Thyroid Ho	Protein Co	50	GC17P040	0.98
FES	FES Proto-	Protein Co	50	GC15P090	0.98
PRKAB1	Protein Kir	Protein Co	49	GC12P119	0.98
PTGIS	Prostaglan	Protein Co	48	GC20M04	0.98
PRKAR2A	Protein Kir	Protein Co	48	GC03M04	0.98
SCNN1A	Sodium Ch	Protein Co	48	GC12M00	0.98
GLS	Glutamina	Protein Co	48	GC02P190	0.98
GALT	Galactose-	Protein Co	48	GC09P034	0.98
F12	Coagulatic	Protein Co	48	GC05M17	0.98
CACNA1S	Calcium V	Protein Co	48	GC01M20	0.98
PDXK	Pyridoxal	Protein Co	48	GC21P043	0.98
PRKD2	Protein Kir	Protein Co	47	GC19M04	0.98
PPP1CB	Protein Ph	Protein Co	47	GC02P028	0.98
CIT	Citron Rhc	Protein Co	47	GC12M11	0.98
GRK6	G Protein-	Protein Co	47	GC05P177	0.98
HINT1	Histidine T	Protein Co	47	GC05M13	0.98
NEK9	NIMA Rela	Protein Co	47	GC14M07	0.98
CTSF	Cathepsin	Protein Co	47	GC11M06	0.98
ATXN2	Ataxin 2	Protein Co	47	GC12M11	0.98
MAN1B1	Mannosida	Protein Co	47	GC09P137	0.98
AMT	Aminomet	Protein Co	47	GC03M04	0.98
ERN1	Endoplasm	Protein Co	47	GC17M06	0.98
SLC25A20	Solute Car	Protein Co	47	GC03M04	0.98
IMPDH2	Inosine Mo	Protein Co	47	GC03M04	0.98
POFUT1	Protein O-	Protein Co	46	GC20P032	0.98
POLR1D	RNA Polyr	Protein Co	46	GC13P027	0.98
RAPGEF3	Rap Guani	Protein Co	46	GC12M04	0.98
IL11RA	Interleukin	Protein Co	46	GC09P034	0.98
VAMP1	Vesicle As	Protein Co	46	GC12M00	0.98
EFNB2	Ephrin B2	Protein Co	45	GC13M10	0.98
PLXNB1	Plexin B1	Protein Co	45	GC03M04	0.98
SLC34A1	Solute Car	Protein Co	45	GC05P177	0.98
TMED10	Transmem	Protein Co	45	GC14M07	0.98
SEC24C	SEC24 Hor	Protein Co	45	GC10P073	0.98
CHRNB2	Cholinergi	Protein Co	45	GC01P154	0.98
ANXA11	Annexin A	Protein Co	45	GC10M08	0.98
TNNT3	Troponin T	Protein Co	45	GC11P001	0.98
TUFM	Tu Transla	Protein Co	45	GC16M02	0.98
UBE2G2	Ubiquitin (	Protein Co	45	GC21M04	0.98
LAMB2	Laminin Su	Protein Co	45	GC03M04	0.98
EFEMP2	EGF Conta	Protein Co	44	GC11M06	0.98
PNPLA8	Patatin Lik	Protein Co	44	GC07M10	0.98
POLR2E	RNA Polyr	Protein Co	44	GC19M00	0.98
PPIF	Peptidylpr	Protein Co	44	GC10P083	0.98
PPOX	Protoporp	Protein Co	44	GC01P161	0.98
SLC26A4	Solute Car	Protein Co	44	GC07P107	0.98
PTPRK	Protein Ty	Protein Co	44	GC06M12	0.98
PPP1R1B	Protein Ph	Protein Co	44	GC17P039	0.98
STK36	Serine/Thr	Protein Co	44	GC02P218	0.98
HHEX	Hematopo	Protein Co	44	GC10P092	0.98
HDAC11	Histone De	Protein Co	44	GC03P013	0.98
HOXA10	Homeobo:	Protein Co	44	GC07M02	0.98
SENP1	SUMO Spe	Protein Co	44	GC12M04	0.98
HIBCH	3-Hydroxy	Protein Co	44	GC02M19	0.98
ABI1	Abl Interac	Protein Co	44	GC10M02	0.98
ADAMTS4	ADAM Me	Protein Co	44	GC01M16	0.98
BATF	Basic Leuc	Protein Co	44	GC14P075	0.98

AUH	AU RNA B	Protein Co	44	GC09M091	0.98
FCER1G	Fc Fragme	Protein Co	44	GC01P161	0.98
LTBP2	Latent Tra	Protein Co	44	GC14M074	0.98
LZTR1	Leucine Zi	Protein Co	44	GC22P020	0.98
ARPC1B	Actin Relat	Protein Co	44	GC07P099	0.98
NPEPPS	Aminopep	Protein Co	43	GC17P047	0.98
EMP2	Epithelial M	Protein Co	43	GC16M010	0.98
NRCAM	Neuronal C	Protein Co	43	GC07M108	0.98
RAB1B	RAB1B, Me	Protein Co	43	GC11P066	0.98
TCAP	Titin-Cap	Protein Co	43	GC17P039	0.98
TCP1	T-Comple	Protein Co	43	GC06M159	0.98
PRPF6	Pre-MRNA	Protein Co	43	GC20P063	0.98
SCNN1D	Sodium Ch	Protein Co	43	GC01P001	0.98
MUS81	MUS81 Str	Protein Co	43	GC11P065	0.98
HOXA11	Homeobo:	Protein Co	43	GC07M027	0.98
NEUROD2	Neuronal I	Protein Co	43	GC17M039	0.98
RPL3	Ribosomal	Protein Co	43	GC22M044	0.98
CCNT1	Cyclin T1	Protein Co	43	GC12M048	0.98
AFF4	AF4/FMR2	Protein Co	43	GC05M132	0.98
ASH1L	ASH1 Like	Protein Co	43	GC01M155	0.98
LHX3	LIM Home	Protein Co	43	GC09M136	0.98
UQCRC1	Ubiquinol-	Protein Co	43	GC03M048	0.98
TRPM2	Transient I	Protein Co	43	GC21P044	0.98
TOM1	Target Of	Protein Co	43	GC22P035	0.98
HLA-F	Major Hist	Protein Co	42	GC06P033	0.98
DSE	Dermatan	Protein Co	42	GC06P116	0.98
THBS3	Thrombos	Protein Co	42	GC01M155	0.98
PPIL2	Peptidylpr	Protein Co	42	GC22P024	0.98
JDP2	Jun Dimeri	Protein Co	42	GC14P075	0.98
KLC2	Kinesin Lig	Protein Co	42	GC11P066	0.98
CLK2	CDC Like K	Protein Co	42	GC01M155	0.98
GNPDA1	Glucosami	Protein Co	42	GC05M141	0.98
HIBADH	3-Hydroxy	Protein Co	42	GC07M027	0.98
AGPAT1	1-Acylglyc	Protein Co	42	GC06M032	0.98
LTBP3	Latent Tra	Protein Co	42	GC11M065	0.98
BRAP	BRCA1 Ass	Protein Co	42	GC12M111	0.98
FLVCR2	FLVCR Her	Protein Co	42	GC14P075	0.98
ANAPC2	Anaphase	Protein Co	42	GC09M137	0.98
TOP3B	DNA Topo	Protein Co	42	GC22M021	0.98
KIF3A	Kinesin Fa	Protein Co	42	GC05M132	0.98
VAMP3	Vesicle Ass	Protein Co	42	GC01P007	0.98
UBE2J2	Ubiquitin C	Protein Co	42	GC01M001	0.98
PHB2	Prohibitin	Protein Co	42	GC12M006	0.98
FLAD1	Flavin Ade	Protein Co	42	GC01P154	0.98
RBMX	RNA Bindi	Protein Co	42	GC0XM136	0.98
CST6	Cystatin E/	Protein Co	41	GC11P066	0.98
NDUFAF3	NADH:Ubi	Protein Co	41	GC03P049	0.98
RBM4	RNA Bindi	Protein Co	41	GC11P066	0.98
POLA2	DNA Polyr	Protein Co	41	GC11P065	0.98
SETD1A	SET Doma	Protein Co	41	GC16P030	0.98
PPA2	Inorganic I	Protein Co	41	GC04M105	0.98
ITIH3	Inter-Alph	Protein Co	41	GC03P052	0.98
PASK	PAS Doma	Protein Co	41	GC02M241	0.98
DBN1	Drebrin 1	Protein Co	41	GC05M177	0.98
HIPK1	Homeodo	Protein Co	41	GC01P113	0.98
NCAPD2	Non-SMC	Protein Co	41	GC12P006	0.98
SART1	Spliceosor	Protein Co	41	GC11P065	0.98
DPM3	Dolichyl-P	Protein Co	41	GC01M155	0.98

EMG1	EMG1 N1-	Protein Co	41	GC12P006	0.98
OLIG2	Oligodenc	Protein Co	41	GC21P033	0.98
GDI2	GDP Disso	Protein Co	41	GC10M005	0.98
CELSR3	Cadherin E	Protein Co	41	GC03M048	0.98
ATP6V1G3	ATPase H+	Protein Co	41	GC01M198	0.98
B3GNT2	UDP-GlcN	Protein Co	41	GC02P062	0.98
B4GALT5	Beta-1,4-(	Protein Co	41	GC20M049	0.98
LPAR5	Lysophosp	Protein Co	41	GC12M006	0.98
FDX1	Ferredoxin	Protein Co	41	GC11P110	0.98
CAMTA1	Calmoduli	Protein Co	41	GC01P006	0.98
APOM	Apolipopr	Protein Co	41	GC06P033	0.98
MED1	Mediator (	Protein Co	41	GC17M039	0.98
TNS1	Tensin 1	Protein Co	41	GC02M217	0.98
LIMD1	LIM Doma	Protein Co	41	GC03P045	0.98
TTLL5	Tubulin Ty	Protein Co	41	GC14P075	0.98
FKRP	Fukutin Re	Protein Co	41	GC19P046	0.98
NRBP1	Nuclear Re	Protein Co	40	GC02P027	0.98
RALGAPA1	Ral GTPase	Protein Co	40	GC14M035	0.98
KDELR2	KDEL Endo	Protein Co	40	GC07M006	0.98
IFT172	Intraflagell	Protein Co	40	GC02M027	0.98
PPP2R3C	Protein Ph	Protein Co	40	GC14M035	0.98
SLC6A7	Solute Car	Protein Co	40	GC05P150	0.98
TAS1R3	Taste 1 Re	Protein Co	40	GC01P001	0.98
SLC2A13	Solute Car	Protein Co	40	GC12M039	0.98
SLC16A10	Solute Car	Protein Co	40	GC06P111	0.98
STARD3	StAR Relat	Protein Co	40	GC17P039	0.98
SON	SON DNA	Protein Co	40	GC21P033	0.98
GRHL3	Grainyhea	Protein Co	40	GC01P024	0.98
RIN1	Ras And R	Protein Co	40	GC11M066	0.98
HIF3A	Hypoxia In	Protein Co	40	GC19P046	0.98
NDST2	N-Deacety	Protein Co	40	GC10M073	0.98
NAB1	NGFI-A Bi	Protein Co	40	GC02P190	0.98
COG5	Componen	Protein Co	40	GC07M107	0.98
CPSF3	Cleavage A	Protein Co	40	GC02P009	0.98
GLYAT	Glycine-N	Protein Co	40	GC11M059	0.98
RPL37	Ribosomal	Protein Co	40	GC05M040	0.98
RPL24	Ribosomal	Protein Co	40	GC03M101	0.98
OLIG1	Oligodenc	Protein Co	40	GC21P033	0.98
DAP3	Death Ass	Protein Co	40	GC01P155	0.98
CD248	CD248 Mo	Protein Co	40	GC11M066	0.98
ACTR1A	Actin Relat	Protein Co	40	GC10M102	0.98
BAZ1A	Bromodom	Protein Co	40	GC14M034	0.98
B3GALT6	Beta-1,3-(	Protein Co	40	GC01P001	0.98
CARD6	Caspase R	Protein Co	40	GC05P040	0.98
AAGAB	Alpha And	Protein Co	40	GC15M067	0.98
APOBEC3A	Apolipopr	Protein Co	40	GC22P038	0.98
ASAP2	ArfGAP Wi	Protein Co	40	GC02P009	0.98
ASIC2	Acid Sensi	Protein Co	40	GC17M033	0.98
ACYP1	Acylphosp	Protein Co	40	GC14M075	0.98
ATP8B2	ATPase Ph	Protein Co	40	GC01P154	0.98
FAU	FAU Ubiqu	Protein Co	40	GC11M065	0.98
THEM4	Thioestera	Protein Co	40	GC01M151	0.98
THEMIS	Thymocyte	Protein Co	40	GC06M127	0.98
UQCR10	Ubiquinol-	Protein Co	40	GC22P029	0.98
USP19	Ubiquitin S	Protein Co	40	GC03M049	0.98
USP21	Ubiquitin S	Protein Co	40	GC01P161	0.98
WTAP	WT1 Asso	Protein Co	40	GC06P159	0.98
STK19	Serine/Thr	Protein Co	39	GC06P031	0.98

RCE1	Ras Conve	Protein Co	39	GC11P066	0.98
PKIG	CAMP-De	Protein Co	39	GC20P044	0.98
SNX27	Sorting Ne	Protein Co	39	GC01P151	0.98
ITGB1BP1	Integrin Su	Protein Co	39	GC02M009	0.98
RAPGEF6	Rap Guani	Protein Co	39	GC05M131	0.98
SH3PXD2E	SH3 And F	Protein Co	39	GC05M172	0.98
PAPOLG	Poly(A) Po	Protein Co	39	GC02P060	0.98
PYGO2	Pygopus F	Protein Co	39	GC01M154	0.98
RBM17	RNA Bindi	Protein Co	39	GC10P006	0.98
KLHDC8B	Kelch Dom	Protein Co	39	GC03P049	0.98
RFT1	RFT1 Hom	Protein Co	39	GC03M053	0.98
DAZAP1	DAZ Assoc	Protein Co	39	GC19P001	0.98
RLN2	Relaxin 2	Protein Co	39	GC09M005	0.98
DAGLB	Diacylglyc	Protein Co	39	GC07M006	0.98
DRAP1	DR1 Assoc	Protein Co	39	GC11P065	0.98
HBP1	HMG-Box	Protein Co	39	GC07P107	0.98
HCN3	Hyperpola	Protein Co	39	GC01P155	0.98
MPPE1	Metalloph	Protein Co	39	GC18M016	0.98
MRPL11	Mitochonc	Protein Co	39	GC11M066	0.98
DIS3L	DIS3 Like E	Protein Co	39	GC15P066	0.98
SF3B2	Splicing Fa	Protein Co	39	GC11P066	0.98
GPSM1	G Protein S	Protein Co	39	GC09P136	0.98
CH25H	Cholesterc	Protein Co	39	GC10M089	0.98
GPN1	GPN-Loop	Protein Co	39	GC02P027	0.98
RPL26L1	Ribosomal	Protein Co	39	GC05P172	0.98
RSPH4A	Radial Spo	Protein Co	39	GC06P116	0.98
CDK11A	Cyclin Dep	Protein Co	39	GC01M001	0.98
CDK11B	Cyclin Dep	Protein Co	39	GC01M001	0.98
BBS1	Bardet-Bie	Protein Co	39	GC11P066	0.98
AURKAIP1	Aurora Kin	Protein Co	39	GC01M001	0.98
LRCH4	Leucine Ri	Protein Co	39	GC07M100	0.98
MAN2A2	Mannosida	Protein Co	39	GC15P090	0.98
ARIH2	Ariadne RE	Protein Co	39	GC03P048	0.98
APOL6	Apolipopr	Protein Co	39	GC22P035	0.98
TOR1B	Torsin Fam	Protein Co	39	GC09P129	0.98
TIPIN	TIMELESS	Protein Co	39	GC15M066	0.98
YY1AP1	YY1 Assoc	Protein Co	39	GC01M155	0.98
USP37	Ubiquitin S	Protein Co	39	GC02M218	0.98
UCKL1	Uridine-Cy	Protein Co	39	GC20M063	0.98
USP36	Ubiquitin S	Protein Co	39	GC17M078	0.98
ZBTB40	Zinc Finge	Protein Co	39	GC01P022	0.98
UBE2Q1	Ubiquitin C	Protein Co	39	GC01M154	0.98
TRIM8	Tripartite M	Protein Co	39	GC10P102	0.98
PGAP3	Post-GPI A	Protein Co	39	GC17M039	0.98
PGS1	Phosphatic	Protein Co	39	GC17P078	0.98
SLC25A28	Solute Car	Protein Co	39	GC10M099	0.98
NRBF2	Nuclear Re	Protein Co	38	GC10P063	0.98
SEC31B	SEC31 Hor	Protein Co	38	GC10M100	0.98
PMF1	Polyamine	Protein Co	38	GC01P156	0.98
SLC12A9	Solute Car	Protein Co	38	GC07P100	0.98
REXO2	RNA Exonu	Protein Co	38	GC11P114	0.98
NDOR1	NADPH De	Protein Co	38	GC09P137	0.98
DIDO1	Death Indu	Protein Co	38	GC20M062	0.98
SDF4	Stromal Ce	Protein Co	38	GC01M001	0.98
SENP7	SUMO Spe	Protein Co	38	GC03M101	0.98
DNAJC27	DnaJ Heat	Protein Co	38	GC02M024	0.98
MED24	Mediator C	Protein Co	38	GC17M040	0.98
CBLL1	Cbl Proto-	Protein Co	38	GC07P107	0.98

ADO	2-Aminoe	Protein Co	38	GC10P062	0.98
ATP6V0E1	ATPase H	Protein Co	38	GC05P172	0.98
LYRM7	LYR Motif	Protein Co	38	GC05P131	0.98
ERRFI1	ERBB Rece	Protein Co	38	GC01M008	0.98
ADGRL2	Adhesion	Protein Co	38	GC01P081	0.98
TRAPPC10	Trafficking	Protein Co	38	GC21P044	0.98
WIPF2	WAS/WAS	Protein Co	38	GC17P040	0.98
ING4	Inhibitor C	Protein Co	38	GC12M006	0.98
STAC2	SH3 And C	Protein Co	38	GC17M039	0.98
DUSP8	Dual Speci	Protein Co	37	GC11M001	0.98
RBM7	RNA Bindi	Protein Co	37	GC11P114	0.98
PRELID1	PRELI Don	Protein Co	37	GC05P177	0.98
SPSB2	SplA/Ryan	Protein Co	37	GC12M006	0.98
SDF2L1	Stromal Ce	Protein Co	37	GC22P023	0.98
SNAPC5	Small Nucl	Protein Co	37	GC15M066	0.98
PLB1	Phospholi	Protein Co	37	GC02P028	0.98
PPP1R11	Protein Ph	Protein Co	37	GC06P033	0.98
RAB24	RAB24, Me	Protein Co	37	GC05M177	0.98
P4HTM	Prolyl 4-H	Protein Co	37	GC03P049	0.98
QSOX2	Quiescin S	Protein Co	37	GC09M136	0.98
INO80	INO80 Cor	Protein Co	37	GC15M040	0.98
ST7	Suppressio	Protein Co	37	GC07P117	0.98
FNIP1	Folliculin I	Protein Co	37	GC05M131	0.98
NFKBIZ	NFKB Inhib	Protein Co	37	GC03P101	0.98
RNPEPL1	Arginyl An	Protein Co	37	GC02P240	0.98
COMMD7	COMM Do	Protein Co	37	GC20M032	0.98
MRPL20	Mitochond	Protein Co	37	GC01M001	0.98
DNAH17	Dynein Ax	Protein Co	37	GC17M078	0.98
RTF1	RTF1 Hom	Protein Co	37	GC15P041	0.98
GPR25	G Protein-	Protein Co	37	GC01P200	0.98
CEP76	Centrosom	Protein Co	37	GC18M016	0.98
CEP192	Centrosom	Protein Co	37	GC18P013	0.98
DUSP12	Dual Speci	Protein Co	37	GC01P161	0.98
RNF123	Ring Finge	Protein Co	37	GC03P049	0.98
MYOZ1	Myozenin	Protein Co	37	GC10M073	0.98
DPH5	Diphthami	Protein Co	37	GC01M100	0.98
RSPH3	Radial Spo	Protein Co	37	GC06M158	0.98
BCAP29	B Cell Rece	Protein Co	37	GC07P107	0.98
BAG6	BAG Coch	Protein Co	37	GC06M031	0.98
LPCAT3	Lysophosp	Protein Co	37	GC12M006	0.98
FBXL20	F-Box And	Protein Co	37	GC17M039	0.98
BNIP1	BCL2 Inter	Protein Co	37	GC05P173	0.98
LY6G6F	Lymphocy	Protein Co	37	GC06P031	0.98
FNDC3A	Fibronecti	Protein Co	37	GC13P048	0.98
ASB8	Ankyrin Re	Protein Co	37	GC12M048	0.98
ASCC2	Activating	Protein Co	37	GC22M029	0.98
ATAD3B	ATPase Fa	Protein Co	37	GC01P001	0.98
TNRC6C	Trinucleoti	Protein Co	37	GC17P077	0.98
TRIM26	Tripartite M	Protein Co	37	GC06M030	0.98
UBLCP1	Ubiquitin L	Protein Co	37	GC05P159	0.98
YTHDF1	YTH N6-M	Protein Co	37	GC20M063	0.98
UBAC1	UBA Doma	Protein Co	37	GC09M135	0.98
TSPAN32	Tetraspani	Protein Co	37	GC11P002	0.98
SLC9A4	Solute Car	Protein Co	37	GC02P102	0.98
ZNF384	Zinc Finge	Protein Co	37	GC12M006	0.98
TAPBPL	TAP Bindir	Protein Co	36	GC12P006	0.98
RBM4B	RNA Bindi	Protein Co	36	GC11M066	0.98
PNRC2	Proline Ric	Protein Co	36	GC01P023	0.98

PLEKHG6	Pleckstrin	Protein Co	36	GC12P006	0.98
TATDN1	TatD DNas	Protein Co	36	GC08M124	0.98
TBC1D8	TBC1 Dom	Protein Co	36	GC02M101	0.98
TBKBP1	TBK1 Bindi	Protein Co	36	GC17P047	0.98
SHISA5	Shisa Fami	Protein Co	36	GC03M048	0.98
PWP2	PWP2 Sma	Protein Co	36	GC21P044	0.98
QRICH1	Glutamine	Protein Co	36	GC03M049	0.98
TMEM199	Transmem	Protein Co	36	GC17P028	0.98
LSM14A	LSM14A M	Protein Co	36	GC19P034	0.98
LY6G6C	Lymphocy	Protein Co	36	GC06M032	0.98
EAPP	E2F Associ	Protein Co	36	GC14M034	0.98
NAALADL1	N-Acetyla	Protein Co	36	GC11M065	0.98
SGIP1	SH3GL Inte	Protein Co	36	GC01P066	0.98
SBK1	SH3 Doma	Protein Co	36	GC16P028	0.98
SERBP1	SERPINE1	Protein Co	36	GC01M067	0.98
CEP89	Centrosom	Protein Co	36	GC19M032	0.98
CENPO	Centromer	Protein Co	36	GC02P024	0.98
MXRA8	Matrix Rer	Protein Co	36	GC01M001	0.98
NKD1	NKD Inhib	Protein Co	36	GC16P050	0.98
NT5DC2	5'-Nucleot	Protein Co	36	GC03M052	0.98
OIP5	Opa Intera	Protein Co	36	GC15M041	0.98
CCNL2	Cyclin L2	Protein Co	36	GC01M001	0.98
FAM177A1	Family Wit	Protein Co	36	GC14P035	0.98
FCAMR	Fc Fragme	Protein Co	36	GC01M206	0.98
FITM2	Fat Storag	Protein Co	36	GC20M044	0.98
CASC3	CASC3 Exc	Protein Co	36	GC17P040	0.98
ACAP3	ArfGAP Wi	Protein Co	36	GC01M001	0.98
ASB6	Ankyrin Re	Protein Co	36	GC09M129	0.98
ANKZF1	Ankyrin Re	Protein Co	36	GC02P219	0.98
BOD1	Biorientati	Protein Co	36	GC05M173	0.98
TRIM10	Tripartite M	Protein Co	36	GC06M030	0.98
VARS1	Valyl-TRN.	Protein Co	36	GC06M032	0.98
ZC3H12C	Zinc Finge	Protein Co	36	GC11P110	0.98
ZNF142	Zinc Finge	Protein Co	36	GC02M218	0.98
UBTD2	Ubiquitin D	Protein Co	36	GC05M172	0.98
YIF1A	Yip1 Intera	Protein Co	36	GC11M066	0.98
VPS51	VPS51 Sub	Protein Co	36	GC11P065	0.98
RNF145	Ring Finge	Protein Co	35	GC05M159	0.98
NPAS4	Neuronal P	Protein Co	35	GC11P066	0.98
ENDOU	Endonucle	Protein Co	35	GC12M047	0.98
THAP7	THAP Don	Protein Co	35	GC22M020	0.98
PRM2	Protamine	Protein Co	35	GC16M011	0.98
IFNLR1	Interferon	Protein Co	35	GC01M024	0.98
IFFO1	Intermedia	Protein Co	35	GC12M006	0.98
SNN	Stannin	Protein Co	35	GC16P011	0.98
TCTEX1D1	Tctex1 Do	Protein Co	35	GC01P066	0.98
KANSL2	KAT8 Regu	Protein Co	35	GC12M048	0.98
RAVER1	Ribonucle	Protein Co	35	GC19M010	0.98
TCHHL1	Trichohyal	Protein Co	35	GC01M152	0.98
QPCTL	Glutaminy	Protein Co	35	GC19P045	0.98
PSMG2	Proteasom	Protein Co	35	GC18P013	0.98
GRAMD1B	GRAM Dor	Protein Co	35	GC11P123	0.98
CMC1	C-X9-C M	Protein Co	35	GC03P028	0.98
CYTL1	Cytokine L	Protein Co	35	GC04M005	0.98
DALRD3	DALR Anti	Protein Co	35	GC03M049	0.98
NGRN	Neugrin, N	Protein Co	35	GC15P090	0.98
DYDC1	DPY30 Do	Protein Co	35	GC10M080	0.98
DYDC2	DPY30 Do	Protein Co	35	GC10P080	0.98

MIEN1	Migration	Protein Co	35	GC17M039	0.98
MIER1	MIER1 Tra	Protein Co	35	GC01P066	0.98
GON4L	Gon-4 Like	Protein Co	35	GC01M155	0.98
DONSON	Downstrea	Protein Co	35	GC21M033	0.98
DNAJC28	DnaJ Heat	Protein Co	35	GC21M033	0.98
MXD3	MAX Dime	Protein Co	35	GC05M177	0.98
DUPD1	Dual Speci	Protein Co	35	GC10M075	0.98
MYRF	Myelin Reg	Protein Co	35	GC11P061	0.98
OAZ3	Ornithine	Protein Co	35	GC01P151	0.98
OLFML2B	Olfactome	Protein Co	35	GC01M161	0.98
GID8	GID Comp	Protein Co	35	GC20P062	0.98
GAL3ST2	Galactose-	Protein Co	35	GC02P241	0.98
CDC42SE2	CDC42 Sm	Protein Co	35	GC05P131	0.98
MFSD9	Major Faci	Protein Co	35	GC02M102	0.98
FUT11	Fucosyltra	Protein Co	35	GC10P073	0.98
FYB1	FYN Bindir	Protein Co	35	GC05M039	0.98
ABHD16A	Abhydrola	Protein Co	35	GC06M031	0.98
FAM53B	Family Wit	Protein Co	35	GC10M124	0.98
EVX1	Even-Skip	Protein Co	35	GC07P027	0.98
EXD1	Exonuclea	Protein Co	35	GC15M041	0.98
AMZ1	Archaelysi	Protein Co	35	GC07P002	0.98
ANKRD30A	Ankyrin Re	Protein Co	35	GC10P037	0.98
TRIM15	Tripartite M	Protein Co	35	GC06P033	0.98
ZNF366	Zinc Finge	Protein Co	35	GC05M072	0.98
WDR43	WD Repea	Protein Co	35	GC02P028	0.98
ZC3H4	Zinc Finge	Protein Co	35	GC19M047	0.98
ZWILCH	Zwilch Kin	Protein Co	35	GC15P066	0.98
PTRHD1	Peptidyl-T	Protein Co	35	GC02M024	0.98
SPATA2	Spermatog	Protein Co	35	GC20M049	0.98
PPP1R18	Protein Ph	Protein Co	35	GC06M030	0.98
PAXBP1	PAX3 And	Protein Co	34	GC21M032	0.98
SKOR1	SKI Family	Protein Co	34	GC15P067	0.98
TMCO4	Transmem	Protein Co	34	GC01M019	0.98
TMEM106C	Transmem	Protein Co	34	GC12P047	0.98
C9orf78	Chromoso	Protein Co	34	GC09M129	0.98
NICN1	Nicolin 1	Protein Co	34	GC03M049	0.98
EIF1AD	Eukaryotic	Protein Co	34	GC11M065	0.98
NACC2	NACC Fam	Protein Co	34	GC09M136	0.98
MUSTN1	Musculosk	Protein Co	34	GC03M052	0.98
ELOVL3	ELOVL Fat	Protein Co	34	GC10P102	0.98
RPAP3	RNA Polyr	Protein Co	34	GC12M047	0.98
RPP25L	Ribonucle	Protein Co	34	GC09M034	0.98
FRMD8	FERM Don	Protein Co	34	GC11P065	0.98
ACRBP	Acrosin Bi	Protein Co	34	GC12M006	0.98
FBXO24	F-Box Prot	Protein Co	34	GC07P100	0.98
C11orf68	Chromoso	Protein Co	34	GC11M065	0.98
C6orf15	Chromoso	Protein Co	34	GC06M031	0.98
FAM118A	Family Wit	Protein Co	34	GC22P045	0.98
KRTCAP2	Keratinocy	Protein Co	34	GC01M155	0.98
UQCC1	Ubiquinol-	Protein Co	34	GC20M035	0.98
ZFPL1	Zinc Finge	Protein Co	34	GC11P065	0.98
YLPM1	YLP Motif	Protein Co	34	GC14P074	0.98
LIX1	Limb And	Protein Co	34	GC05M097	0.98
ZNF507	Zinc Finge	Protein Co	34	GC19P032	0.98
SUPT7L	SPT7 Like,	Protein Co	33	GC02M027	0.98
IQCH	IQ Motif C	Protein Co	33	GC15P067	0.98
TCTA	T Cell Leuk	Protein Co	33	GC03P049	0.98
PCMTD2	Protein-L-	Protein Co	33	GC20P064	0.98

C5orf24	Chromoso	Protein Co	33	GC05P134	0.98
LY6G6D	Lymphocy	Protein Co	33	GC06P031	0.98
CNEP1R1	CTD Nucle	Protein Co	33	GC16P050	0.98
RIC8B	RIC8 Guan	Protein Co	33	GC12P106	0.98
EHBP1L1	EH Domaii	Protein Co	33	GC11P065	0.98
DXO	Decapping	Protein Co	33	GC06M031	0.98
MIER2	MIER Fami	Protein Co	33	GC19M000	0.98
GPATCH1	G-Patch D	Protein Co	33	GC19P033	0.98
GPATCH2L	G-Patch D	Protein Co	33	GC14P076	0.98
GMEB2	Glucocorti	Protein Co	33	GC20M063	0.98
NUDT13	Nudix Hyd	Protein Co	33	GC10P073	0.98
RSBN1	Round Spe	Protein Co	33	GC01M113	0.98
OR10AD1	Olfactory	Protein Co	33	GC12M048	0.98
CCDC51	Coiled-Co	Protein Co	33	GC03M048	0.98
FCF1	FCF1 RRN	Protein Co	33	GC14P074	0.98
LURAP1L	Leucine Ri	Protein Co	33	GC09P012	0.98
BPIFA2	BPI Fold C	Protein Co	33	GC20P033	0.98
BPIFB3	BPI Fold C	Protein Co	33	GC20P033	0.98
BPIFB4	BPI Fold C	Protein Co	33	GC20P033	0.98
CARS1	Cysteinyl-	Protein Co	33	GC11M003	0.98
KRT222	Keratin 22	Protein Co	33	GC17M040	0.98
TNP2	Transition	Protein Co	33	GC16M011	0.98
TRIM4	Tripartite	Protein Co	33	GC07M099	0.98
TRIM40	Tripartite	Protein Co	33	GC06P033	0.98
TTPAL	Alpha Toc	Protein Co	33	GC20P044	0.98
LCA5L	Lebercilin	Protein Co	33	GC21M039	0.98
ZNF512	Zinc Finge	Protein Co	33	GC02P027	0.98
CREBRF	CREB3 Reg	Protein Co	32	GC05P173	0.98
EMSY	EMSY Trar	Protein Co	32	GC11P076	0.98
GRID2IP	Grid2 Inter	Protein Co	32	GC07M006	0.98
CPTP	Ceramide-	Protein Co	32	GC01P001	0.98
HEATR3	HEAT Rep	Protein Co	32	GC16P050	0.98
DCST2	DC-STAM	Protein Co	32	GC01M155	0.98
MTERF4	Mitochonc	Protein Co	32	GC02M241	0.98
OSGIN2	Oxidative	Protein Co	32	GC08P089	0.98
CFAP70	Cilia And	Protein Co	32	GC10M073	0.98
CFAP298	Cilia And	Protein Co	32	GC21M032	0.98
CDADC1	Cytidine A	Protein Co	32	GC13P049	0.98
CCDC36	Coiled-Co	Protein Co	32	GC03P049	0.98
C1orf189	Chromoso	Protein Co	32	GC01M154	0.98
FAXDC2	Fatty Acid	Protein Co	32	GC05M154	0.98
BOLA2	BolA Famil	Protein Co	32	GC16M029	0.98
ZMAT5	Zinc Finge	Protein Co	32	GC22M029	0.98
VWA7	Von Willet	Protein Co	32	GC06M032	0.98
WDR78	WD Repea	Protein Co	32	GC01M066	0.98
WHRN	Whirlin	Protein Co	32	GC09M114	0.98
PFN3	Profilin 3	Protein Co	32	GC05M177	0.98
SLC45A1	Solute Car	Protein Co	32	GC01P008	0.98
SYS1	SYS1 Golg	Protein Co	32	GC20P045	0.98
LINGO4	Leucine Ri	Protein Co	32	GC01M151	0.98
PLEKHN1	Pleckstrin	Protein Co	31	GC01P000	0.98
RASL11A	RAS Like F	Protein Co	31	GC13P027	0.98
TMEM89	Transmem	Protein Co	31	GC03M048	0.98
CNOT11	CCR4-NO	Protein Co	31	GC02P101	0.98
SEPTIN1	Septin 1	Protein Co	31	GC16M030	0.98
SEPTIN8	Septin 8	Protein Co	31	GC05M132	0.98
DUSP28	Dual Speci	Protein Co	31	GC02P240	0.98
SAP25	Sin3A Ass	Protein Co	31	GC07M100	0.98

MFSD4B	Major Faci	Protein Co	31	GC06P111	0.98
CCDC71	Coiled-Co	Protein Co	31	GC03M049	0.98
CCDC82	Coiled-Co	Protein Co	31	GC11M096	0.98
BPIFA3	BPI Fold C	Protein Co	31	GC20P033	0.98
ZNF774	Zinc Finge	Protein Co	31	GC15P090	0.98
NSG2	Neuronal '	Protein Co	30	GC05P174	0.98
PCP4L1	Purkinje C	Protein Co	30	GC01P161	0.98
CNOT9	CCR4-NO	Protein Co	30	GC02P218	0.98
DNLZ	DNL-Type	Protein Co	30	GC09M136	0.98
CATIP	Ciliogenes	Protein Co	30	GC02P218	0.98
AQP12A	Aquaporin	Protein Co	30	GC02P240	0.98
AQP12B	Aquaporin	Protein Co	30	GC02M240	0.98
TSGA10IP	Testis Spe	Protein Co	30	GC11P065	0.98
PRM3	Protamine	Protein Co	29	GC16M011	0.98
DEXI	Dexi Homo	Protein Co	29	GC16M010	0.98
C17orf67	Chromoso	Protein Co	29	GC17M056	0.98
TMA7	Translatior	Protein Co	29	GC03P048	0.98
ZNRD2	Zinc Ribbo	Protein Co	29	GC11P065	0.98
TMEM116	Transmem	Protein Co	28	GC12M111	0.98
PRXL2A	Peroxiredo	Protein Co	28	GC10P083	0.98
LY6G5C	Lymphocy	Protein Co	28	GC06M031	0.98
EIPR1	EARP Com	Protein Co	28	GC02M003	0.98
OR5B12	Olfactory I	Protein Co	28	GC11M058	0.98
OR5B21	Olfactory I	Protein Co	28	GC11M058	0.98
CFAP126	Cilia And F	Protein Co	28	GC01M161	0.98
CCDC184	Coiled-Co	Protein Co	28	GC12P048	0.98
ABRAXAS2	Abraxas 2,	Protein Co	28	GC10P124	0.98
AGAP5	ArfGAP Wi	Protein Co	28	GC10M073	0.98
FAM205A	Family Wit	Protein Co	28	GC09M034	0.98
C11orf21	Chromoso	Protein Co	28	GC11M002	0.98
LYRM9	LYR Motif	Protein Co	28	GC17M027	0.98
ANKRD65	Ankyrin Re	Protein Co	28	GC01M001	0.98
KRTAP5-5	Keratin As	Protein Co	28	GC11P001	0.98
ZUP1	Zinc Finge	Protein Co	28	GC06M116	0.98
PCNX3	Pecanex 3	Protein Co	27	GC11P065	0.98
LY6G6E	Lymphocy	Pseudoger	27	GC06M032	0.98
MUCL3	Mucin Like	Protein Co	27	GC06P033	0.98
RUSC1-AS	RUSC1 An	RNA Gene	27	GC01M155	0.98
MFSD13A	Major Faci	Protein Co	27	GC10P102	0.98
BICRA	BRD4 Inter	Protein Co	27	GC19P047	0.98
TRAPPC3L	Trafficking	Protein Co	27	GC06M116	0.98
KRTAP5-6	Keratin As	Protein Co	27	GC11P001	0.98
LCE3A	Late Corni	Protein Co	27	GC01M152	0.98
LCE3B	Late Corni	Protein Co	27	GC01P152	0.98
KHDC4	KH Domai	Protein Co	26	GC01M155	0.98
INKA1	Inka Box A	Protein Co	26	GC03P049	0.98
EEF1AKMT	EEF1A Lysi	Protein Co	26	GC10M124	0.98
OR5B2	Olfactory I	Protein Co	26	GC11M059	0.98
C9orf163	Chromoso	Protein Co	26	GC09P136	0.98
IGF2-AS	IGF2 Antis	RNA Gene	25	GC11P002	0.98
INAFM1	InaF Motif	Protein Co	25	GC19P047	0.98
STIMATE	STIM Activ	Protein Co	25	GC03M052	0.98
ARIH2OS	ARIH2 Op	RNA Gene	25	GC03M048	0.98
TTLL8	Tubulin Ty	Protein Co	25	GC22M050	0.98
TMEM250	Transmem	Protein Co	24	GC09M136	0.98
C1QTNF12	C1q And T	Protein Co	24	GC01M001	0.98
C20orf203	Chromoso	Protein Co	24	GC20M032	0.98
TNXA	Tenascin X	Pseudoger	24	GC06M032	0.98

LINC0269[illegible]	Long Inter	RNA Gene	24	GC15P038	0.98
PI4KAP2	Phosphati[illegible]	Pseudoger	24	GC22M02[illegible]	0.98
TSBP1	Testis Expr	Protein Co	23	GC06M03[illegible]	0.98
DELEC1	Deleted In	RNA Gene	22	GC09P114	0.98
GABARAPI	GABA Typ[illegible]	Pseudoger	22	GC15M09[illegible]	0.98
MEIKIN	Meiotic Kir	Protein Co	22	GC05M13[illegible]	0.98
C3orf84	Chromoso	Protein Co	21	GC03M04[illegible]	0.98
CALHM4	Calcium H	Protein Co	21	GC06P116	0.98
MIR219A1	MicroRNA	RNA Gene	20	GC06P033	0.98
SCARNA1[illegible]	Small Caja	RNA Gene	20	GC12M00[illegible]	0.98
ATP6V1G2	ATP6V1G2	RNA Gene	19	GC06M03[illegible]	0.98
STIMATE-I	STIMATE-I	Protein Co	18	GC03M05[illegible]	0.98
HCG4	HLA Comp	RNA Gene	18	GC06M03[illegible]	0.98
GVQW3	GVQW Mc	Protein Co	17	GC11P076	0.98
MSH5-SAI	MSH5-SAI	RNA Gene	17	GC06P033	0.98
HOXA10-[illegible]	HOXA10 A	RNA Gene	17	GC07P027	0.98
SCARNA1[illegible]	Small Caja	RNA Gene	17	GC12P006	0.98
ZFP91-CN	ZFP91-CN	RNA Gene	17	GC11P058	0.98
LINC0162[illegible]	Long Inter	RNA Gene	17	GC20M04[illegible]	0.98
TEX41	Testis Expr	RNA Gene	16	GC02P144	0.98
PPT2-EGFI	PPT2-EGFI	RNA Gene	16	GC06P033	0.98
JAZF1-AS[illegible]	JAZF1 Anti	RNA Gene	16	GC07P028	0.98
JMJD1C-A	JMJD1C A[illegible]	RNA Gene	16	GC10P063	0.98
MIR3909	MicroRNA	RNA Gene	16	GC22P035	0.98
SCARNA1[illegible]	Small Caja	RNA Gene	16	GC12M00[illegible]	0.98
MIR1301	MicroRNA	RNA Gene	16	GC02M02[illegible]	0.98
MIR1260B	MicroRNA	RNA Gene	16	GC11P096	0.98
GBAP1	Glucosylce	Pseudoger	16	GC01M15[illegible]	0.98
ACTA2-AS	ACTA2 An	RNA Gene	16	GC10P088	0.98
FLG-AS1	FLG Antise	RNA Gene	16	GC01P152	0.98
IL21-AS1	IL21 Antise	RNA Gene	15	GC04P122	0.98
SNORD16	Small Nucl	RNA Gene	15	GC15M06[illegible]	0.98
CD27-AS1	CD27 Anti[illegible]	RNA Gene	15	GC12M00[illegible]	0.98
ADAM1A	ADAM Me	Pseudoger	15	GC12P111	0.98
FAM99B	Family Wit	RNA Gene	15	GC11M00[illegible]	0.98
THAP7-AS	THAP7 An	RNA Gene	14	GC22P021	0.98
SLC26A4-[illegible]	SLC26A4 [illegible]	RNA Gene	14	GC07M10[illegible]	0.98
SUGT1P3	SUGT1 Pse	Pseudoger	14	GC13M04[illegible]	0.98
MIR647	MicroRNA	RNA Gene	14	GC20M06[illegible]	0.98
HCG17	HLA Comp	RNA Gene	14	GC06M03[illegible]	0.98
SATB1-AS	SATB1 Ant	RNA Gene	14	GC03P018	0.98
FAM205BF	Family Wit	Pseudoger	14	GC09M03[illegible]	0.98
C1RL-AS1	C1RL Antis	RNA Gene	14	GC12P007	0.98
C20orf181	Chromoso	Uncategor	14	GC20M06[illegible]	0.98
LINC0048[illegible]	Long Inter	RNA Gene	14	GC09P091	0.98
LINC0099[illegible]	Long Inter	RNA Gene	14	GC10P037	0.98
LINC0118[illegible]	Long Inter	RNA Gene	14	GC02M06[illegible]	0.98
ZSWIM8-[illegible]	ZSWIM8 A	RNA Gene	14	GC10M07[illegible]	0.98
IFITM4P	Interferon	Pseudoger	13	GC06M03[illegible]	0.98
SNORD12[illegible]	Small Nucl	RNA Gene	13	GC17M04[illegible]	0.98
RBFADN	RBFA Dow	RNA Gene	13	GC18P080	0.98
IGLV5-52	Immunogl	Protein Co	13	GC22P022	0.98
MMP24OS	MMP24 O	Protein Co	13	GC20M03[illegible]	0.98
NALT1	NOTCH1 [illegible]	RNA Gene	13	GC09P136	0.98
CRTC3-AS	CRTC3 An	RNA Gene	13	GC15M09[illegible]	0.98
MIR4686	MicroRNA	RNA Gene	13	GC11P002	0.98
MIR4425	MicroRNA	RNA Gene	13	GC01P025	0.98
HORMAD[illegible]	HORMAD[illegible]	RNA Gene	13	GC22M03[illegible]	0.98

DKFZP434	Uncharact	RNA Gene	13	GC09M13(	0.98
MIR1208	MicroRNA	RNA Gene	13	GC08P128	0.98
CATIP-AS1	CATIP Ant	RNA Gene	13	GC02M218	0.98
LURAP1L-	LURAP1L /	RNA Gene	13	GC09M012	0.98
FLJ31104	Uncharact	RNA Gene	13	GC05P055	0.98
ARHGEF38	ARHGEF38	RNA Gene	13	GC04P105	0.98
ASH1L-AS	ASH1L Ant	RNA Gene	13	GC01P155	0.98
TNRC6C-/	TNRC6C A	RNA Gene	13	GC17M078	0.98
WAKMAR2	Wound Ar	RNA Gene	13	GC06M13	0.98
ZBTB46-A	ZBTB46 Ar	RNA Gene	13	GC20P063	0.98
LINC00824	Long Inter	RNA Gene	13	GC08M128	0.98
LINC0143(	Long Inter	RNA Gene	13	GC20M044	0.98
LINC0122(	Long Inter	RNA Gene	13	GC14P075	0.98
LINC0125(	Long Inter	RNA Gene	13	GC02M002	0.98
ENSG00000244255		Protein Co	12	GC06P031	0.98
PDCL3P4	PDCL3 Pse	Pseudoger	12	GC03P101	0.98
OVOL1-AS	OVOL1 Ar	RNA Gene	12	GC11M065	0.98
TET2-AS1	TET2 Antis	RNA Gene	12	GC04M105	0.98
MIR623	MicroRNA	RNA Gene	12	GC13P099	0.98
HCG21	HLA Comp	RNA Gene	12	GC06M03(	0.98
MIR4673	MicroRNA	RNA Gene	12	GC09M13(	0.98
LOC10192	Uncharact	RNA Gene	12	GC05P172	0.98
CDC37P1	Cell Divisic	Pseudoger	12	GC16M028	0.98
ETS1-AS1	ETS1 Antis	RNA Gene	12	GC11P128	0.98
FAM238C	Family Wit	RNA Gene	12	GC10M02(	0.98
TNFRSF14	TNFRSF14	RNA Gene	12	GC01M002	0.98
LINC00548	Long Inter	RNA Gene	12	GC13M04(	0.98
LINC00581	Long Inter	RNA Gene	12	GC06M021	0.98
SMG1P5	SMG1 Pse	Pseudoger	12	GC16M03(	0.98
LINC00892	Long Inter	RNA Gene	12	GC0XP136	0.98
LINC0280(	Long Inter	RNA Gene	12	GC01P024	0.98
LINC02098	Long Inter	RNA Gene	12	GC11P128	0.98
MIR6090	MicroRNA	RNA Gene	11	GC11P128	0.98
NRAD1	Non-Codi	RNA Gene	11	GC13P043	0.98
LOC10192	Uncharact	RNA Gene	11	GC02P065	0.98
BSN-DT	BSN Diver	RNA Gene	11	GC03M049	0.98
MIR6513	MicroRNA	RNA Gene	11	GC02M218	0.98
MIR3936H	MIR3936 H	RNA Gene	11	GC05M132	0.98
CCR5AS	CCR5 Antis	RNA Gene	11	GC03M04(	0.98
UBE2Q1-A	UBE2Q1 A	RNA Gene	11	GC01P154	0.98
LINC02202	Long Inter	RNA Gene	11	GC05P159	0.98
SNORA58I	Small Nucl	RNA Gene	10	GC01P154	0.98
IRS3P	Insulin Rec	Pseudoger	10	GC07P100	0.98
SLC12A9-/	SLC12A9 /	RNA Gene	10	GC07M10(	0.98
IL6R-AS1	IL6R Antise	RNA Gene	10	GC01M154	0.98
ENSG00000249624		Protein Co	10	GC21P033	0.98
MIR6771	MicroRNA	RNA Gene	10	GC16P050	0.98
MIR4456	MicroRNA	RNA Gene	10	GC05M00(	0.98
DDR1-DT	DDR1 Dive	RNA Gene	10	GC06M03(	0.98
LOC10192	Uncharact	RNA Gene	10	GC10M08(	0.98
MHENCR	Melanoma	RNA Gene	10	GC20P063	0.98
RPL7P32	Ribosomal	Pseudoger	10	GC07P108	0.98
LOC10192	Uncharact	Protein Co	10	GC01P154	0.98
CCND3P1	Cyclin D3 I	Pseudoger	10	GC10M03(	0.98
ETF1P1	Eukaryotic	Pseudoger	10	GC06P033	0.98
ANKRD33I	ANKRD33I	RNA Gene	10	GC05M01(	0.98
LINC01845	Long Inter	RNA Gene	10	GC05M159	0.98
LINC01989	Long Inter	RNA Gene	10	GC17M034	0.98

LINC0255!	Long Inter	RNA Gene	10	GC12M04(	0.98
LINC0257(	Long Inter	RNA Gene	10	GC06M03(	0.98
ENSG00000263020		Protein Co	10	GC06P033	0.98
LINC0114	Long Inter	RNA Gene	10	GC14M08	0.98
RANP1	RAN Pseu	Pseudoger	10	GC06P033	0.98
ZBTB11-A	ZBTB11 Ar	RNA Gene	10	GC03P101	0.98
LINC0242	Long Inter	RNA Gene	10	GC12M06	0.98
ENSG00000254295		RNA Gene	9	GC05P172	0.98
ENSG00000236039		Uncategor	9	GC07M01	0.98
ENSG00000238160		RNA Gene	9	GC05M13	0.98
ENSG00000238290		RNA Gene	9	GC01P008	0.98
ENSG00000245156		RNA Gene	9	GC11M06(	0.98
IL1R1-AS1	IL1R1 Anti	RNA Gene	9	GC02M10	0.98
SUMO2P1	SUMO2 Ps	Pseudoger	9	GC06M02	0.98
ENSG00000253736		RNA Gene	9	GC05P172	0.98
EIF2S2P3	Eukaryotic	Pseudoger	9	GC10M09	0.98
ENSG00000229694		Uncategor	9	GC09P091	0.98
MICB-DT	MICB Dive	RNA Gene	9	GC06M03	0.98
RNA5SP1	RNA, 5S Ri	Pseudoger	9	GC05P132	0.98
RPS19P3	Ribosomal	Pseudoger	9	GC14M03	0.98
RNY4P10	RNY4 Pseu	Pseudoger	9	GC06P033	0.98
RPL23AP1	Ribosomal	Pseudoger	9	GC21P039	0.98
RNU7-57F	RNA, U7 S	Pseudoger	9	GC01P154	0.98
RPS5P3	Ribosomal	Pseudoger	9	GC21M03	0.98
ENSG00000229990		RNA Gene	9	GC10P073	0.98
LOC10192	Uncharact	RNA Gene	9	GC01P160	0.98
ABHD17AI	Abhydrola	Pseudoger	9	GC22M02(	0.98
ENSG00000258539		Protein Co	9	GC10M12	0.98
LINC0270	Long Inter	RNA Gene	9	GC11P001	0.98
ENSG00000260302		RNA Gene	9	GC18M01	0.98
ZNF90P1	Zinc Finge	Pseudoger	9	GC03P101	0.98
LINC0060	Long Inter	RNA Gene	9	GC05P040	0.98
ENSG00000253508		RNA Gene	9	GC07M02	0.98
LINC0221	Long Inter	RNA Gene	9	GC05M01(	0.98
RNU6-344	RNA, U6 S	Pseudoger	8	GC06P106	0.98
ENSG00000227938		RNA Gene	8	GC02M02	0.98
SPTLC1P1	Serine Palr	Pseudoger	8	GC10P031	0.98
ENSG00000248753		RNA Gene	8	GC05P135	0.98
ENSG00000241764		Uncategor	8	GC07M10	0.98
ENSG00000238280		RNA Gene	8	GC10M06	0.98
ENSG00000234630		RNA Gene	8	GC22P023	0.98
ENSG00000247121		RNA Gene	8	GC05M09	0.98
ENSG00000248373		RNA Gene	8	GC04P104	0.98
SNRPGP7	Small Nucl	Pseudoger	8	GC02M02	0.98
ENSG00000243696		Protein Co	8	GC03M05	0.98
ENSG00000253445		RNA Gene	8	GC05M17	0.98
ENSG00000253111		RNA Gene	8	GC08P125	0.98
ENSG00000234261		RNA Gene	8	GC06M01	0.98
ENSG00000234290		RNA Gene	8	GC05M13	0.98
ENSG00000234117		RNA Gene	8	GC06P116	0.98
ENSG00000234132		RNA Gene	8	GC01P201	0.98
ENSG00000254855		RNA Gene	8	GC11M06(	0.98
ENSG00000255038		RNA Gene	8	GC11M06(	0.98
ENSG00000254461		RNA Gene	8	GC11M06(	0.98
ENSG00000255135		RNA Gene	8	GC11M07(	0.98
ENSG00000255320		RNA Gene	8	GC11M06(	0.98
ENSG00000254810		RNA Gene	8	GC11M07(	0.98
HMGB3P4	High Mob	Pseudoger	8	GC13P099	0.98

HNRNPCP	Heteroger	Pseudoger	8	GC16P011	0.98
RNU1-134	RNA, U1 S	Pseudoger	8	GC20P063	0.98
EBAG9P1	EBAG9 Pse	Pseudoger	8	GC10M099	0.98
RN7SL391	RNA, 7SL,	Pseudoger	8	GC12P006	0.98
ENSG00000231128		RNA Gene	8	GC01P113	0.98
ENSG00000231355		RNA Gene	8	GC21M033	0.98
ENSG00000230534		RNA Gene	8	GC10M035	0.98
ENSG00000230684		RNA Gene	8	GC09P129	0.98
SERBP1P3	SERPINE1	Pseudoger	8	GC03M053	0.98
GLULP4	Glutamate	Pseudoger	8	GC09P034	0.98
RNA5SP18	RNA, 5S Ri	Pseudoger	8	GC05M056	0.98
DPPA5P4	Developm	Pseudoger	8	GC14M075	0.98
ENSG00000226681		RNA Gene	8	GC02M181	0.98
NPM1P17	Nucleopho	Pseudoger	8	GC03P137	0.98
ENSG00000226812		RNA Gene	8	GC20M044	0.98
ENSG00000225421		RNA Gene	8	GC02M198	0.98
ENSG00000212228		RNA Gene	8	GC16M011	0.98
RNU6-43P	RNA, U6 S	Pseudoger	8	GC10P102	0.98
RNU6-474	RNA, U6 S	Pseudoger	8	GC02P203	0.98
RNU6-543	RNA, U6 S	Pseudoger	8	GC10P063	0.98
RNU6-638	RNA, U6 S	Pseudoger	8	GC17M078	0.98
ENSG00000199550		RNA Gene	8	GC11P002	0.98
RNU6-70P	RNA, U6 S	Pseudoger	8	GC13M027	0.98
ENSG00000224228		RNA Gene	8	GC01P172	0.98
ENSG00000224478		RNA Gene	8	GC06P159	0.98
ENSG00000224934		RNA Gene	8	GC10P099	0.98
ENSG00000225172		RNA Gene	8	GC01P198	0.98
RPL21P33	Ribosomal	Pseudoger	8	GC02P060	0.98
RNU6-850	RNA, U6 S	Pseudoger	8	GC06M031	0.98
RNU6-919	RNA, U6 S	Pseudoger	8	GC20P049	0.98
ENSG00000204758		Uncategor	8	GC05M172	0.98
RPSAP64	Ribosomal	Pseudoger	8	GC21P038	0.98
ENSG00000228778		RNA Gene	8	GC10P099	0.98
ENSG00000228863		RNA Gene	8	GC01P160	0.98
ENSG00000228412		Uncategor	8	GC06M019	0.98
ENSG00000229299		RNA Gene	8	GC20P063	0.98
CCT5P2	Chaperoni	Pseudoger	8	GC13P078	0.98
CCR12P	C-C Motif	Pseudoger	8	GC13M099	0.98
CBX3P9	Chromobo	Pseudoger	8	GC06M116	0.98
ENSG00000284779		Protein Co	8	GC11M002	0.98
ENSG00000285551		Protein Co	8	GC10P062	0.98
BTF3P2	BTF3 Pseu	Pseudoger	8	GC14M028	0.98
LOC11226	Uncharact	Protein Co	8	GC06M159	0.98
ENSG00000255966		RNA Gene	8	GC12M006	0.98
ENSG00000256967		RNA Gene	8	GC12M007	0.98
KRT18P39	Keratin 18	Pseudoger	8	GC02M203	0.98
ENSG00000259005		RNA Gene	8	GC14M074	0.98
ENSG00000258407		RNA Gene	8	GC14M088	0.98
ENSG00000258559		RNA Gene	8	GC14M074	0.98
ENSG00000258646		RNA Gene	8	GC14M075	0.98
ENSG00000258740		RNA Gene	8	GC14M075	0.98
ENSG00000258820		RNA Gene	8	GC14P075	0.98
ENSG00000261338		RNA Gene	8	GC02P218	0.98
LINC02354	Long Inter	RNA Gene	8	GC12P047	0.98
LINC02723	Long Inter	RNA Gene	8	GC11M064	0.98
ENSG00000260257		RNA Gene	8	GC20P032	0.98
ENSG00000256433		RNA Gene	8	GC12P006	0.98
TRG-TCC2	TRNA-Gly	RNA Gene	8	GC01P161	0.98

ENSG00000259202		RNA Gene	8	GC15M06	0.98
TRL-CAG1	TRNA-Leu	RNA Gene	8	GC01M16	0.98
ENSG00000236471		RNA Gene	8	GC21P015	0.98
ENSG00000235888		RNA Gene	8	GC21M03	0.98
ENSG00000261573		RNA Gene	8	GC01M19	0.98
ENSG00000270640		RNA Gene	8	GC02P028	0.98
ENSG00000271855		RNA Gene	8	GC02P009	0.98
ENSG00000271781		RNA Gene	8	GC05P000	0.98
ENSG00000272305		Protein Co	8	GC03M05	0.98
ENSG00000262151		RNA Gene	8	GC16M01	0.98
ENSG00000263080		Uncategor	8	GC16M01	0.98
ENSG00000263033		RNA Gene	8	GC16P011	0.98
ENSG00000263766		RNA Gene	8	GC17M04	0.98
ENSG00000261367		RNA Gene	8	GC16P030	0.98
ENSG00000273154		Protein Co	8	GC20P063	0.98
ENSG00000266202		Protein Co	8	GC17M02	0.98
ENSG00000267199		RNA Gene	8	GC18P013	0.98
ENSG00000267480		RNA Gene	8	GC18M01	0.98
ENSG00000268746		RNA Gene	8	GC19P047	0.98
ENSG00000268810		RNA Gene	8	GC19M04	0.98
ENSG00000269514		RNA Gene	8	GC12P048	0.98
ENSG00000269621		RNA Gene	8	GC01P151	0.98
ENSG00000269919		RNA Gene	8	GC06P106	0.98
ENSG00000270124		RNA Gene	8	GC16M08	0.98
ENSG00000270210		RNA Gene	8	GC02P028	0.98
CICP4	Capicua Tr	Pseudoge	8	GC20M06	0.98
RPS21P8	Ribosomal	Pseudoge	7	GC13P026	0.98
RPS23P10	Ribosomal	Pseudoge	7	GC01M16	0.98
RNU6-32	RNA, U6 S	Pseudoge	7	GC0XP136	0.98
RNU6-351	RNA, U6 S	Pseudoge	7	GC04P104	0.98
PEBP1P3	Phosphati	Pseudoge	7	GC01M19	0.98
ENSG00000248734		RNA Gene	7	GC05P096	0.98
ENSG00000238326		RNA Gene	7	GC05M05	0.98
LOC10537	Uncharact	RNA Gene	7	GC06P159	0.98
ENSG00000249743		RNA Gene	7	GC05P073	0.98
RN7SKP22	RN7SK Ps	Pseudoge	7	GC08P128	0.98
ENSG00000232124		RNA Gene	7	GC21M04	0.98
RNU6-29	RNA, U6 S	Pseudoge	7	GC05P056	0.98
RPLP0P7	Ribosomal	Pseudoge	7	GC02M15	0.98
ENSG00000184441		RNA Gene	7	GC21P044	0.98
ENSG00000199332		RNA Gene	7	GC06M03	0.98
ENSG00000199473		RNA Gene	7	GC07M01	0.98
RPL5P26	Ribosomal	Pseudoge	7	GC10P069	0.98
ENSG00000219159		Uncategor	7	GC02P240	0.98
RNU6-92	RNA, U6 S	Pseudoge	7	GC08M08	0.98
ENSG00000202533		RNA Gene	7	GC05P132	0.98
ENSG00000205537		RNA Gene	7	GC12P047	0.98
ENSG00000283782		Protein Co	7	GC05P132	0.98
ENSG00000284633		RNA Gene	7	GC22P039	0.98
FGFR1OP	FGFR1 On	Pseudoge	7	GC13P026	0.98
ENSG00000283321		Protein Co	7	GC07P017	0.98
AIMP1P2	Aminoacyl	Pseudoge	7	GC01M17	0.98
LINC0195	Long Inter	RNA Gene	7	GC02P156	0.98
ENSG00000260651		RNA Gene	7	GC04M10	0.98
ENSG00000260773		RNA Gene	7	GC15M06	0.98
ENSG00000260577		RNA Gene	7	GC16M06	0.98
ENSG00000260233		Uncategor	7	GC11M06	0.98
ENSG00000259314		RNA Gene	7	GC15M09	0.98

ENSG00000235434		RNA Gene	7	GC01P019	0.98
ENSG00000234929		RNA Gene	7	GC02M002	0.98
ENSG00000272109		RNA Gene	7	GC05P096	0.98
ENSG00000261644		RNA Gene	7	GC16M050	0.98
ENSG00000263893		RNA Gene	7	GC17P072	0.98
ENSG00000263756		RNA Gene	7	GC06M032	0.98
ENSG00000267303		Protein Co	7	GC19M010	0.98
ENSG00000269570		RNA Gene	7	GC11M058	0.98
ENSG00000270000		RNA Gene	7	GC14M074	0.98
ENSG00000270212		RNA Gene	7	GC19M033	0.98
LINC02863	Long Inter	RNA Gene	7	GC05M132	0.98
lnc-ITGB1BP1-3		RNA Gene	6	GC02M009	0.98
LOC10192	Uncharacte	RNA Gene	6	GC21M015	0.98
ENSG00000251049		RNA Gene	6	GC04M057	0.98
ENSG00000253955		Uncategor	6	GC05P173	0.98
ENSG00000237773		RNA Gene	6	GC07M016	0.98
POLR2LP1	RNA Polyr	Pseudoger	6	GC06P033	0.98
hsa-miR-5096-095		RNA Gene	6	GC07M028	0.98
LOC10536	Uncharacte	RNA Gene	6	GC21M015	0.98
ENSG00000247853		RNA Gene	6	GC12P006	0.98
SNRPGP8	Small Nucl	Pseudoger	6	GC02M227	0.98
ENSG00000250993		RNA Gene	6	GC04M179	0.98
ENSG00000251459		RNA Gene	6	GC04P057	0.98
KCCAT333	Renal Clea	RNA Gene	6	GC07P017	0.98
HMGN2P1	High Mobi	Pseudoger	6	GC01P155	0.98
lnc-GCA-5		RNA Gene	6	GC02P162	0.98
RNU6-144	RNA, U6 S	Pseudoger	6	GC08P137	0.98
EIF1P7	Eukaryotic	Pseudoger	6	GC02M009	0.98
lnc-TMED10-5		RNA Gene	6	GC14M075	0.98
lnc-TMEM50B-3		RNA Gene	6	GC21M033	0.98
RN7SKP11	RN7SK Pse	Pseudoger	6	GC04M004	0.98
RN7SL51P	RNA, 7SL,	Pseudoger	6	GC02M062	0.98
RN7SKP21	RN7SK Pse	Pseudoger	6	GC06P105	0.98
ENSG00000231557		RNA Gene	6	GC02M198	0.98
ENSG00000230074		RNA Gene	6	GC09P034	0.98
ENSG00000230537		RNA Gene	6	GC09M091	0.98
RNU6-222	RNA, U6 S	Pseudoger	6	GC19M054	0.98
NIPA2P5	NIPA2 Pse	Pseudoger	6	GC13P079	0.98
LOC10012	Eukaryotic	Pseudoger	6	GC01P008	0.98
lnc-IL10-5		RNA Gene	6	GC01M206	0.98
ENSG00000225931		Uncategor	6	GC01P002	0.98
ENSG00000226032		RNA Gene	6	GC06M159	0.98
ENSG00000197254		Pseudoger	6	GC11P058	0.98
ENSG00000197536		Uncategor	6	GC05P132	0.98
RNU6-704	RNA, U6 S	Pseudoger	6	GC01M200	0.98
RPL35P9	Ribosomal	Pseudoger	6	GC13P106	0.98
ENSG00000219410		RNA Gene	6	GC12P006	0.98
lnc-HOXA13-1		RNA Gene	6	GC07M027	0.98
ENSG00000222701		RNA Gene	6	GC16P030	0.98
ENSG00000200677		RNA Gene	6	GC15M090	0.98
RNU7-15P	RNA, U7 S	Pseudoger	6	GC05M132	0.98
ENSG00000228037		Uncategor	6	GC01P002	0.98
ENSG00000228430		Pseudoger	6	GC09P082	0.98
lnc-ARHGAP20-52		RNA Gene	6	GC11M110	0.98
ENSG00000286503		RNA Gene	6	GC05P108	0.98
lnc-ASIC2-2		RNA Gene	6	GC17M034	0.98
lnc-PRDM1-1		RNA Gene	6	GC06P106	0.98
ENSG00000281883		Protein Co	6	GC13P043	0.98

ENSG00000284829		RNA Gene	6	GC06M032	0.98
ENSG00000285082		Protein Co	6	GC09P117	0.98
ENSG00000285413		RNA Gene	6	GC21M044	0.98
ENSG00000285446		Protein Co	6	GC06P116	0.98
LOC39031	Chromoso	Pseudoger	6	GC12M048	0.98
BTF3L4P3	Basic Trans	Pseudoger	6	GC06M137	0.98
ENSG00000287771		RNA Gene	6	GC02P102	0.98
lnc-SCNN1A-1		RNA Gene	6	GC12M006	0.98
lnc-SDF4-1		RNA Gene	6	GC01M001	0.98
ENSG00000258860		RNA Gene	6	GC14M035	0.98
LINC02300	Long Inter	RNA Gene	6	GC14M028	0.98
YBX1P5	Y-Box Binc	Pseudoger	6	GC05P072	0.98
ENSG00000261025		RNA Gene	6	GC01M024	0.98
KRT18P56	Keratin 18	Pseudoger	6	GC05P040	0.98
LOC10798	Uncharact	RNA Gene	6	GC11P076	0.98
PIGCP2	Phosphati	Pseudoger	6	GC07P107	0.98
ENSG00000237371		RNA Gene	6	GC20P064	0.98
ENSG00000237422		RNA Gene	6	GC09M091	0.98
ENSG00000236318		Uncategor	6	GC07P017	0.98
lnc-MAP3K7-3		RNA Gene	6	GC06M090	0.98
ENSG00000234789		RNA Gene	6	GC09P129	0.98
ENSG00000272072		RNA Gene	6	GC07M107	0.98
ENSG00000272459		RNA Gene	6	GC05P177	0.98
ENSG00000262020		RNA Gene	6	GC16M011	0.98
ENSG00000272791		RNA Gene	6	GC10P073	0.98
ENSG00000273055		RNA Gene	6	GC07P107	0.98
ENSG00000273176		RNA Gene	6	GC22M035	0.98
ENSG00000266527		RNA Gene	6	GC17P027	0.98
LINC02635	Long Inter	RNA Gene	6	GC10P034	0.98
ENSG00000272477		RNA Gene	6	GC03M018	0.98
lnc-TM9SF2-4		RNA Gene	5	GC13P099	0.98
lnc-TM9SF2-7		RNA Gene	5	GC13P099	0.98
piR-50893		RNA Gene	5	GC01P001	0.98
lnc-USP36-4		RNA Gene	5	GC17M078	0.98
lnc-ZCCHC24-7		RNA Gene	5	GC10M079	0.98
lnc-IRF1-7		RNA Gene	5	GC05M132	0.98
HSALNG0086605		RNA Gene	5	GC11P096	0.98
lnc-LIPG-7		RNA Gene	5	GC18P048	0.98
lnc-MRPL23-2		RNA Gene	5	GC11P002	0.98
ENSG00000242044-0		RNA Gene	5	GC06M032	0.98
lnc-MIEN1-1		RNA Gene	5	GC17M039	0.98
lnc-MMEL1-1		RNA Gene	5	GC01M002	0.98
JA662168		RNA Gene	5	GC06M032	0.98
lnc-NDFIP1-1		RNA Gene	5	GC05P142	0.98
piR-55650-032		RNA Gene	5	GC10M006	0.98
lnc-NEK7-4		RNA Gene	5	GC01P198	0.98
piR-37824		RNA Gene	5	GC02M102	0.98
ENSG00000234062		Pseudoger	5	GC0XP136	0.98
lnc-NFKBIZ-2		RNA Gene	5	GC03P101	0.98
lnc-NFKBIZ-3		RNA Gene	5	GC03P101	0.98
ENSG00000253683		Pseudoger	5	GC05M172	0.98
ENSG00000249650		Uncategor	5	GC05M000	0.98
lnc-MTPAP-7		RNA Gene	5	GC10M030	0.98
piR-33458		RNA Gene	5	GC15P072	0.98
lnc-CLEC16A-4		RNA Gene	5	GC16P011	0.98
piR-42694-019		RNA Gene	5	GC10P030	0.98
lnc-TH-1		RNA Gene	5	GC11M002	0.98
HNRNPA1	Heteroger	Pseudoger	5	GC09M004	0.98

lnc-TSPAN32-4	RNA Gene	5	GC11P002	0.98
piR-34911-011	RNA Gene	5	GC17M039	0.98
piR-43104-029	RNA Gene	5	GC19P001	0.98
lnc-TRRAP-5	RNA Gene	5	GC07P099	0.98
lnc-ZC3H12C-5	RNA Gene	5	GC11P110	0.98
HSALNG0021688	RNA Gene	5	GC02M203	0.98
lnc-DUSP1-7	RNA Gene	5	GC05M172	0.98
lnc-TNFRSF14-3	RNA Gene	5	GC01P002	0.98
lnc-TNFRSF1A-1	RNA Gene	5	GC12M006	0.98
lnc-TNFSF18-3	RNA Gene	5	GC01M172	0.98
piR-50444-308	RNA Gene	5	GC03P046	0.98
lnc-TMED10-2	RNA Gene	5	GC14M075	0.98
lnc-TMED10-4	RNA Gene	5	GC14M075	0.98
lnc-TMEM268-4	RNA Gene	5	GC09P114	0.98
lnc-TMEM17-10	RNA Gene	5	GC02M062	0.98
piR-51327	RNA Gene	5	GC12M111	0.98
lnc-FAM109A-1	RNA Gene	5	GC12M111	0.98
HSALNG0001696	RNA Gene	5	GC01P024	0.98
piR-51137-090	RNA Gene	5	GC04M105	0.98
piR-43105-342	RNA Gene	5	GC02M025	0.98
lnc-FAP-3	RNA Gene	5	GC02M162	0.98
lnc-USP25-6	RNA Gene	5	GC21P015	0.98
lnc-DDR1-4	RNA Gene	5	GC06P033	0.98
CHCHD2P Coiled-Co	Pseudogen	5	GC19P034	0.98
piR-50437-058	RNA Gene	5	GC10P062	0.98
lnc-VEGFA-1	RNA Gene	5	GC06P046	0.98
lnc-FCGR3A-2	RNA Gene	5	GC01M161	0.98
lnc-FCGR3A-4	RNA Gene	5	GC01M161	0.98
piR-52079-016	RNA Gene	5	GC01P113	0.98
piR-52079-043	RNA Gene	5	GC12P047	0.98
lnc-WASHC5-9	RNA Gene	5	GC08M127	0.98
lnc-FLI1-5	RNA Gene	5	GC11P128	0.98
HSALNG0017394	RNA Gene	5	GC02M102	0.98
lnc-FNBP1-2	RNA Gene	5	GC09M129	0.98
lnc-FOSL2-2	RNA Gene	5	GC02P028	0.98
lnc-EMC8-1	RNA Gene	5	GC16M085	0.98
lnc-UBAC2-4	RNA Gene	5	GC13P099	0.98
piR-59241	RNA Gene	5	GC15P072	0.98
ENSG00000206734	RNA Gene	5	GC17P047	0.98
LOC10050 Uncharact	RNA Gene	5	GC09M091	0.98
lnc-IL15RA-4	RNA Gene	5	GC10M006	0.98
LOC10053 ATP Bindir	Pseudogen	5	GC02P227	0.98
piR-44878-042	RNA Gene	5	GC07M100	0.98
ENSG00000213386	Pseudogen	5	GC05M172	0.98
NONHSAG001750.2	RNA Gene	5	GC01P067	0.98
NONHSAG003874.2	RNA Gene	5	GC01P198	0.98
NONHSAG041785.2	RNA Gene	5	GC05P142	0.98
piR-53431-298	RNA Gene	5	GC18P013	0.98
piR-45035-151	RNA Gene	5	GC05M096	0.98
ENSG00000224431	Pseudogen	5	GC05P132	0.98
ENSG00000224988	Pseudogen	5	GC09M129	0.98
lnc-HLA-A-2	RNA Gene	5	GC06P033	0.98
lnc-HOXA11-1	RNA Gene	5	GC07M027	0.98
lnc-HOXA11-3	RNA Gene	5	GC07M027	0.98
lnc-HOXA13-3	RNA Gene	5	GC07M027	0.98
NONHSAG007397.2	RNA Gene	5	GC11P001	0.98
HSALNG0046732	RNA Gene	5	GC05P173	0.98
lnc-GRB7-1	RNA Gene	5	GC17P039	0.98

lnc-GSDMC-13	RNA Gene	5	GC08M128	0.98
lnc-IL6ST-2	RNA Gene	5	GC05M056	0.98
NXPE2P1 Neurexoph	Pseudogen	5	GC11M114	0.98
RF00483	RNA Gene	5	GC11M002	0.98
lnc-CTNND2-10	RNA Gene	5	GC05M010	0.98
lnc-CREB5-4	RNA Gene	5	GC07P028	0.98
lnc-CNTF-2	RNA Gene	5	GC11P058	0.98
lnc-ATP6V1G3-6	RNA Gene	5	GC01M198	0.98
lnc-REL-2	RNA Gene	5	GC02P060	0.98
RF00017-5119	RNA Gene	5	GC05M173	0.98
ENSG00000286116	RNA Gene	5	GC10M088	0.98
lnc-ATG5-7	RNA Gene	5	GC06M105	0.98
RF00017-5399	RNA Gene	5	GC06M030	0.98
RF00017-5414	RNA Gene	5	GC06M032	0.98
AC003959	RNA Gene	5	GC05P132	0.98
lnc-ANKMY1-3	RNA Gene	5	GC02M240	0.98
ENSG00000283648	RNA Gene	5	GC07M148	0.98
lnc-PSORS1C2-1	RNA Gene	5	GC06M031	0.98
lnc-PTGIR-1	RNA Gene	5	GC19M046	0.98
lnc-PTGIR-2	RNA Gene	5	GC19M046	0.98
RF00017-4587	RNA Gene	5	GC05M038	0.98
lnc-APOBR-1	RNA Gene	5	GC16P028	0.98
RF00017-4625	RNA Gene	5	GC05P055	0.98
RF00017-4964	RNA Gene	5	GC05P142	0.98
ENSG00000285837	RNA Gene	5	GC10P062	0.98
lnc-PWP2-2	RNA Gene	5	GC21P044	0.98
lnc-ARFRP1-1	RNA Gene	5	GC20M063	0.98
lnc-CALM3-3	RNA Gene	5	GC19P046	0.98
ENSG00000287850	RNA Gene	5	GC20M032	0.98
lnc-SEH1L-5	RNA Gene	5	GC18P013	0.98
AY077737	RNA Gene	5	GC02P233	0.98
lnc-CCT4-2	RNA Gene	5	GC02M061	0.98
lnc-CEP76-3	RNA Gene	5	GC18M016	0.98
lnc-SLC34A1-3	RNA Gene	5	GC05P177	0.98
lnc-SNX13-5	RNA Gene	5	GC07M017	0.98
RF00017-5695	RNA Gene	5	GC06M105	0.98
lnc-RNF39-8	RNA Gene	5	GC06M030	0.98
piR-48877	RNA Gene	5	GC10P083	0.98
RF00017-6553	RNA Gene	5	GC07M107	0.98
lnc-SBNO2-2	RNA Gene	5	GC19M001	0.98
lnc-RTN4IP1-6	RNA Gene	5	GC06M106	0.98
lnc-RTTN-6	RNA Gene	5	GC18M069	0.98
lnc-C1QTNF1-9	RNA Gene	5	GC17P078	0.98
ENSG00000286974	RNA Gene	5	GC06P033	0.98
RF00017-3549	RNA Gene	5	GC20P032	0.98
lnc-AHR-4	RNA Gene	5	GC07P017	0.98
ENSG00000283265	RNA Gene	5	GC06P137	0.98
ENSG00000283286	RNA Gene	5	GC05P040	0.98
ENSG00000283360	RNA Gene	5	GC01M161	0.98
ENSG00000283573	RNA Gene	5	GC06P046	0.98
piR-48950-118	RNA Gene	5	GC03P046	0.98
RF00017-6464	RNA Gene	5	GC07M099	0.98
piR-57133-098	RNA Gene	5	GC12P008	0.98
ENSG00000287597	RNA Gene	5	GC05M040	0.98
RF00017-6488	RNA Gene	5	GC07M100	0.98
lnc-SCGB2B2-133	RNA Gene	5	GC19M034	0.98
lnc-SCGB2B2-4	RNA Gene	5	GC19M033	0.98
lnc-SCNN1A-2	RNA Gene	5	GC12M006	0.98

RF00017-717	RNA Gene	5	GC10P083	0.98
lnc-C9orf78-2	RNA Gene	5	GC09M129	0.98
piR-31937-163	RNA Gene	5	GC04P105	0.98
ENSG00000283579	RNA Gene	5	GC13M026	0.98
RF00017-5418	RNA Gene	5	GC06M032	0.98
piR-48759-287	RNA Gene	5	GC07P099	0.98
RF00017-3286	RNA Gene	5	GC02M102	0.98
RF00017-349	RNA Gene	5	GC01M151	0.98
AB372574	RNA Gene	5	GC01P151	0.98
lnc-OLIG3-1	RNA Gene	5	GC06M137	0.98
lnc-NXPE3-2	RNA Gene	5	GC03P101	0.98
RF00017-1621	RNA Gene	5	GC14M075	0.98
RF00017-1272	RNA Gene	5	GC12M111	0.98
L13712-019	RNA Gene	5	GC20P032	0.98
ENSG00000275693	RNA Gene	5	GC10P088	0.98
ENSG00000276609	RNA Gene	5	GC07P027	0.98
ENSG00000278708	RNA Gene	5	GC07P027	0.98
RF00017-2113	RNA Gene	5	GC16P068	0.98
ENSG00000260249	Uncategor	5	GC16P050	0.98
RF00017-2930	RNA Gene	5	GC19M033	0.98
RF00017-2425	RNA Gene	5	GC17P047	0.98
RF00017-2721	RNA Gene	5	GC19M001	0.98
ENSG00000236710	Pseudogen	5	GC11M003	0.98
ENSG00000271267	Pseudogen	5	GC01M155	0.98
ENSG00000271992	RNA Gene	5	GC01P070	0.98
lnc-PLEKHG6-4	RNA Gene	5	GC12P006	0.98
ENSG00000272644	RNA Gene	5	GC02M219	0.98
ENSG00000273466	RNA Gene	5	GC02M218	0.98
ENSG00000274038	RNA Gene	5	GC16P011	0.98
ENSG00000274737	RNA Gene	5	GC12M047	0.98
ENSG00000268069	RNA Gene	5	GC12P047	0.98
piR-58538-001	RNA Gene	4	GC16P085	0.98
piR-58538-016	RNA Gene	4	GC16P085	0.98
lnc-TRIB1-1	RNA Gene	4	GC08P125	0.98
piR-43107-300	RNA Gene	4	GC08M137	0.98
HSALNG0007877	RNA Gene	4	GC01M160	0.98
lnc-WNT4-6	RNA Gene	4	GC01M022	0.98
piR-52079-091	RNA Gene	4	GC18M069	0.98
lnc-IQCH-5	RNA Gene	4	GC15P071	0.98
lnc-ZNF365-3	RNA Gene	4	GC10P062	0.98
HSALNG0088853	RNA Gene	4	GC12P006	0.98
ENSG00000227836	Pseudogen	4	GC05P096	0.98
lnc-ITGB1BP1-2	RNA Gene	4	GC02M009	0.98
HSALNG0088095	RNA Gene	4	GC11M128	0.98
HSALNG0130695	RNA Gene	4	GC20M049	0.98
piR-30396	RNA Gene	4	GC15P072	0.98
piR-61945-308	RNA Gene	4	GC20P044	0.98
lnc-LYRM9-3	RNA Gene	4	GC17M027	0.98
ENSG00000237553	Pseudogen	4	GC09M005	0.98
piR-46391-002	RNA Gene	4	GC01P154	0.98
hsa-miR-5095-436	RNA Gene	4	GC06P137	0.98
ENSG00000242299	Pseudogen	4	GC03M101	0.98
ENSG00000240023	Pseudogen	4	GC14M035	0.98
ENSG00000238138	Pseudogen	4	GC03M015	0.98
piR-55186-001	RNA Gene	4	GC01P007	0.98
LOC10537 Uncharact	RNA Gene	4	GC02P162	0.98
ENSG00000244061	Pseudogen	4	GC05P072	0.98
piR-38319	RNA Gene	4	GC01P198	0.98

lnc-MMEL1-2	RNA Gene	4	GC01M002	0.98
piR-38259	RNA Gene	4	GC12M111	0.98
piR-55281-150	RNA Gene	4	GC13M099	0.98
piR-46501-010	RNA Gene	4	GC10P102	0.98
piR-45932-058	RNA Gene	4	GC02M198	0.98
piR-46002-152	RNA Gene	4	GC13M049	0.98
lnc-NBN-9	RNA Gene	4	GC08M089	0.98
piR-47234	RNA Gene	4	GC15P072	0.98
RF00001-253	RNA Gene	4	GC05M056	0.98
ENSG00000254926	Pseudogen	4	GC11M059	0.98
ENSG00000254755	Pseudogen	4	GC11P076	0.98
RF00001-209	RNA Gene	4	GC22P039	0.98
HG983680	RNA Gene	4	GC01P161	0.98
lnc-TRAPPC3L-2	RNA Gene	4	GC06M116	0.98
piR-52079-106	RNA Gene	4	GC02M062	0.98
lnc-ZMAT5-2	RNA Gene	4	GC22M029	0.98
piR-52294-068	RNA Gene	4	GC02M062	0.98
piR-43408-221	RNA Gene	4	GC07P107	0.98
lnc-DYRK2-15	RNA Gene	4	GC12P068	0.98
piR-43099-059	RNA Gene	4	GC10M079	0.98
RF00994-809	RNA Gene	4	GC05P010	0.98
RF00994-753	RNA Gene	4	GC04P102	0.98
lnc-TMEM258-1	RNA Gene	4	GC11M061	0.98
lnc-DSE-1	RNA Gene	4	GC06P116	0.98
lnc-DNLZ-1	RNA Gene	4	GC09M136	0.98
piR-51267-027	RNA Gene	4	GC07M017	0.98
piR-51449	RNA Gene	4	GC12P111	0.98
HQ292134	RNA Gene	4	GC01P161	0.98
piR-51137-089	RNA Gene	4	GC04M102	0.98
lnc-TTC33-6	RNA Gene	4	GC05M040	0.98
lnc-USP20-5	RNA Gene	4	GC09P129	0.98
LOC10272 Uncharact	RNA Gene	4	GC19P001	0.98
lnc-LAMB4-3	RNA Gene	4	GC07M107	0.98
piR-33804-078	RNA Gene	4	GC07P017	0.98
lnc-CYTL1-3	RNA Gene	4	GC04M004	0.98
piR-50437-360	RNA Gene	4	GC02M024	0.98
piR-58297-114	RNA Gene	4	GC12M055	0.98
piR-50346	RNA Gene	4	GC12M111	0.98
lnc-WASF3-4	RNA Gene	4	GC13P026	0.98
HSALNG0015265	RNA Gene	4	GC02P060	0.98
piR-43325-002	RNA Gene	4	GC01M019	0.98
piR-59907-003	RNA Gene	4	GC01P172	0.98
HSALNG0017398	RNA Gene	4	GC02M102	0.98
piR-61240-151	RNA Gene	4	GC02M191	0.98
piR-44610-008	RNA Gene	4	GC05P040	0.98
piR-44610-011	RNA Gene	4	GC06M032	0.98
NONHSAG017238.2	RNA Gene	4	GC15P072	0.98
lnc-IKZF1-5	RNA Gene	4	GC07P050	0.98
lnc-IL12B-2	RNA Gene	4	GC05M159	0.98
lnc-IL19-2	RNA Gene	4	GC01P206	0.98
lnc-HDAC11-4	RNA Gene	4	GC03P013	0.98
ENSG00000213440	Pseudogen	4	GC21M044	0.98
NONHSAG026080.2	RNA Gene	4	GC19P046	0.98
piR-43939-002	RNA Gene	4	GC01P007	0.98
lnc-GOT1-1	RNA Gene	4	GC10M099	0.98
NONHSAG043568.2	RNA Gene	4	GC06P033	0.98
HSALNG0073924	RNA Gene	4	GC09P114	0.98
NONHSAG045774.2	RNA Gene	4	GC06P033	0.98

lnc-ICAM3-1	RNA Gene	4	GC19M01(	0.98
piR-61101-576	RNA Gene	4	GC06P045	0.98
LOC10028 Mitochonc	Pseudoger	4	GC03M13	0.98
lnc-HLA-DQA1-9	RNA Gene	4	GC06P033	0.98
lnc-HLA-DRB1-7	RNA Gene	4	GC06M032	0.98
NONHSAG031883.2-	RNA Gene	4	GC20P046	0.98
NONHSAG031883.2-	RNA Gene	4	GC20P046	0.98
LOC10041 MIA SH3 D	Pseudoger	4	GC02P199	0.98
lnc-HNF4A-1	RNA Gene	4	GC20P044	0.98
ENSG00000220412	Pseudoger	4	GC06M13	0.98
piR-45012-401	RNA Gene	4	GC05M04(	0.98
NONHSAG008489.2	RNA Gene	4	GC11P061	0.98
HSALNG0043662	RNA Gene	4	GC05P096	0.98
piR-36455	RNA Gene	4	GC12M11	0.98
piR-52916-031	RNA Gene	4	GC06M15	0.98
HSALNG0050236	RNA Gene	4	GC06P046	0.98
ENSG00000227758	Pseudoger	4	GC06P033	0.98
piR-37170-040	RNA Gene	4	GC02M218	0.98
piR-61514-156	RNA Gene	4	GC06M032	0.98
lnc-CTIF-9	RNA Gene	4	GC18P048	0.98
RF00066-120	RNA Gene	4	GC20P049	0.98
piR-50308-096	RNA Gene	4	GC11M07	0.98
lnc-ARHGAP20-11	RNA Gene	4	GC11M11(	0.98
ENSG00000285616	RNA Gene	4	GC05M04(	0.98
lnc-R3HDML-1	RNA Gene	4	GC20P044	0.98
piR-56480-015	RNA Gene	4	GC12M11	0.98
ENSG00000286368	RNA Gene	4	GC06M02	0.98
lnc-ATP6V1G3-5	RNA Gene	4	GC01M198	0.98
piR-32461-022	RNA Gene	4	GC10M062	0.98
RF00017-5114	RNA Gene	4	GC05M172	0.98
ENSG00000286186	RNA Gene	4	GC18M07	0.98
RF00017-3998	RNA Gene	4	GC03P049	0.98
lnc-PRKCD-2	RNA Gene	4	GC03P053	0.98
lnc-AMZ1-1	RNA Gene	4	GC07P002	0.98
piR-39701-054	RNA Gene	4	GC06M09(	0.98
lnc-PSMA6-6	RNA Gene	4	GC14P035	0.98
RF00017-4375	RNA Gene	4	GC04P101	0.98
piR-56341-125	RNA Gene	4	GC02M198	0.98
piR-48325-110	RNA Gene	4	GC21M03	0.98
piR-48325-111	RNA Gene	4	GC21P039	0.98
lnc-PTCD2-5	RNA Gene	4	GC05P072	0.98
piR-56341-188	RNA Gene	4	GC05P135	0.98
lnc-PTGER4-8	RNA Gene	4	GC05P040	0.98
RF00017-458	RNA Gene	4	GC01M20	0.98
RF00017-4588	RNA Gene	4	GC05M038	0.98
RF00017-4629	RNA Gene	4	GC05P056	0.98
RF00017-4970	RNA Gene	4	GC05P142	0.98
piR-32285-085	RNA Gene	4	GC07P107	0.98
RF00017-4711	RNA Gene	4	GC05P072	0.98
ENSG00000285552	RNA Gene	4	GC05P040	0.98
ENSG00000285560	RNA Gene	4	GC15M09(	0.98
lnc-CCDC8-6	RNA Gene	4	GC19M04	0.98
lnc-SFMBT1-5	RNA Gene	4	GC03M052	0.98
lnc-CAB39L-9	RNA Gene	4	GC13M04	0.98
lnc-CDH3-5	RNA Gene	4	GC16P068	0.98
piR-49732-033	RNA Gene	4	GC10M00	0.98
lnc-SLC37A1-2	RNA Gene	4	GC21P042	0.98
piR-41306-110	RNA Gene	4	GC17M02	0.98

lnc-CEP76-2	RNA Gene	4	GC18M01(	0.98
piR-33432-055	RNA Gene	4	GC10M062	0.98
RF00026-1103	RNA Gene	4	GC08P137	0.98
ENSG00000286629	RNA Gene	4	GC17P064	0.98
RF00017-5677	RNA Gene	4	GC06M09(	0.98
piR-48759-204	RNA Gene	4	GC03P053	0.98
piR-56902	RNA Gene	4	GC17P027	0.98
piR-48852	RNA Gene	4	GC03P049	0.98
lnc-BOLL-6	RNA Gene	4	GC02M19	0.98
ENSG00000288064	RNA Gene	4	GC02P191	0.98
piR-57133-396	RNA Gene	4	GC22M04	0.98
ENSG00000287967	RNA Gene	4	GC22M02	0.98
lnc-ADCY7-3	RNA Gene	4	GC16P050	0.98
RF00017-3409	RNA Gene	4	GC02P203	0.98
lnc-RIPK2-3	RNA Gene	4	GC08P089	0.98
RF00017-356	RNA Gene	4	GC01P154	0.98
RF00017-3596	RNA Gene	4	GC20P044	0.98
ENSG00000280010	Uncategor	4	GC11P058	0.98
RF00017-3761	RNA Gene	4	GC22P024	0.98
RF00017-3762	RNA Gene	4	GC22P024	0.98
piR-48259	RNA Gene	4	GC18P079	0.98
ENSG00000283504	RNA Gene	4	GC07M14	0.98
lnc-ANKRD33B-5	RNA Gene	4	GC05P010	0.98
lnc-ANKRD33B-6	RNA Gene	4	GC05P010	0.98
lnc-RPL21-6	RNA Gene	4	GC13P026	0.98
piR-31937-161	RNA Gene	4	GC04M10	0.98
RF00017-3132	RNA Gene	4	GC02M02	0.98
5EW4_A-043	RNA Gene	4	GC05M17	0.98
RF00017-3201	RNA Gene	4	GC02M06(	0.98
piR-48820-008	RNA Gene	4	GC01M15	0.98
RF00017-7988	RNA Gene	4	GC09M12	0.98
piR-41306-095	RNA Gene	4	GC16P011	0.98
lnc-STMN3-5	RNA Gene	4	GC20M06	0.98
piR-55655-375	RNA Gene	4	GC22M04	0.98
piR-47211-583	RNA Gene	4	GC07M00	0.98
piR-39098-183	RNA Gene	4	GC04M12	0.98
piR-55948-040	RNA Gene	4	GC17P034	0.98
piR-39099-151	RNA Gene	4	GC03P013	0.98
piR-55783	RNA Gene	4	GC16P028	0.98
RF00017-1397	RNA Gene	4	GC13M04(	0.98
piR-48007	RNA Gene	4	GC12M11	0.98
piR-55829-003	RNA Gene	4	GC14M03	0.98
piR-31292-113	RNA Gene	4	GC21P015	0.98
RF00017-092	RNA Gene	4	GC01P019	0.98
RF00017-299	RNA Gene	4	GC01M11	0.98
lnc-PDGFB-3	RNA Gene	4	GC22M04	0.98
ENSG00000235679	Pseudoger	4	GC10P030	0.98
LOC10537 Uncharact	RNA Gene	4	GC16M01(	0.98
ZYXP1 Zyxin Pseu	Pseudoger	4	GC08M13	0.98
ENSG00000262488	Pseudoger	4	GC16M01(	0.98
ENSG00000272779	Pseudoger	4	GC22P022	0.98
ENSG00000272980	RNA Gene	4	GC06P167	0.98
ENSG00000273112	RNA Gene	4	GC01P161	0.98
piR-31937-039	RNA Gene	4	GC11P128	0.98
ENSG00000275103	Pseudoger	4	GC08P089	0.98
ENSG00000270204	Pseudoger	4	GC11P114	0.98
HSALNG0133311	RNA Gene	4	GC21M04	0.98
piR-58538-006	RNA Gene	3	GC16P085	0.98

piR-58538-007		RNA Gene	3	GC16P085	0.98
piR-58538-008		RNA Gene	3	GC16P085	0.98
piR-58538-009		RNA Gene	3	GC16P085	0.98
piR-58538-010		RNA Gene	3	GC16P085	0.98
piR-58538-011		RNA Gene	3	GC16P085	0.98
piR-58538-012		RNA Gene	3	GC16P085	0.98
piR-58538-013		RNA Gene	3	GC16P085	0.98
piR-58538-014		RNA Gene	3	GC16P085	0.98
piR-58538-015		RNA Gene	3	GC16P085	0.98
piR-60146-087		RNA Gene	3	GC02P197	0.98
LOC10192	Uncharact	RNA Gene	3	GC18M079	0.98
piR-38580-144		RNA Gene	3	GC19P034	0.98
LOC10537	Uncharact	RNA Gene	3	GC21M044	0.98
LOC10537	Uncharact	RNA Gene	3	GC01M206	0.98
LOC10537	Uncharact	RNA Gene	3	GC02M198	0.98
LOC10536	Uncharact	RNA Gene	3	GC12P068	0.98
ENSG00000253654		Pseudoger	3	GC08M048	0.98
ENSG00000250839		Pseudoger	3	GC04P179	0.98
LOC64634	Spermine	Pseudoger	3	GC01P160	0.98
piR-43105-054		RNA Gene	3	GC10M035	0.98
HSALNG0032571		RNA Gene	3	GC04P004	0.98
RF00994-819		RNA Gene	3	GC05M040	0.98
HSALNG0001583		RNA Gene	3	GC01P022	0.98
LOC10272	Uncharact	RNA Gene	3	GC05P096	0.98
ENSG00000230785		Pseudoger	3	GC07M117	0.98
LOC10050	39S Ribos	Pseudoger	3	GC12P068	0.98
ENSG00000213080		Pseudoger	3	GC01P160	0.98
piR-36393-409		RNA Gene	3	GC05P038	0.98
HSALNG0041356		RNA Gene	3	GC05M040	0.98
lnc-ICAM3-2		RNA Gene	3	GC19M010	0.98
LOC10042	Galactose-	Pseudoger	3	GC02P240	0.98
ENSG00000229172		Pseudoger	3	GC02P227	0.98
piR-56451-093		RNA Gene	3	GC07M107	0.98
piR-48749-023		RNA Gene	3	GC02M198	0.98
piR-56759-041		RNA Gene	3	GC10M034	0.98
piR-40110-155		RNA Gene	3	GC13M040	0.98
ENSG00000285810		RNA Gene	3	GC10P075	0.98
piR-33212		RNA Gene	3	GC10P102	0.98
piR-40398		RNA Gene	3	GC18M017	0.98
ENSG00000279625		Uncategor	3	GC01M022	0.98
ENSG00000286565		RNA Gene	3	GC07P152	0.98
piR-39767-002		RNA Gene	3	GC01M002	0.98
piR-55655-210		RNA Gene	3	GC16P030	0.98
RF00017-239		RNA Gene	3	GC01M067	0.98
RF00017-2046		RNA Gene	3	GC16P030	0.98
ENSG00000270335		Pseudoger	3	GC02M062	0.98
ENSG00000253843		Pseudoger	2	GC08M048	0.98
ENSG00000244245		Pseudoger	2	GC05P108	0.98
LOC10537	Uncharact	RNA Gene	2	GC07M107	0.98
lnc-TNFSF15-5		RNA Gene	2	GC09M114	0.98
ENSG00000233020		Pseudoger	2	GC01P070	0.98
ENSG00000224104		Pseudoger	2	GC07M153	0.98
piR-32810-138		RNA Gene	2	GC09M114	0.98
ENSG00000287290		RNA Gene	2	GC04M116	0.98
ENSG00000286119		RNA Gene	2	GC04P182	0.98
ENSG00000276949		Pseudoger	2	GC02P240	0.98
ENSG00000277191		Pseudoger	2	GC16P028	0.98
GKN1	Gastrokine	Protein Co	39	GC02P068	0.96

VASP	Vasodilato	Protein Co	44	GC19P045	0.95
SOCS2	Suppresso	Protein Co	45	GC12P093	0.94
TFPI2	Tissue Fac	Protein Co	43	GC07M093	0.94
FMR1	FMRP Trar	Protein Co	46	GC0XP147	0.93
CLCN5	Chloride V	Protein Co	43	GC0XP049	0.93
VCAN	Versican	Protein Co	48	GC05P083	0.93
INPP5D	Inositol Po	Protein Co	47	GC02P233	0.92
TAB1	TGF-Beta ,	Protein Co	44	GC22P039	0.92
FADS1	Fatty Acid	Protein Co	44	GC11M061	0.92
PNPLA3	Patatin Lik	Protein Co	42	GC22P043	0.92
HIC1	HIC ZBTB	Protein Co	41	GC17P002	0.92
IPMK	Inositol Po	Protein Co	40	GC10M058	0.92
MGAT3	Beta-1,4-N	Protein Co	40	GC22P039	0.92
PRICKLE2	Prickle Pla	Protein Co	39	GC03M064	0.92
ASAH1	N-Acylsph	Protein Co	47	GC08M018	0.92
CALB2	Calbindin	Protein Co	42	GC16P071	0.92
SATB1	SATB Hom	Protein Co	43	GC03M018	0.92
EPAS1	Endothelia	Protein Co	50	GC02P046	0.92
ALDH9A1	Aldehyde	Protein Co	42	GC01M165	0.9
GALK1	Galactokin	Protein Co	49	GC17M075	0.89
HMGA2	High Mobi	Protein Co	46	GC12P065	0.89
TNFSF13	TNF Super	Protein Co	45	GC17P007	0.89
REG1B	Regenerat	Protein Co	39	GC02M079	0.89
SOAT1	Sterol O-A	Protein Co	45	GC01P179	0.89
ALPK1	Alpha Kina	Protein Co	38	GC04P112	0.89
ABCB7	ATP Bindir	Protein Co	44	GC0XM075	0.88
CD99	CD99 Mol	Protein Co	41	GC0XP002	0.88
SELENOS	Selenopro	Protein Co	32	GC15M102	0.87
ZNF281	Zinc Finge	Protein Co	38	GC01M200	0.85
HCK	HCK Proto	Protein Co	51	GC20P032	0.85
CACNA1C	Calcium V	Protein Co	50	GC12P001	0.85
PGM1	Phosphog	Protein Co	49	GC01P063	0.85
RPS6KA2	Ribosomal	Protein Co	48	GC06M166	0.85
VRK1	VRK Serine	Protein Co	48	GC14P096	0.85
EIF2AK2	Eukaryotic	Protein Co	47	GC02M037	0.85
POU2F1	POU Class	Protein Co	46	GC01P167	0.85
CSF2RA	Colony Sti	Protein Co	46	GC0XP001	0.85
ATP2B2	ATPase Pla	Protein Co	46	GC03M010	0.85
IRS2	Insulin Rec	Protein Co	45	GC13M109	0.85
SPRY4	Sprouty R	Protein Co	45	GC05M142	0.85
CTSZ	Cathepsin	Protein Co	45	GC20M058	0.85
SPHK2	Sphingosir	Protein Co	44	GC19P048	0.85
PLTP	Phospholi	Protein Co	44	GC20M045	0.85
RASGRF1	Ras Protei	Protein Co	44	GC15M078	0.85
CLN3	CLN3 Lyso	Protein Co	44	GC16M028	0.85
RNASET2	Ribonucle	Protein Co	44	GC06M166	0.85
LAMA5	Laminin Su	Protein Co	44	GC20M062	0.85
NLRP2	NLR Famil	Protein Co	43	GC19P054	0.85
IL31RA	Interleukin	Protein Co	43	GC05P055	0.85
SPRED1	Sprouty Re	Protein Co	43	GC15P038	0.85
PADI2	Peptidyl A	Protein Co	43	GC01M017	0.85
CDC37	Cell Divisic	Protein Co	43	GC19M010	0.85
SULF2	Sulfatase 2	Protein Co	41	GC20M047	0.85
ST8SIA4	ST8 Alpha	Protein Co	41	GC05M100	0.85
CREB5	CAMP Res	Protein Co	41	GC07P028	0.85
UBQLN4	Ubiquilin 4	Protein Co	41	GC01M156	0.85
NOX3	NADPH O	Protein Co	40	GC06M155	0.85
SPRED2	Sprouty Re	Protein Co	40	GC02M065	0.85

RAB13	RAB13, Me	Protein Co	40	GC01M153	0.85
FLRT1	Fibronecti	Protein Co	40	GC11P064	0.85
EPS8L2	EPS8 Like 2	Protein Co	40	GC11P000	0.85
POP4	POP4 Hom	Protein Co	39	GC19P029	0.85
RBFOX1	RNA Bindi	Protein Co	39	GC16P005	0.85
DBP	D-Box Bin	Protein Co	39	GC19M048	0.85
CEBPG	CCAAT En	Protein Co	39	GC19P033	0.85
ZPBP	Zona Pellu	Protein Co	39	GC07M049	0.85
SYT4	Synaptotag	Protein Co	38	GC18M043	0.85
STXBP4	Syntaxin B	Protein Co	38	GC17P054	0.85
MMP28	Matrix Me	Protein Co	38	GC17M035	0.85
RSPO3	R-Spondir	Protein Co	38	GC06P127	0.85
TMTC2	Transmem	Protein Co	37	GC12P082	0.85
MPPED2	Metalloph	Protein Co	37	GC11M030	0.85
CSMD2	CUB And S	Protein Co	37	GC01M033	0.85
TM9SF4	Transmem	Protein Co	37	GC20P032	0.85
TUBD1	Tubulin De	Protein Co	37	GC17M059	0.85
PF4V1	Platelet Fa	Protein Co	37	GC04P073	0.85
SLC10A4	Solute Car	Protein Co	36	GC04P048	0.85
SNX7	Sorting Ne	Protein Co	36	GC01P098	0.85
RABEP2	Rabaptin, I	Protein Co	36	GC16M028	0.85
GALNTL6	Polypeptic	Protein Co	36	GC04P171	0.85
IZUMO1	Izumo Spe	Protein Co	35	GC19M048	0.85
SLAIN2	SLAIN Mot	Protein Co	35	GC04P048	0.85
OR2AT4	Olfactory I	Protein Co	35	GC11M075	0.85
KIAA1841	KIAA1841	Protein Co	35	GC02P061	0.85
TRPT1	TRNA Pho	Protein Co	35	GC11M064	0.85
PHACTR2	Phosphata	Protein Co	35	GC06P143	0.85
ZNF532	Zinc Finge	Protein Co	35	GC18P058	0.85
PRRC1	Proline Ric	Protein Co	33	GC05P127	0.85
MORC4	MORC Far	Protein Co	33	GC0XM10(	0.85
BABAM2	BRISC And	Protein Co	32	GC02P027	0.85
LRRC61	Leucine Ri	Protein Co	32	GC07P150	0.85
TM6SF2	Transmem	Protein Co	30	GC19M019	0.85
C1orf53	Chromoso	Protein Co	29	GC01P197	0.85
RIMBP3	RIMS Bind	Protein Co	28	GC22M018	0.85
C2orf74	Chromoso	Protein Co	28	GC02P061	0.85
BORCS5	BLOC-1 Re	Protein Co	27	GC12P012	0.85
PPAN-P2R	PPAN-P2R	Protein Co	25	GC19P010	0.85
ZNF300P1	Zinc Finge	Pseudoger	16	GC05M150	0.85
MIR6727	MicroRNA	RNA Gene	9	GC01M001	0.85
OR2AT1P	Olfactory I	Pseudoger	9	GC11M075	0.85
OR7E116P	Olfactory I	Pseudoger	8	GC09P090	0.85
RPL21P108	Ribosomal	Pseudoger	6	GC13M074	0.85
RPL18P7	Ribosomal	Pseudoger	6	GC08P113	0.85
RPL35AP7	Ribosomal	Pseudoger	6	GC01M164	0.85
RNU7-67P	RNA, U7 S	Pseudoger	6	GC08P101	0.85
LAMP3	Lysosomal	Protein Co	40	GC03M183	0.84
NTRK3	Neurotrop	Protein Co	52	GC15M087	0.83
BMPR2	Bone Morp	Protein Co	51	GC02P202	0.83
CCNE1	Cyclin E1	Protein Co	48	GC19P029	0.83
ACO1	Aconitase	Protein Co	45	GC09P032	0.83
LTC4S	Leukotrien	Protein Co	43	GC05P179	0.83
TRIM5	Tripartite M	Protein Co	43	GC11M005	0.83
SLCO4A1	Solute Car	Protein Co	43	GC20P062	0.83
PDLIM1	PDZ And L	Protein Co	42	GC10M095	0.83
TRAIP	TRAF Inter	Protein Co	41	GC03M049	0.83
UBASH3B	Ubiquitin A	Protein Co	41	GC11P122	0.83

ABCF2	ATP Bindir	Protein Co	40	GC07M151	0.83
NCR1	Natural Cy	Protein Co	39	GC19P055	0.83
IL31	Interleukin	Protein Co	36	GC12M122	0.83
C13orf42	Chromoso	Protein Co	11	GC13M051	0.83
TRIM33	Tripartite M	Protein Co	45	GC01M114	0.82
IQGAP1	IQ Motif C	Protein Co	45	GC15P090	0.8
SSTR2	Somatosta	Protein Co	49	GC17P073	0.8
AMPD2	Adenosine	Protein Co	49	GC01P109	0.8
AMPD3	Adenosine	Protein Co	47	GC11P010	0.8
TAOK1	TAO Kinas	Protein Co	44	GC17P029	0.8
CASP6	Caspase 6	Protein Co	50	GC04M109	0.8
CDH11	Cadherin 1	Protein Co	48	GC16M064	0.8
GRN	Granulin P	Protein Co	48	GC17P044	0.8
SLC7A5	Solute Car	Protein Co	45	GC16M087	0.8
USP8	Ubiquitin S	Protein Co	49	GC15P050	0.78
SPINT2	Serine Pep	Protein Co	45	GC19P038	0.78
RNF31	Ring Finge	Protein Co	43	GC14P024	0.78
SHARPIN	SHANK As	Protein Co	41	GC08M144	0.78
IL17RE	Interleukin	Protein Co	41	GC03P009	0.78
CD101	CD101 Mo	Protein Co	40	GC01P117	0.75
MYLK	Myosin Lig	Protein Co	54	GC03M123	0.75
PRLR	Prolactin R	Protein Co	50	GC05M035	0.75
PGK1	Phosphog	Protein Co	50	GC0XP077	0.75
CPT1A	Carnitine P	Protein Co	49	GC11M068	0.75
NR1H3	Nuclear Re	Protein Co	48	GC11P047	0.75
CES1	Carboxyles	Protein Co	48	GC16M055	0.75
AKR1C2	Aldo-Keto	Protein Co	48	GC10M004	0.75
ADAM9	ADAM Me	Protein Co	48	GC08P038	0.75
ARHGDIA	Rho GDP D	Protein Co	48	GC17M081	0.75
GRM8	Glutamate	Protein Co	47	GC07M126	0.75
MTNR1B	Melatonin	Protein Co	47	GC11P092	0.75
CBR1	Carbonyl R	Protein Co	47	GC21P036	0.75
FPR2	Formyl Pep	Protein Co	47	GC19P051	0.75
ANGPTL4	Angiopoie	Protein Co	47	GC19P008	0.75
ACADVL	Acyl-CoA	Protein Co	47	GC17P007	0.75
ITGB6	Integrin Su	Protein Co	47	GC02M160	0.75
MGLL	Monoglyce	Protein Co	46	GC03M127	0.75
TLE1	TLE Family	Protein Co	45	GC09M081	0.75
CYP26B1	Cytochrom	Protein Co	45	GC02M072	0.75
HES1	Hes Family	Protein Co	45	GC03P194	0.75
ANXA4	Annexin A	Protein Co	45	GC02P069	0.75
PON2	Paraoxona	Protein Co	44	GC07M095	0.75
PANX1	Pannexin 1	Protein Co	44	GC11P094	0.75
MTNR1A	Melatonin	Protein Co	44	GC04M186	0.75
GGH	Gamma-G	Protein Co	44	GC08M063	0.75
GALNT2	Polypeptic	Protein Co	44	GC01P230	0.75
AKR1C1	Aldo-Keto	Protein Co	44	GC10P004	0.75
TPP1	Tripeptidyl	Protein Co	44	GC11M006	0.75
JUNB	JunB Proto	Protein Co	43	GC19P012	0.75
GSTA1	Glutathion	Protein Co	43	GC06M052	0.75
HNF4G	Hepatocyt	Protein Co	43	GC08P075	0.75
ADAM19	ADAM Me	Protein Co	43	GC05M157	0.75
SULT1B1	Sulfotransf	Protein Co	43	GC04M069	0.75
HSPB2	Heat Shoc	Protein Co	42	GC11P111	0.75
GPC5	Glypican 5	Protein Co	42	GC13P091	0.75
TAGLN2	Transgelin	Protein Co	41	GC01M159	0.75
GSTK1	Glutathion	Protein Co	41	GC07P144	0.75
GSTA2	Glutathion	Protein Co	41	GC06M052	0.75

CLDN11	Claudin 11	Protein Co	41	GC03P170	0.75
NCAPD3	Non-SMC	Protein Co	41	GC11M134	0.75
ALDOC	Aldolase, F	Protein Co	41	GC17M029	0.75
FLOT2	Flotillin 2	Protein Co	41	GC17M029	0.75
FN3K	Fructosam	Protein Co	41	GC17P082	0.75
USP25	Ubiquitin S	Protein Co	41	GC21P015	0.75
PPP2R5E	Protein Ph	Protein Co	40	GC14M063	0.75
PPP3R2	Protein Ph	Protein Co	40	GC09M101	0.75
PSG1	Pregnancy	Protein Co	40	GC19M042	0.75
HNRNPH3	Heteroger	Protein Co	40	GC10P068	0.75
RNF20	Ring Finge	Protein Co	40	GC09P101	0.75
FIS1	Fission, Mi	Protein Co	40	GC07M101	0.75
CALCOCO	Calcium Bi	Protein Co	40	GC17P048	0.75
USO1	USO1 Vesi	Protein Co	40	GC04P075	0.75
NOXO1	NADPH O:	Protein Co	39	GC16M002	0.75
IBSP	Integrin Bi	Protein Co	39	GC04P087	0.75
PANX2	Pannexin 2	Protein Co	39	GC22P050	0.75
LSAMP	Limbic Sys	Protein Co	39	GC03M115	0.75
CLEC4A	C-Type Le	Protein Co	38	GC12P008	0.75
EXOSC1	Exosome C	Protein Co	38	GC10M097	0.75
PIH1D1	PIH1 Dom	Protein Co	37	GC19M049	0.75
PTER	Phosphotr	Protein Co	37	GC10P016	0.75
CLEC2D	C-Type Le	Protein Co	37	GC12P010	0.75
CNN3	Calponin 3	Protein Co	37	GC01M094	0.75
BEST2	Bestrophir	Protein Co	36	GC19P012	0.75
MAGI3	Membrane	Protein Co	36	GC01P113	0.75
DNAH12	Dynein Ax	Protein Co	35	GC03M057	0.75
DNPH1	2'-Deoxyn	Protein Co	35	GC06M043	0.75
ZNF649	Zinc Finge	Protein Co	35	GC19M051	0.75
ACTL8	Actin Like	Protein Co	34	GC01P017	0.75
BEST4	Bestrophir	Protein Co	33	GC01M044	0.75
ZG16	Zymogen	Protein Co	33	GC16P029	0.75
PROSER1	Proline An	Protein Co	30	GC13M039	0.75
C10orf67	Chromoso	Protein Co	30	GC10M023	0.75
H2BC3	H2B Cluste	Protein Co	29	GC06M026	0.75
MIR595	MicroRNA	RNA Gene	16	GC07M158	0.75
MIR1246	MicroRNA	RNA Gene	13	GC02M176	0.75
LOC40086	Uncharact	RNA Gene	11	GC21M038	0.75
NM	Neutrophi	Genetic Lo	8	GC07U990	0.75
IL6STP1	Interleukin	Pseudoger	8	GC17P015	0.75
OMP	Olfactory I	Protein Co	39	GC11P077	0.75
ABCC4	ATP Bindir	Protein Co	47	GC13M095	0.74
MIR425	MicroRNA	RNA Gene	18	GC03M049	0.74
MYH9	Myosin He	Protein Co	50	GC22M036	0.72
ACTB	Actin Beta	Protein Co	50	GC07M005	0.72
TTF2	Transcripti	Protein Co	40	GC01P117	0.7
STK38	Serine/Thr	Protein Co	44	GC06M036	0.69
CHKB	Choline Ki	Protein Co	47	GC22M050	0.68
SREBF2	Sterol Reg	Protein Co	44	GC22P041	0.68
TRAF4	TNF Recep	Protein Co	44	GC17P028	0.68
CD47	CD47 Mol	Protein Co	45	GC03M108	0.68
NAA15	N-Alpha-A	Protein Co	40	GC04P139	0.68
PIK3CD	Phosphati	Protein Co	54	GC01P009	0.65
LIMK2	LIM Doma	Protein Co	49	GC22P031	0.65
KMT2A	Lysine Met	Protein Co	45	GC11P118	0.65
ACTR2	Actin Relat	Protein Co	44	GC02P065	0.65
CHD3	Chromodc	Protein Co	43	GC17P007	0.65
CD177	CD177 Mc	Protein Co	40	GC19P043	0.65

YBX2	Y-Box Binc	Protein Co	39	GC17M007	0.65
TARS1	Threonyl-T	Protein Co	36	GC05P033	0.65
GRAP2	GRB2 Rela	Protein Co	44	GC22P039	0.63
P2RY6	Pyrimidine	Protein Co	43	GC11P073	0.59
PPM1D	Protein Ph	Protein Co	49	GC17P060	0.58
TSPO	Translocat	Protein Co	44	GC22P043	0.58
TRAF5	TNF Recep	Protein Co	44	GC01P211	0.56
NDRG1	N-Myc Dc	Protein Co	47	GC08M133	0.54
PROK2	Prokinetici	Protein Co	44	GC03M071	0.54
LPO	Lactopero:	Protein Co	40	GC17P058	0.54
AQP3	Aquaporin	Protein Co	48	GC09M033	0.54
MAP3K1	Mitogen-A	Protein Co	51	GC05P056	0.51
PON3	Paraoxona	Protein Co	45	GC07M095	0.51
KLRB1	Killer Cell L	Protein Co	43	GC12M013	0.51
ICA1	Islet Cell A	Protein Co	41	GC07M008	0.51
OLFM4	Olfactome	Protein Co	39	GC13P053	0.51
OTUD1	OTU Deub	Protein Co	33	GC10P023	0.51
PNOC	Prepronoc	Protein Co	38	GC08P028	0.49
KAT2B	Lysine Ace	Protein Co	50	GC03P020	0.49
LTB4R	Leukotrien	Protein Co	45	GC14P024	0.49
MIP	Major Intri	Protein Co	43	GC12M056	0.49
AQP10	Aquaporin	Protein Co	38	GC01P154	0.49
ARNT	Aryl Hydrc	Protein Co	45	GC01M150	0.47
NTF4	Neurotrop	Protein Co	45	GC19M049	0.41
DVL2	Dishevelle	Protein Co	47	GC17M007	0.37
CCL28	C-C Motif	Protein Co	41	GC05M043	0.37
ITGAV	Integrin Su	Protein Co	48	GC02P186	0.34
TPM3	Tropomyo	Protein Co	47	GC01M154	0.34
MPST	Mercaptop	Protein Co	44	GC22P037	0.34
FUT8	Fucosyltra	Protein Co	44	GC14P065	0.34
MNAT1	MNAT1 Cc	Protein Co	43	GC14P060	0.34
CCDC22	Coiled-Co	Protein Co	39	GC0XP049	0.34
HDAC4	Histone De	Protein Co	52	GC02M239	0.33
GFRA1	GDNF Fam	Protein Co	45	GC10M116	0.33
GUCY2D	Guanylate	Protein Co	44	GC17P008	0.33
MDM4	MDM4 Reg	Protein Co	44	GC01P204	0.33
MIR22HG	MIR22 Hos	RNA Gene	27	GC17M001	0.33
PI4KB	Phosphatic	Protein Co	45	GC01M151	0.3
EGLN3	Egl-9 Fam	Protein Co	48	GC14M033	0.29
EIF2B4	Eukaryotic	Protein Co	45	GC02M027	0.29
CLSPN	Claspin	Protein Co	41	GC01M035	0.29
ATF1	Activating	Protein Co	48	GC12P050	0.29
MCM3	Minichrom	Protein Co	47	GC06M052	0.29
FSTL1	Follistatin	Protein Co	43	GC03M120	0.29
FUT7	Fucosyltra	Protein Co	42	GC09M137	0.29
UBD	Ubiquitin D	Protein Co	40	GC06M029	0.29
DYRK1A	Dual Speci	Protein Co	52	GC21P037	0.24
AXL	AXL Recep	Protein Co	52	GC19P041	0.24
PLD1	Phospholip	Protein Co	50	GC03M171	0.24
RYR2	Ryanodine	Protein Co	48	GC01P237	0.24
WEE1	WEE1 G2 C	Protein Co	48	GC11P009	0.24
P2RY2	Purinergic	Protein Co	47	GC11P073	0.24
GRM3	Glutamate	Protein Co	47	GC07P086	0.24
CYP11B1	Cytochron	Protein Co	47	GC08M142	0.24
CHRNA7	Cholinergi	Protein Co	47	GC15P031	0.24
TNFRSF11A	TNF Recep	Protein Co	47	GC18P062	0.24
ITGB5	Integrin Su	Protein Co	47	GC03M124	0.24
SLC29A2	Solute Car	Protein Co	46	GC11M066	0.24

DLG1	Discs Larg	Protein Co	46	GC03M19	0.24
RPSA	Ribosomal	Protein Co	46	GC03P039	0.24
B4GALT1	Beta-1,4-(	Protein Co	46	GC09M03	0.24
CNTN1	Contactin	Protein Co	45	GC12P040	0.24
PTGFR	Prostaglan	Protein Co	45	GC01P078	0.24
KL	Klotho	Protein Co	45	GC13P033	0.24
MAP3K14	Mitogen-A	Protein Co	45	GC17M04	0.24
CTRC	Chymotryp	Protein Co	45	GC01P015	0.24
CHD2	Chromodo	Protein Co	45	GC15P092	0.24
DOCK2	Dedicator	Protein Co	45	GC05P169	0.24
AKR1B10	Aldo-Keto	Protein Co	45	GC07P134	0.24
CACNA1I	Calcium V	Protein Co	45	GC22P039	0.24
FABP1	Fatty Acid	Protein Co	45	GC02M08	0.24
VAV2	Vav Guani	Protein Co	45	GC09M13	0.24
VPS35	VPS35 Ret	Protein Co	45	GC16M04	0.24
UBE2D3	Ubiquitin (	Protein Co	45	GC04M10	0.24
INHBA	Inhibin Sul	Protein Co	45	GC07M04	0.24
SLCO2B1	Solute Car	Protein Co	45	GC11P075	0.24
ENPEP	Glutamyl A	Protein Co	44	GC04P110	0.24
KCNN3	Potassium	Protein Co	44	GC01M15	0.24
CYFIP2	Cytoplasm	Protein Co	44	GC05P157	0.24
COPS5	COP9 Sign	Protein Co	44	GC08M06	0.24
VAPA	VAMP Ass	Protein Co	44	GC18P009	0.24
UBB	Ubiquitin B	Protein Co	44	GC17P016	0.24
SLC8A3	Solute Car	Protein Co	44	GC14M07	0.24
IGF2BP3	Insulin Like	Protein Co	43	GC07M02	0.24
ITGA11	Integrin Su	Protein Co	43	GC15M06	0.24
SLC28A2	Solute Car	Protein Co	43	GC15P045	0.24
SEPHS1	Selenopho	Protein Co	43	GC10M01	0.24
DPP6	Dipeptidyl	Protein Co	43	GC07P153	0.24
ROBO2	Roundabo	Protein Co	43	GC03P075	0.24
CDH13	Cadherin 1	Protein Co	43	GC16P082	0.24
CACNB1	Calcium V	Protein Co	43	GC17M03	0.24
FABP5	Fatty Acid	Protein Co	43	GC08P081	0.24
TIRAP	TIR Domai	Protein Co	43	GC11P126	0.24
TSFM	Ts Translat	Protein Co	43	GC12P057	0.24
SLIT1	Slit Guidar	Protein Co	43	GC10M09	0.24
NRG3	Neuregulir	Protein Co	42	GC10P083	0.24
SORBS1	Sorbin And	Protein Co	42	GC10M09	0.24
CNN1	Calponin 1	Protein Co	42	GC19P011	0.24
CPD	Carboxype	Protein Co	42	GC17P030	0.24
CADPS	Calcium D	Protein Co	42	GC03M06	0.24
SMYD2	SET And M	Protein Co	42	GC01P214	0.24
NSMAF	Neutral Sp	Protein Co	41	GC08M05	0.24
PTPRR	Protein Ty	Protein Co	41	GC12M07	0.24
CAPZB	Capping A	Protein Co	41	GC01M01	0.24
HNRNPM	Heteroger	Protein Co	41	GC19P008	0.24
ELMO1	Engulfmer	Protein Co	41	GC07M03	0.24
SBF1	SET Bindin	Protein Co	41	GC22M05	0.24
GPM6A	Glycoprote	Protein Co	41	GC04M17	0.24
CD200R1	CD200 Rec	Protein Co	41	GC03M11	0.24
MEGF10	Multiple E	Protein Co	41	GC05P127	0.24
ABCB9	ATP Bindir	Protein Co	41	GC12M12	0.24
LRRC4C	Leucine Ri	Protein Co	41	GC11M04	0.24
CAPZA1	Capping A	Protein Co	41	GC01P112	0.24
ARID5B	AT-Rich In	Protein Co	41	GC10P061	0.24
CALU	Calumenir	Protein Co	41	GC07P128	0.24
TNFRSF19	TNF Recep	Protein Co	41	GC13P023	0.24

VPS26A	VPS26, Ret	Protein Co	41	GC10P069	0.24
SMARCD3	SWI/SNF F	Protein Co	41	GC07M151	0.24
PDSS2	Decapreny	Protein Co	41	GC06M107	0.24
PIK3AP1	Phosphoin	Protein Co	40	GC10M096	0.24
PARVB	Parvin Beta	Protein Co	40	GC22P043	0.24
HCST	Hematopo	Protein Co	40	GC19P037	0.24
GPR158	G Protein-	Protein Co	40	GC10P025	0.24
DNER	Delta/Notc	Protein Co	40	GC02M229	0.24
CDK18	Cyclin Dep	Protein Co	40	GC01P205	0.24
ALDH1L2	Aldehyde	Protein Co	40	GC12M105	0.24
LRP1B	LDL Recep	Protein Co	40	GC02M140	0.24
ARHGAP24	Rho GTPas	Protein Co	40	GC04P085	0.24
EPB41L2	Erythrocyt	Protein Co	40	GC06M130	0.24
AUTS2	Activator (	Protein Co	40	GC07P069	0.24
TIGAR	TP53 Indu	Protein Co	39	GC12P006	0.24
SNRPC	Small Nucl	Protein Co	39	GC06P046	0.24
PLCH1	Phospholip	Protein Co	39	GC03M155	0.24
SOX13	SRY-Box T	Protein Co	39	GC01P204	0.24
TAF3	TATA-Box	Protein Co	39	GC10P007	0.24
STAB2	Stabilin 2	Protein Co	39	GC12P103	0.24
ELL2	Elongation	Protein Co	39	GC05M095	0.24
NCR2	Natural Cy	Protein Co	39	GC06P046	0.24
CRISP3	Cysteine R	Protein Co	39	GC06M049	0.24
COL19A1	Collagen T	Protein Co	39	GC06P069	0.24
MRPL28	Mitochond	Protein Co	39	GC16M000	0.24
MTSS1	MTSS I-BA	Protein Co	39	GC08M124	0.24
MEF2B	Myocyte E	Protein Co	39	GC19M019	0.24
ABI3	ABI Family	Protein Co	39	GC17P049	0.24
BRWD1	Bromodom	Protein Co	39	GC21M039	0.24
LRRTM4	Leucine Ri	Protein Co	39	GC02M076	0.24
FMNL2	Formin Lik	Protein Co	39	GC02P152	0.24
LY75	Lymphocy	Protein Co	39	GC02M159	0.24
USH2A	Usherin	Protein Co	39	GC01M215	0.24
SMYD1	SET And M	Protein Co	39	GC02P088	0.24
KCNIP4	Potassium	Protein Co	39	GC04M020	0.24
POU6F2	POU Class	Protein Co	38	GC07P038	0.24
RALGAPA2	Ral GTPase	Protein Co	38	GC20M020	0.24
CCDC40	Coiled-Co	Protein Co	38	GC17P080	0.24
ANO2	Anoctamin	Protein Co	38	GC12M005	0.24
CDH22	Cadherin 2	Protein Co	38	GC20M046	0.24
HUNK	Hormonall	Protein Co	37	GC21P031	0.24
JAKMIP1	Janus Kina	Protein Co	37	GC04M006	0.24
SYTL1	Synaptota	Protein Co	37	GC01P027	0.24
TMEM132I	Transmem	Protein Co	37	GC12M129	0.24
CSMD1	CUB And S	Protein Co	37	GC08M002	0.24
MPRIP	Myosin Ph	Protein Co	37	GC17P017	0.24
HOXD3	Homeobo	Protein Co	37	GC02P176	0.24
SCUBE1	Signal Pep	Protein Co	37	GC22M043	0.24
CCNG2	Cyclin G2	Protein Co	37	GC04P077	0.24
MDGA2	MAM Dom	Protein Co	37	GC14M046	0.24
TRIM6	Tripartite M	Protein Co	37	GC11P005	0.24
GDE1	Glyceroph	Protein Co	37	GC16M019	0.24
RAB3C	RAB3C, Me	Protein Co	37	GC05P058	0.24
TBC1D14	TBC1 Dom	Protein Co	36	GC04P006	0.24
SYT9	Synaptota	Protein Co	36	GC11P007	0.24
MON2	MON2 Ho	Protein Co	36	GC12P062	0.24
CPNE8	Copine 8	Protein Co	36	GC12M038	0.24
DERL3	Derlin 3	Protein Co	36	GC22M023	0.24

DNAH10	Dynein Ax	Protein Co	36	GC12P123	0.24
RTCB	RNA 2',3'-	Protein Co	36	GC22M032	0.24
SGCZ	Sarcoglyca	Protein Co	36	GC08M014	0.24
DNASE2B	Deoxyribo	Protein Co	36	GC01P084	0.24
EYS	Eyes Shut	Protein Co	36	GC06M063	0.24
FCGBP	Fc Fragme	Protein Co	36	GC19M039	0.24
AVIL	Advillin	Protein Co	36	GC12M057	0.24
UBE2U	Ubiquitin (	Protein Co	36	GC01P064	0.24
VPS13C	Vacuolar F	Protein Co	36	GC15M061	0.24
SLC38A10	Solute Car	Protein Co	36	GC17M081	0.24
RBM26	RNA Bindi	Protein Co	36	GC13M079	0.24
LINGO2	Leucine Ri	Protein Co	36	GC09M027	0.24
CNTNAP5	Contactin	Protein Co	35	GC02P124	0.24
PLD5	Phospholi	Protein Co	35	GC01M242	0.24
CEP131	Centrosom	Protein Co	35	GC17M081	0.24
SAMD14	Sterile Alp	Protein Co	35	GC17M050	0.24
CCDC93	Coiled-Co	Protein Co	35	GC02M117	0.24
FRY	FRY Micro	Protein Co	35	GC13P031	0.24
ADAMDEC	ADAM Like	Protein Co	35	GC08P024	0.24
ARMC3	Armadillo	Protein Co	35	GC10P022	0.24
UMODL1	Uromodul	Protein Co	35	GC21P042	0.24
JPH4	Junctophil	Protein Co	35	GC14M023	0.24
SHISA6	Shisa Fami	Protein Co	34	GC17P011	0.24
ZNF804B	Zinc Finge	Protein Co	34	GC07P088	0.24
EFCAB6	EF-Hand (	Protein Co	33	GC22M044	0.24
SNTG1	Syntrophir	Protein Co	33	GC08P049	0.24
TMEM182	Transmem	Protein Co	33	GC02P102	0.24
KMT5B	Lysine Met	Protein Co	33	GC11M068	0.24
CEP170B	Centrosom	Protein Co	33	GC14P104	0.24
CEACAM4	CEA Cell A	Protein Co	33	GC19M041	0.24
BEND7	BEN Doma	Protein Co	33	GC10M013	0.24
ZNF385D	Zinc Finge	Protein Co	33	GC03M021	0.24
RELL1	RELT Like	Protein Co	32	GC04M037	0.24
TMEM255	Transmem	Protein Co	31	GC13P113	0.24
EMC9	ER Membr	Protein Co	31	GC14M024	0.24
TTLL2	Tubulin Ty	Protein Co	31	GC06P167	0.24
TOPAZ1	Testis And	Protein Co	30	GC03P044	0.24
TENT2	Terminal N	Protein Co	29	GC05P079	0.24
KIAA1257	KIAA1257	Protein Co	29	GC03M129	0.24
WASHC2C	WASH Cor	Protein Co	28	GC10P045	0.24
TRIM6-TR	TRIM6-TR	Protein Co	28	GC11P005	0.24
REC114	REC114 M	Protein Co	27	GC15P073	0.24
NPSR1-AS	NPSR1 An	RNA Gene	20	GC07M034	0.24
LINC00469	Long Inter	RNA Gene	20	GC17M073	0.24
CLRN1-AS	CLRN1 An	RNA Gene	17	GC03P150	0.24
MIR19B2	MicroRNA	RNA Gene	17	GC0XM134	0.24
SNORD48	Small Nucl	RNA Gene	14	GC06P033	0.24
ZNF815P	Zinc Finge	Pseudoger	14	GC07P005	0.24
PRICKLE2-	PRICKLE2	RNA Gene	13	GC03P064	0.24
LINC00331	Long Inter	RNA Gene	13	GC13M078	0.24
LACTB2-A	LACTB2 Ar	RNA Gene	13	GC08P070	0.24
ANKRD34(	ANKRD34(	RNA Gene	12	GC15M079	0.24
LINC01342	Long Inter	RNA Gene	12	GC01P001	0.24
LOC10192	Uncharact	RNA Gene	11	GC05P095	0.24
FILNC1	FOXO Indu	RNA Gene	11	GC06P139	0.24
LINC02367	Long Inter	RNA Gene	11	GC12P010	0.24
IATPR	ITGB1 Adja	RNA Gene	10	GC10M033	0.24
LINC00836	Long Inter	RNA Gene	10	GC10P025	0.24

ISCA2P1	Iron-Sulfu	Pseudoger	9	GC22M026	0.24
LOC10272	Uncharact	RNA Gene	9	GC05M157	0.24
LOC10050	Uncharact	RNA Gene	9	GC11M007	0.24
LRRTM4-A	LRRTM4 A	RNA Gene	9	GC02P076	0.24
PPIAP33	Peptidylpr	Pseudoger	5	GC09P007	0.24
IDDM15	Insulin Dep	Genetic Lc	4	GC06U990	0.24
LOC10192	Uncharact	RNA Gene	3	GC12M055	0.24
