# Supplementary material for: Uncovering the Mechanism of Curcuma in the Treatment of Ulcerative Colitis Based on Network Pharmacology, Molecular Docking Technology, and Experiment Verification
Source: Evid Based Complement Alternat Med. 2021 Jun 16;2021:6629761. doi: 10.1155/2021/6629761 (PMC8225429; doi:10.1155/2021/6629761)
Supplement: Supplementary Materials — Table S1: compound targets for each component in curcuma by prediction. Table S2: UC-related targets in GeneCards and DisGeNet and papers published in CNKI and PubMed. Table S3: component-target-pathway connection. [file 6629761.f1.zip › 6629761.f1/TableS3 COMPONENT-PATHWAT-TARGETS.pdf]

PW	TAR	
PW1	AKT1	Pathways in cancer
PW1	BCL2	Pathways in cancer
PW1	BRAF	Pathways in cancer
PW1	CDK2	Pathways in cancer
PW1	CDK4	Pathways in cancer
PW1	EGFR	Pathways in cancer
PW1	EP300	Pathways in cancer
PW1	MET	Pathways in cancer
PW1	MMP9	Pathways in cancer
PW1	NOS2	Pathways in cancer
PW1	PPARG	Pathways in cancer
PW1	PTGER2	Pathways in cancer
PW1	PTGS2	Pathways in cancer
PW1	RAF1	Pathways in cancer
PW1	STAT3	Pathways in cancer
PW2	RORC	Inflammatory bowel disease (IBD)
PW2	STAT3	Inflammatory bowel disease (IBD)
PW2	TLR4	Inflammatory bowel disease (IBD)
PW2	TNF	Inflammatory bowel disease (IBD)
PW3	MET	Transcriptional misregulation in cancer
PW3	MMP3	Transcriptional misregulation in cancer
PW3	MMP9	Transcriptional misregulation in cancer
PW3	PPARG	Transcriptional misregulation in cancer
PW4	AKT1	Hepatitis B
PW4	BCL2	Hepatitis B
PW4	CDK2	Hepatitis B
PW4	CDK4	Hepatitis B
PW4	EP300	Hepatitis B
PW4	MMP9	Hepatitis B
PW4	RAF1	Hepatitis B
PW4	STAT3	Hepatitis B
PW4	TLR4	Hepatitis B
PW4	TNF	Hepatitis B
PW5	AKT1	Chagas disease (American trypanosomiasis)
PW5	NOS2	Chagas disease (American trypanosomiasis)
PW5	SERPINE1	Chagas disease (American trypanosomiasis)
PW5	TLR4	Chagas disease (American trypanosomiasis)
PW5	TNF	Chagas disease (American trypanosomiasis)
PW5	TLR9	Chagas disease (American trypanosomiasis)
PW6	EGFR	Epithelial cell signaling in Helicobacter pylori infection
PW6	CXCR2	Epithelial cell signaling in Helicobacter pylori infection
PW6	MET	Epithelial cell signaling in Helicobacter pylori infection
PW6	ADAM17	Epithelial cell signaling in Helicobacter pylori infection
PW7	AKT1	Longevity regulating pathway
PW7	PPARA	Longevity regulating pathway
PW7	RPS6KB1	Longevity regulating pathway
PW7	STAT3	Longevity regulating pathway
PW7	TNF	Longevity regulating pathway
PW7	NR1H2	Longevity regulating pathway
PW8	ALOX5	Serotonergic synapse
PW8	ALOX15	Serotonergic synapse
PW8	BRAF	Serotonergic synapse
PW8	CYP2C19	Serotonergic synapse
PW8	HTR1A	Serotonergic synapse
PW8	PTGS1	Serotonergic synapse
PW8	PTGS2	Serotonergic synapse
PW8	RAF1	Serotonergic synapse

PW8	SLC6A4	Serotonergic synapse
PW9	AKT1	TNF signaling pathway
PW9	MMP3	TNF signaling pathway
PW9	MMP9	TNF signaling pathway
PW9	MMP14	TNF signaling pathway
PW9	PTGS2	TNF signaling pathway
PW9	TNF	TNF signaling pathway
PW10	AKT1	T cell receptor signaling pathway
PW10	CDK4	T cell receptor signaling pathway
PW10	LCK	T cell receptor signaling pathway
PW10	RAF1	T cell receptor signaling pathway
PW10	TNF	T cell receptor signaling pathway
PW11	EP300	TGF-beta signaling pathway
PW11	RPS6KB1	TGF-beta signaling pathway
PW11	TNF	TGF-beta signaling pathway
PW12	AKT1	Longevity regulating pathway
PW12	PPARG	Longevity regulating pathway
PW12	RPS6KB1	Longevity regulating pathway
PW13	CDK1	p53 signaling pathway
PW13	CDK2	p53 signaling pathway
PW13	CDK4	p53 signaling pathway
PW13	CHEK1	p53 signaling pathway
PW13	SERPINE1	p53 signaling pathway
PW14	AKT1	Endocrine resistance
PW14	BCL2	Endocrine resistance
PW14	BRAF	Endocrine resistance
PW14	CDK4	Endocrine resistance
PW14	EGFR	Endocrine resistance
PW14	ESR1	Endocrine resistance
PW14	ESR2	Endocrine resistance
PW14	MMP9	Endocrine resistance
PW14	RAF1	Endocrine resistance
PW14	RPS6KB1	Endocrine resistance
PW	PW1	
PW	PW2	
PW	PW3	
PW	PW4	
PW	PW5	
PW	PW6	
PW	PW7	
PW	PW8	
PW	PW9	
PW	PW10	
PW	PW11	
PW	PW12	
PW	PW13	
PW	PW14	
